# Supplementary material for: Linkage of Maternity Hospital Episode Statistics data to birth registration and notification records for births in England 2005–2014: Quality assurance of linkage of routine data for singleton and multiple births
Source: BMJ Open. 2018 Mar 1;8(3):e017898. doi: 10.1136/bmjopen-2017-017898 (PMC5855305; doi:10.1136/bmjopen-2017-017898)
Supplement: Supplementary file 1 [file bmjopen-2017-017898supp001.pdf]

Appendix A

Quality assurance procedures for singleton and multiple births to select only one correct linked HES delivery record for each ONS birth record and discard incorrect linkages (detailed version)

Key: SMDB – same mother, different baby; SMSB – same mother, same baby

1: Singleton births

RULE	CATEGORY	APPLIES TO	K (Keep the link)	D (Discard the link)	NOTES
1	Cleaning	Linked to HES records that have no birth or op dates or is an invalid delivery		HES records where baby date of birth = null or valid delivery = false	
2	Cleaning	Duplicate HES records (typically x 6 or x 9) linked to same ONS birth record where baby tail information and identifier is identical	The first version of the duplicate HES record goes through to the subsequent cleaning stages	All other versions of the duplicate HES record	Due to IT administrative issue with some NHS Trusts
3	SMDB	ONS birth record linked to HES record where baby date of birth differs between them by 24 weeks (168 days) or more		If HES baby date of birth > 24 weeks (168 days) from ONS birth record baby date of birth	The size of the date difference implies a different birth to the same mother<br><br>Kings Project used 30 weeks
4	SMSB	ONS birth record linked to one HES record (originally or remaining)	If ONS birth and HES variables match as listed in Box 3	If ONS birth and HES variables do not match as listed in Box 3	
5	SMSB	ONS birth record linked to >1 HES record all from same spell with genuine epiorder sequence (or a combination of genuine spell and additional delivery records)	If ONS birth and HES variables match as listed in Box 3 (baby date of birth must be exact match) and episode is part of spell and onset of delivery and delivery method and birth status values are valid	If ONS birth and HES variables do not match as listed in Box 3 or are the other episodes in that spell	Identifies where ONS birth records are linked to >1 HES record because some or all form a hospital spell. A spell is identified as where episodes have a sequence of epiorder numbers (e.g. 1 and 2) and the episode start date of the subsequent episode = the episode end date of the previous episode, all have the same admission date, and the discharge date is only recorded on the final episode.<br><br>The episode in the spell with the maximum and valid delivery information is chosen

6	SMSB	ONS birth record linked to >1 HES record with different epikeys but duplicate (admidate, disdate, epistart, epiend, epiorder, date of birth, gestat, birweit, baby sex, numbaby, birstat, birordr, delonset, delmeth, ICD code count, OP code count, procode, sitetret)	If ONS birth and HES variables match as listed in Box 3 and highest epikey	If ONS birth and HES variables do not match as listed in Box 3 or if not highest epikey	Highest epikey is the latest HES record and should contain the most information
7	SMSB	ONS birth record linked to >1 HES record with different epikeys but duplicate (epiorder, date of birth, gestat, birweit, baby sex, numbaby, birstat, birordr, delonset, delmeth, procode, sitetret)<br><br>Different admidate, disdate, epistart, epiend, ICD code count, OP code count	If ONS birth and HES variables match as listed in Box 3 and highest epikey	If ONS birth and HES variables do not match as listed in Box 3 or if not highest epikey	Highest epikey is the latest HES record and should contain the most information<br><br>The additional HES records can be pre- or post- hospital admissions relating to a delivery
8	SMSB	ONS birth record linked to >1 HES record with different epikeys not duplicates	If ONS birth and HES variables match as listed in Box 3 (baby date of birth must be exact match) and ONS and onset of delivery and delivery method and birth status and birth order values are valid	If ONS birth and HES variables match as listed in Box 3 but baby date of birth is not an exact match or HES baby date of birth is null) and onset of delivery and delivery method and birth status and birth order values are not valid and there is a corresponding K record	Chooses the linked HES record with the maximum valid delivery information
10	SMSB	ONS birth record linked to 2 HES records, both match on baby date of birth and location of birth	If ONS birth and HES variables match as listed in Box 3 (HES derived and original baby date of birth must be exact match to ONS) and onset of delivery and delivery method and birth status and birth order values are valid. Most escalated onset of delivery and delivery method	If ONS birth and HES variables match as listed in Box 3 (HES derived baby date of birth is exact match to ONS but HES original baby date of birth is null) and onset of delivery and delivery method and birth status and birth order values are valid (but less escalated)	A small number of records that can be differentiated by one containing maximum delivery information and one doesn’t where they both match on location of birth and baby dob

11	SMSB	ONS birth record linked to HES records with differing baby date of birth values (within 14 days of ONS baby date of birth)	If ONS birth and HES variables match as listed in Box 3 (baby date of birth must be exact match) and valid onset of delivery and delivery method value	If HES baby date of birth <=-14 days from ONS baby date of birth or HES baby date of birth <=-1 and >-14 days before ONS baby date of birth<br><br>or<br><br>if already a K record and HES baby date of birth >=1-14 days from ONS baby date of birth	Differentiates between the HES linked records by HES baby date of birth. The HES records that have HES baby date of birth <=-14 days from ONS baby date of birth, then <=-1 and >-14 days before ONS baby date of birth, then >=1-14 days from ONS baby date of birth, are discarded and the remaining 1 linked HES record with HES baby date of birth = ONS baby date of birth and valid method of delivery value is kept (if matching).<br><br>The discarded HES records look like maternal pregnancy care records based on ICD codes
12	SMDB	ONS birth record linked to HES records with differing baby date of birth values (within 15 to 168 days of ONS baby date of birth)	If ONS birth and HES variables match as listed in Box 3 (baby date of birth must be exact match) and valid onset of delivery and delivery method value	If already a K record and HES baby date of birth >14-168 days from ONS baby date of birth and different baby tail values	The discarded records look like different births approximately 2 or 3 months later to the same mother, some of which are clearly premature births.<br><br>Could have been incorporated in Rule 3
13		ONS birth record linked to >1 HES record with different epikeys but duplicate (date of birth, gestat, birweit, baby sex, delonset, delmeth)<br><br>]]	If ONS birth and HES variables match as listed in Box 3 and highest epikey	If not highest epikey	
14	SMSB	Clerical check to identify potential multiple birth records where >1 HES baby tail with same epikey linking to ONS birth record and/or where HES multibirth ICD is true and/or the epikey is linked to >1 ONS birth record, but not due to SMDB	Keep as a singleton matched birth if ONS birth and HES variables match as listed in Box 3<br><br>and<br><br>HES multiple birth ICD but matches only one ONS birth record<br><br>or<br><br>Either ONS birth or HES say multiple or stillbirth, baby tails identical, keep only 1 HES record with field order 1	Identify as potential multiple birth if:<br><br>epikey links >1 ONS birth record with same baby date of birth<br><br>or<br><br>2 different HES baby tails, 1 still birth, and only 1 ONS birth record for livebirth<br><br>or<br><br>HES multiple birth ICD and HES	Any records identified as potential multiple births are separated and given to ONS to check

			(genuine duplicates)<br><br>or<br><br>ONS birth singleton and HES multiple, treat as singleton if baby tails are the same	baby tails different<br><br>D all if ONS birth and HES variables do not match as listed in Box 3	
15	Check	If previous stages have kept > correct number of linked HES records per ONS birth	The highest epikey	Version (s) with earlier or other epikeys	
16	Check	If previous stages have kept no links per ONS birth	If birth location match, ONS baby date of birth within HES admission date ranges, and gestation, birth weight and sex match or are null and match rank 1 and is not a SMDB or invalid record.<br><br>Or if birth location does not match but everything else does		Clerical

2: Multiple Births

RULE	CATEGORY	APPLIES TO	K (Keep the link)	D (Discard the link)	NOTES
1	Cleaning	Linked to HES records that are invalid deliveries		HES records where valid delivery = false	Do not exclude those where HES baby date of birth = null because may be ghost baby tail records
2	Cleaning	Duplicate HES records (typically x 6 or x 9) linked to same ONS birth record where baby tail information and identifier is identical	The first version of the duplicate HES record goes through to the subsequent cleaning stages	All other versions of the duplicate HES record	Due to IT administrative issue with some NHS Trusts
3	SMDB	ONS birth record linked to HES record where baby date of birth differs between them by 24 weeks (168 days) or more		If HES baby date of birth > 24 weeks (168 days) from ONS birth record baby date of birth	The size of the date difference implies a different birth to the same mother<br><br>Kings Project used 30 weeks
4	SMSB	ONS birth records in MULTBTNO linked to one epikey and the correct number of baby tails (originally or remaining)<br><br>Correct number of baby tails<br><br>Correct number of ONS birth records<br><br>1 epikey per MULTBTNO	If ONS birth and HES variables match as listed in Box 3 and field order 1 for 1st baby, field order 2 for 2nd baby etc. so that ONS birth record corresponds to correct baby tail	If ONS birth and HES variables do not match as listed in Box 3.<br><br>Or field and birth orders don't match and sex and birth weight are wrong way round and already 2 correct K records	
5	SMSB	ONS birth records in MULTBTNO linked to one epikey and more than the correct number of baby tails<br><br>> correct number of baby tails<br><br>(duplicates)	If ONS birth and HES variables match as listed in Box 3 and field order 1 (or lowest) for 1st baby, field order 2 (or plus 1) for 2nd baby etc. so that birth record corresponds to correct baby tail	If ONS birth and HES variables do not match as listed in Box 3.<br><br>Or field and birth orders don't match and sex and birth weight are wrong way round and already 2 correct K records	

		Correct number of ONS birth records<br>1 epikey per MULTBTNO			
6	SMSB	ONS birth records in MULTBTNO linked to more than one epikey<br>Correct number of ONS birth records<br>>1 epikey per MULTBTNO	Where epikey matches by baby date of birth, ONS baby date of birth is within HES admission date range, and has valid/most escalated onset of delivery and delivery method	All others	Births with >1 epikey correctly associated with them, but not spells<br>Due to first baby linked to 1 epikey and the second baby to the other or duplicates with epikey number increasing by one each time, or non-delivery records
7	SMSB	Remaining MULTBTNO with < correct number of ONS birth records	If ONS birth and HES variables match as listed in Box 3		Some of the missing ONS birth records found in singleton file and unlinked file
8	Check	If previous stages have kept > correct number of links per MULTBTNO. Epikeys are exact duplicates	Version with highest epikey or version within admission date range	Version (s) with earlier or other epikeys	
9	Check	If previous stages have kept no links per MULTBTNO	If birth location match, ONS baby date of birth within HES admission date ranges, and gestation, birth weight and sex match or are null and match rank 1 and is not a SMDB or invalid record<br>Or if birth location does not match but everything else does		Clerical

Appendix B

Differences between records with correct and incorrect linkage

Comparison of distributions of key variables for singleton and multiple births in England 2005 to 2014, for 4 groups of data:

1) All birth registrations (n = 6,468,586 singleton births, n = 208,326 multiple births)

2) All birth registrations linked to HES by NHS Digital with a HES delivery record (n = 6,268,013 singleton births, n = 196,769 multiple births)

3) Those of 2) that remain linked to HES after QA (n = 6,138,487 singleton births, n = 193,270 multiple births)

4) Those of 2) left with no link after QA (n = 129,526 singleton births, n = 3,499 multiple births)

Tables B.13 and B.14 are based on total links (a birth registration record can link to >1 HES record):

1) All birth registrations (n = 6,468,586 singleton births, n = 208,326 multiple births)

2) All birth registrations and HES links by NHS Digital (n = 7,862,903 singleton births, n = 395,202 multiple births)

3) Those of 2) that remain linked to HES after QA (n = 6,138,487 singleton births, n = 193,270 multiple births)

4) Those of 2) total broken links after QA (n = 1,724,416 singleton births, n = 201,932 multiple births)

Total number of links are inflated for multiple births because each birth registration within a multiple delivery links with every possible HES baby tail i.e. 4 linked records minimum

Chi-square tests are conducted to test the null hypothesis that the distribution of a demographic variable is the same in the "linked to HES after QA" as in the "No link to HES after QA"

Any rows with values of 0 are excluded from the Chi-square tests

	Singleton births England				Multiple births England				Difference between no link to HES delivery and linked to HES delivery after QA	
	Percentage distribution of variables				Percentage distribution of variables				Singletons	Multiples
**B.1: Match rank**	**All birth registrations**	**All birth registrations linked to HES by NHS Digital with a HES delivery record**	**Linked to HES delivery after QA**	**No link to HES delivery after QA**	**All birth registrations**	**All birth registrations linked to HES by NHS Digital with a HES delivery record**	**Linked to HES delivery after QA**	**No link to HES delivery after QA**		
1	80.51	81.92	82.06	75.13	80.3	81.4	81.4	80.9	-6.93	-0.59
2	2.65	2.67	2.65	3.68	2.0	2.1	2.1	2.8	1.03	0.71
3	1.39	1.42	1.42	1.27	1.3	1.3	1.3	1.1	-0.15	-0.16
4	0.06	0.06	0.06	0.09	0.0	0.0	0.0	0.1	0.03	0.08
5	0.45	0.46	0.46	0.51	0.4	0.4	0.4	0.5	0.06	0.14
6	13.26	13.16	13.05	18.40	14.3	14.4	14.4	13.6	5.35	-0.85
7	0.28	0.27	0.27	0.38	0.3	0.3	0.3	0.1	0.11	-0.25
8	0.05	0.04	0.03	0.53	0.0	0.0	0.0	0.9	0.50	0.93
Null	1.36	0.00	0.00	0.00	1.3	0.0	0.0	0.0	0.00	0.00
**Total**	6,468,586	6,268,013	6,138,487	129,526	208,326	196,769	193,270	3,499		
**χ^2 test**	$p < .001$ (χ^2 = 11815.13 df = 7)				$p < .001$ (χ^2 = 903.69, df = 7)					
**B.2: Region of birth**										
East Midlands	6.84	6.95	7.02	3.56	6.61	6.66	6.64	7.66	-3.46	1.02
East of England	10.05	10.08	10.12	8.09	10.50	10.46	10.43	12.35	-2.03	1.92
London	19.36	18.92	18.93	18.34	21.91	21.14	21.03	26.86	-0.59	5.83
North East	4.61	4.66	4.73	0.97	4.62	4.75	4.81	1.54	-3.77	-3.26
North West	13.02	13.18	13.32	6.63	12.35	12.49	12.52	10.89	-6.69	-1.63
South Central	6.92	7.03	7.08	4.56	7.39	7.47	7.47	7.69	-2.52	0.22
South East Coast	7.68	7.76	7.83	4.38	8.48	8.53	8.52	8.72	-3.45	0.19
South West	8.48	8.63	8.73	3.74	8.91	9.17	9.25	4.74	-5.00	-4.51
West Midlands	10.64	10.78	10.93	3.83	10.03	10.05	10.09	7.75	-7.09	-2.35

Yorkshire/Humber	9.69	9.85	9.97	4.07	8.92	9.05	9.07	8.46	-5.90	-0.61
Elsewhere	0.17	0.15	0.10	2.34	0.05	0.04	0.04	0.43	2.24	0.39
Home	2.54	2.01	1.22	39.49	0.23	0.19	0.14	2.92	38.27	2.77
Not known	0.00	0.00	0.00	0.00	0.00	0.00	0	0	0.00	0.00
**Total**	6,468,586	6,268,013	6,138,487	129,526	208,326	196,769	193,270	3,499		
**χ^2 test**	p < .001 (χ^2 = 992568.53, df = 11)				p < .001 (χ^2 = 1768.25, df = 11)					
**B.3: Year of birth**										
2005	9.26	9.02	9.03	8.49	8.82	8.84	8.90	5.57	-0.55	-3.33
2006	9.60	9.40	9.35	11.86	9.38	9.41	9.45	7.55	2.51	-1.90
2007	9.88	9.78	9.70	13.28	9.63	9.79	9.81	8.57	3.57	-1.23
2008	10.14	10.14	10.12	11.12	9.99	10.22	10.29	5.89	1.00	-4.41
2009	10.10	10.15	10.12	11.47	10.56	10.84	10.91	7.23	1.35	-3.68
2010	10.29	10.41	10.45	8.78	10.32	10.69	10.79	5.32	-1.67	-5.47
2011	10.30	10.43	10.46	8.72	10.61	10.77	10.80	9.35	-1.75	-1.45
2012	10.46	10.55	10.56	10.04	10.59	10.25	10.12	17.49	-0.52	7.37
2013	10.01	10.10	10.15	7.94	9.97	9.52	9.40	16.29	-2.21	6.89
2014	9.95	10.02	10.06	8.31	10.13	9.66	9.54	16.75	-1.74	7.21
**Total**	6,468,586	6,268,013	6,138,487	129,526	208,326	196,769	193,270	3,499		
**χ^2 test**	p < .001 (χ^2 = 4645.84, df = 9)				p < .001 (χ^2 = 811.59, df = 9)					
**B.4: Month of birth**										
Jan	8.28	8.27	8.26	8.46	8.35	8.38	8.39	8.09	0.20	-0.30
Feb	7.59	7.58	7.56	8.40	7.57	7.56	7.57	6.97	0.84	-0.60
March	8.29	8.26	8.21	10.19	8.22	8.20	8.17	9.89	1.98	1.72
April	8.00	7.99	8.00	7.56	7.95	7.97	7.94	9.43	-0.44	1.49
May	8.43	8.44	8.45	7.72	8.38	8.38	8.39	7.92	-0.74	-0.48
June	8.27	8.28	8.29	7.91	8.55	8.52	8.52	8.57	-0.38	0.06
July	8.68	8.69	8.70	8.10	8.68	8.70	8.70	8.92	-0.60	0.22
Aug	8.60	8.61	8.62	8.17	8.07	8.14	8.15	7.57	-0.45	-0.58
Sep	8.65	8.67	8.68	8.21	8.39	8.43	8.46	6.89	-0.47	-1.57
Oct	8.67	8.69	8.70	8.24	8.97	8.91	8.91	9.03	-0.46	0.12
Nov	8.21	8.22	8.22	8.08	8.43	8.40	8.41	7.89	-0.14	-0.53
Dec	8.32	8.32	8.30	8.95	8.44	8.39	8.38	8.83	0.65	0.45

**Total**	6,468,586	6,268,013	6,138,487	129,526	208,326	196,769	193,270	3,499		
**χ^2 test**	p < .001 (χ^2 = 1072.06, df = 11)				p < .001 (χ^2 = 42.23, df = 11)					
**B.5: Day of birth (8)**										
Mon	13.25	13.25	13.25	13.27	13.75	13.76	13.74	14.49	0.02	0.75
Tue	14.63	14.64	14.64	14.53	16.41	16.38	16.39	15.72	-0.11	-0.67
Wed	14.90	14.90	14.90	14.86	16.74	16.80	16.80	16.29	-0.04	-0.51
Thur	15.03	15.03	15.04	14.87	17.31	17.29	17.29	17.63	-0.17	0.35
Fri	14.73	14.73	14.73	14.65	16.66	16.65	16.68	15.06	-0.08	-1.62
Sat	12.96	12.95	12.94	13.32	9.77	9.76	9.74	10.95	0.38	1.21
Sun	12.14	12.15	12.15	12.10	7.75	7.75	7.75	8.00	-0.05	0.25
Hol	2.36	2.36	2.36	2.40	1.61	1.62	1.61	1.86	0.04	0.25
**Total**	6,468,586	6,268,013	6,138,487	129,526	208,326	196,769	193,270	3,499		
**χ^2 test**	p < .001 (χ^2 = 36.20, df = 7)				p = .0333 (χ^2 = 15.22, df = 7)					
**B.6: Day of birth (11)**										
Mon	13.05	13.05	13.05	12.99	13.54	13.55	13.54	14.32	-0.07	0.78
Tue	13.32	13.33	13.33	13.26	14.95	14.92	14.93	14.58	-0.06	-0.35
Wed	14.59	14.59	14.59	14.46	16.37	16.43	16.44	16.00	-0.12	-0.43
Thur	14.55	14.56	14.56	14.24	16.78	16.76	16.76	16.95	-0.32	0.19
Fri	13.59	13.59	13.59	13.55	15.39	15.37	15.40	13.63	-0.04	-1.77
Sat	12.96	12.95	12.94	13.47	9.77	9.76	9.74	10.95	0.53	1.21
Sun	12.14	12.15	12.15	12.24	7.75	7.75	7.75	8.00	0.09	0.25
a.hol	1.68	1.68	1.69	1.62	1.89	1.88	1.88	1.60	-0.07	-0.28
b.hol	1.75	1.75	1.75	1.72	1.95	1.96	1.96	2.11	-0.03	0.16
Christmas	0.43	0.43	0.43	0.50	0.29	0.29	0.29	0.37	0.07	0.09
Hol	1.93	1.93	1.93	1.95	1.33	1.33	1.33	1.49	0.03	0.16
**Total**	6,468,586	6,268,013	6,138,487	129,526	208,326	196,769	193,270	3,499		
**χ^2 test**	p < .001 (χ^2 = 60.47, df = 10)				p = .0525 (χ^2 = 18.15, df = 10)					
**B.7: Hour of birth**										
0.00 - 2.59	12.35	12.36	12.35	12.84	8.42	8.46	8.45	9.00	0.49	0.56
3.00 - 5.59	12.15	12.15	12.12	13.75	7.74	7.82	7.81	8.06	1.64	0.25
6.00 - 8.59	10.79	10.76	10.73	12.53	6.67	6.69	6.67	8.03	1.80	1.36

9.00 -11.59	16.06	16.10	16.13	14.39	26.62	26.56	26.53	27.98	-1.74	1.45
12.00 -14.59	12.88	12.87	12.90	11.80	17.35	17.27	17.28	16.95	-1.10	-0.33
15.00 -17.59	11.86	11.85	11.87	10.93	13.41	13.37	13.40	11.77	-0.94	-1.63
18.00 -20.59	11.19	11.18	11.19	11.02	9.65	9.65	9.67	8.49	-0.16	-1.19
21.00 -23.59	11.81	11.81	11.81	11.80	9.18	9.21	9.22	8.69	-0.01	-0.53
Null	0.91	0.91	0.91	0.93	0.96	0.96	0.96	1.03	0.03	0.07
**Total**	6,468,586	6,268,013	6,138,487	129,526	208,326	196,769	193,270	3,499		
**χ^2 test**	p < .001 (χ^2 = 1146.18, df = 8)				p = < .001 (χ^2 = 26.99, df = 8)					
**B.8: Age of mother**										
Under 15	0.03	0.03	0.03	0.02	0.01	0.01	0.01	0.00	-0.01	-0.01
15-19	3.16	3.16	3.18	2.28	1.24	1.26	1.27	0.86	-0.89	-0.41
20-24	18.48	18.57	18.59	17.39	11.14	11.41	11.46	8.75	-1.20	-2.72
25-29	27.29	27.42	27.42	27.44	22.70	23.10	23.19	18.12	0.01	-5.07
30-34	28.72	28.73	28.70	30.39	32.75	32.85	32.83	33.98	1.69	1.16
35-39	16.13	15.99	15.96	17.20	23.75	23.44	23.40	25.81	1.24	2.41
40-44	3.60	3.53	3.54	3.19	5.99	5.64	5.57	9.46	-0.35	3.89
45 & more	0.20	0.19	0.19	0.19	1.34	1.17	1.15	2.14	0.00	0.99
Null	2.39	2.39	2.40	1.91	1.08	1.12	1.12	0.89	-0.49	-0.24
**Total**	6,468,586	6,268,013	6,138,487	129,526	208,326	196,769	193,270	3,499		
**χ^2 test**	p < .001 (χ^2 = 716.36, df = 8)				p < .001 (χ^2 = 196.19, df = 8)					
**B.9: Sex of baby**										
Female	48.69	48.69	48.66	49.65	49.87	49.91	49.93	48.84	0.99	-1.09
Male	51.31	51.31	51.34	50.35	50.13	50.09	50.07	51.16	-0.99	1.09
**Total**	6,468,586	6,268,013	6,138,487	129,526	208,326	196,769	193,270	3,499		
**χ^2 test**	p < .001 (χ^2 = 49.40, df = 1)				p = .2018 (χ^2 = 1.63, df = 1)					
**B.10: Ethnicity of baby**										
Bangladeshi	1.41	1.43	1.43	1.00	0.86	0.89	0.89	0.69	-0.43	-0.20
Indian	3.00	3.02	3.04	1.90	2.73	2.62	2.60	3.63	-1.14	1.03
Pakistani	4.12	4.15	4.18	2.81	2.98	2.99	2.98	3.06	-1.37	0.07
Black African	3.21	3.14	3.12	3.90	3.73	3.67	3.64	5.54	0.77	1.91
Black Caribbean	0.97	0.97	0.97	0.91	1.09	1.09	1.10	0.97	-0.06	-0.13

White British	63.44	63.99	63.97	64.75	65.11	65.85	65.90	62.79	0.78	-3.12
White Other	8.19	8.10	8.13	6.75	8.38	8.21	8.23	7.14	-1.38	-1.08
Other	9.43	9.34	9.35	8.71	8.85	8.65	8.65	8.69	-0.64	0.03
Not Known	6.20	5.87	5.80	9.21	6.26	6.02	5.99	7.40	3.41	1.41
Null	0.02	0.01	0.01	0.07	0.02	0.01	0.01	0.09	0.06	0.08
**Total**	6,468,586	6,268,013	6,138,487	129,526	208,326	196,769	193,270	3,499		
**χ^2 test**	p < .001 (χ^2 = 4730.98 df = 9)				p < .001 (χ^2 = 151.29, df = 9)					
**B.11: Gestational age**										
Missing or less than 22 weeks	0.79	0.78	0.76	1.29	0.93	0.92	0.90	1.83		
									0.53	0.92
Pre-term	6.08	6.07	6.05	7.13	54.10	53.86	53.84	54.93	1.08	1.09
Term	89.17	89.18	89.19	88.63	44.85	45.09	45.12	43.13	-0.56	-2.00
Post-term	3.96	3.97	4.00	2.95	0.13	0.13	0.13	0.11	-1.04	-0.01
**Total**	6,468,586	6,268,013	6,138,487	129,526	208,326	196,769	193,270	3,499		
**χ^2 test**	p < .001 (χ^2 = 1048.57, df = 3)				p < .001 (χ^2 = 35.71, df = 3)					
**B.12: Stillbirth**										
No	99.52	99.53	99.58	97.30	98.87	98.88	98.92	96.46	-2.28	-2.47
Yes	0.48	0.47	0.42	2.70	1.13	1.12	1.08	3.54	2.28	2.47
**Total**	6,468,586	6,268,013	6,138,487	129,526	208,326	205,684	193,270	12,414		
**χ^2 test**	p < .001 (χ^2 = 14053.04, df = 1)				p < .001 (χ^2 = 188.14, df = 1)					
**B.13: Gestational age missing on HES**	**All birth registrations**	**All birth registrations and HES links by NHS Digital**	**Linked to HES delivery after QA**	**Broken links after QA**	**All birth registrations**	**All birth registrations and HES links by NHS Digital**	**Linked to HES delivery after QA**	**Broken links after QA**		
No		68.94	73.50	52.70		74.18	79.73	68.86	-20.80	-10.87
Yes		31.06	26.50	47.30		25.82	20.27	31.14	20.80	10.87
**Total**	6,468,586	7,862,903	6,138,487	1,724,416	208,326	196,769	193,270	3,499		
**χ^2 test**	p < .001 (χ^2 = 272051.09 df = 1)				p < .001 (χ^2 = 6086.08, df = 1)					
**B.14: Birth weight missing on HES**										

No		80.69	83.38	71.12		85.88	89.52	82.40	-12.26	-7.13
Yes		19.31	16.62	28.88		14.12	10.48	17.60	12.26	7.13
**Total**	6,468,586	7,862,903	6,138,487	1,724,416	208,326	395,202	193,270	201,932		
**χ^2 test**	$p < .001$ (χ^2 = 129812.25 df = 1)				$p < .001$ (χ^2 = 4814.69, df = 1)					

	**Singleton births England** Percentage distribution of variables				**Multiple births England** Percentage distribution of variables					
**B.15: Location of birth (assigned trust code)**	All birth registrations	All birth registrations linked to HES by NHS Digital with a HES delivery record	Linked to HES delivery after QA	No link to HES delivery after QA	All birth registrations	All birth registrations linked to HES by NHS Digital with a HES delivery record	Linked to HES delivery after QA	No link to HES delivery after QA		
**MMC**	0.00	0.00	0.00	0.00	0.00	0.00	0.00	0.00	0.00	0.00
**MML**	0.07	0.00	0.00	0.04	0.04	0.00	0.00	0.00	0.04	0.00
**MMS**	0.00	0.00	0.00	0.00	0.00	0.00	0.00	0.00	0.00	0.00
**PPJ**	0.04	0.01	0.00	0.26	0.00	0.00	0.00	0.06	0.26	0.06
**PPL**	0.30	0.04	0.00	1.74	0.40	0.05	0.00	2.52	1.74	2.51
**PPN**	0.00	0.00	0.00	0.01	0.00	0.00	0.00	0.00	0.01	0.00
**PPT**	0.00	0.00	0.00	0.08	0.00	0.00	0.00	0.00	0.08	0.00
**R1F**	0.18	0.18	0.18	0.27	0.18	0.18	0.18	0.31	0.09	0.13
**RA2**	0.48	0.49	0.49	0.21	0.57	0.59	0.60	0.23	-0.28	-0.37
**RA4**	0.22	0.23	0.23	0.11	0.18	0.19	0.18	0.37	-0.12	0.19
**RA7**	0.84	0.86	0.87	0.35	0.84	0.89	0.90	0.23	-0.52	-0.67
**RA9**	0.35	0.35	0.35	0.10	0.33	0.33	0.33	0.11	-0.25	-0.22
**RAE**	0.89	0.91	0.92	0.43	0.66	0.69	0.68	1.00	-0.49	0.32
**RAJ**	0.54	0.55	0.55	0.24	0.57	0.56	0.55	0.86	-0.31	0.31
**RAL**	0.48	0.48	0.49	0.13	0.44	0.44	0.44	0.34	-0.36	-0.10
**RAP**	0.55	0.55	0.55	0.21	0.49	0.49	0.49	0.34	-0.34	-0.15
**RAS**	0.60	0.61	0.62	0.15	0.57	0.59	0.59	0.40	-0.47	-0.19
**RAX**	0.83	0.84	0.85	0.32	0.99	0.93	0.93	1.23	-0.52	0.30
**RBA**	0.47	0.47	0.48	0.27	0.55	0.55	0.55	0.46	-0.21	-0.09
**RBD**	0.30	0.31	0.32	0.04	0.31	0.32	0.33	0.14	-0.28	-0.18

**RBK**	0.61	0.63	0.64	0.13	0.50	0.51	0.51	0.29	-0.50	-0.23
**RBL**	0.52	0.53	0.54	0.14	0.52	0.53	0.53	0.26	-0.40	-0.28
**RBN**	0.50	0.49	0.50	0.08	0.41	0.41	0.41	0.06	-0.43	-0.36
**RBT**	0.42	0.42	0.42	0.45	0.39	0.39	0.39	0.37	0.02	-0.02
**RBZ**	0.23	0.24	0.24	0.10	0.21	0.22	0.22	0.29	-0.14	0.07
**RC1**	0.46	0.46	0.46	0.39	0.48	0.46	0.46	0.60	-0.07	0.14
**RC3**	0.43	0.40	0.40	0.09	0.35	0.33	0.33	0.40	-0.32	0.07
**RC9**	0.74	0.75	0.76	0.50	0.81	0.80	0.80	0.66	-0.25	-0.15
**RCB**	0.49	0.50	0.51	0.05	0.48	0.51	0.51	0.34	-0.46	-0.17
**RCC**	0.25	0.25	0.23	1.04	0.17	0.17	0.16	0.69	0.81	0.52
**RCD**	0.29	0.30	0.31	0.04	0.25	0.26	0.27	0.00	-0.27	-0.27
**RCF**	0.36	0.37	0.38	0.08	0.31	0.32	0.32	0.06	-0.30	-0.27
**RCX**	0.34	0.34	0.35	0.05	0.33	0.34	0.34	0.06	-0.30	-0.29
**RD1**	0.72	0.72	0.72	0.43	0.74	0.75	0.76	0.34	-0.29	-0.42
**RD3**	0.72	0.73	0.74	0.18	0.72	0.74	0.75	0.26	-0.57	-0.49
**RD7**	0.75	0.76	0.78	0.16	0.69	0.69	0.68	0.74	-0.61	0.06
**RD8**	0.55	0.55	0.54	1.02	0.52	0.51	0.50	1.23	0.48	0.73
**RDD**	0.64	0.66	0.67	0.11	0.61	0.64	0.65	0.14	-0.57	-0.51
**RDE**	0.56	0.57	0.57	0.31	0.58	0.58	0.57	0.80	-0.26	0.23
**RDU**	0.72	0.74	0.75	0.31	0.79	0.80	0.80	0.69	-0.43	-0.11
**RE9**	0.22	0.23	0.23	0.05	0.16	0.17	0.17	0.06	-0.18	-0.11
**REF**	0.62	0.64	0.64	0.30	0.59	0.61	0.62	0.34	-0.34	-0.27
**REP**	1.22	1.24	1.26	0.20	1.59	1.64	1.66	0.74	-1.07	-0.91
**RF4**	1.35	1.32	1.35	0.34	1.35	1.31	1.31	1.09	-1.01	-0.23
**RFF**	0.42	0.43	0.43	0.11	0.37	0.38	0.39	0.11	-0.33	-0.27
**RFR**	0.42	0.42	0.42	0.12	0.41	0.41	0.41	0.46	-0.30	0.05
**RFS**	0.43	0.44	0.45	0.09	0.38	0.40	0.41	0.11	-0.36	-0.29
**RFW**	0.64	0.63	0.62	1.13	0.54	0.49	0.47	1.23	0.51	0.76
**RGC**	0.77	0.76	0.77	0.33	0.74	0.72	0.72	0.80	-0.43	0.08
**RGN**	0.64	0.65	0.66	0.11	0.60	0.60	0.60	0.63	-0.54	0.03
**RGP**	0.32	0.33	0.33	0.06	0.29	0.29	0.29	0.17	-0.27	-0.12
**RGQ**	0.54	0.52	0.48	2.48	0.53	0.49	0.46	1.94	2.00	1.48
**RGR**	0.37	0.38	0.39	0.04	0.35	0.36	0.36	0.17	-0.34	-0.19
**RGT**	0.80	0.82	0.84	0.08	1.05	1.10	1.11	0.11	-0.76	-1.00
**RH8**	0.58	0.59	0.60	0.16	0.65	0.67	0.68	0.40	-0.44	-0.28

**RHM**	0.85	0.86	0.87	0.45	0.86	0.87	0.88	0.34	-0.42	-0.54
**RHQ**	1.03	1.05	1.07	0.20	1.16	1.14	1.13	1.74	-0.87	0.62
**RHU**	0.86	0.87	0.88	0.30	0.95	0.99	1.00	0.40	-0.58	-0.60
**RHW**	0.83	0.85	0.86	0.39	0.95	0.99	0.99	0.74	-0.48	-0.25
**RJ1**	0.97	0.98	0.99	0.30	1.24	1.26	1.28	0.49	-0.70	-0.79
**RJ2**	0.55	0.53	0.53	0.46	0.52	0.53	0.53	0.29	-0.07	-0.25
**RJ6**	0.65	0.65	0.65	0.68	0.61	0.61	0.59	1.91	0.03	1.33
**RJ7**	0.74	0.75	0.76	0.26	0.99	1.00	1.01	0.77	-0.50	-0.24
**RJC**	0.40	0.41	0.41	0.14	0.36	0.36	0.36	0.29	-0.27	-0.07
**RJD**	0.31	0.31	0.32	0.08	0.28	0.27	0.28	0.06	-0.24	-0.22
**RJE**	0.85	0.86	0.87	0.29	0.77	0.79	0.80	0.31	-0.58	-0.48
**RJF**	0.54	0.54	0.54	0.46	0.48	0.50	0.50	0.34	-0.09	-0.16
**RJL**	0.69	0.70	0.71	0.46	0.59	0.61	0.61	0.57	-0.24	-0.04
**RJN**	0.30	0.31	0.31	0.02	0.30	0.31	0.32	0.09	-0.29	-0.23
**RJR**	0.47	0.48	0.49	0.08	0.47	0.48	0.49	0.11	-0.41	-0.37
**RJZ**	0.74	0.73	0.73	0.47	1.02	0.97	0.97	0.89	-0.26	-0.09
**RK5**	0.46	0.47	0.48	0.19	0.41	0.43	0.43	0.06	-0.29	-0.38
**RK9**	0.66	0.67	0.66	1.15	0.74	0.72	0.72	1.03	0.49	0.31
**RKB**	0.83	0.85	0.87	0.25	0.89	0.92	0.93	0.11	-0.62	-0.82
**RKE (Whittington)**	0.56	0.48	0.40	4.43	0.59	0.47	0.40	4.34	4.03	3.94
**RL4**	0.57	0.58	0.59	0.12	0.60	0.62	0.62	0.43	-0.47	-0.19
**RLN**	0.50	0.50	0.51	0.06	0.44	0.45	0.46	0.00	-0.45	-0.46
**RLQ**	0.27	0.28	0.29	0.07	0.26	0.25	0.25	0.29	-0.22	0.03
**RLT**	0.35	0.36	0.36	0.05	0.29	0.31	0.31	0.11	-0.32	-0.20
**RLU**	1.11	1.10	1.11	0.52	1.32	1.27	1.26	1.91	-0.58	0.66
**RM1**	0.84	0.86	0.88	0.04	0.82	0.86	0.87	0.20	-0.84	-0.67
**RM2**	0.56	0.56	0.55	1.04	0.55	0.52	0.50	1.43	0.49	0.93
**RM4**	0.11	0.11	0.11	0.24	0.08	0.08	0.08	0.17	0.13	0.09
**RMC**	0.74	0.76	0.78	0.13	0.61	0.63	0.64	0.11	-0.64	-0.53
**RMP**	0.40	0.41	0.41	0.12	0.31	0.32	0.32	0.17	-0.30	-0.15
**RN1**	0.41	0.42	0.43	0.16	0.44	0.44	0.44	0.51	-0.27	0.07
**RN3**	0.63	0.65	0.66	0.17	0.62	0.66	0.66	0.26	-0.49	-0.41
**RN5**	0.42	0.42	0.43	0.07	0.45	0.48	0.48	0.06	-0.36	-0.43
**RN7**	0.59	0.60	0.62	0.07	0.52	0.54	0.55	0.11	-0.54	-0.43
**RNA**	0.68	0.69	0.70	0.21	0.60	0.59	0.60	0.54	-0.50	-0.05

**RNH**	0.82	0.79	0.78	1.18	0.63	0.61	0.61	0.63	0.40	0.02
**RNJ**	0.68	0.69	0.70	0.14	0.57	0.57	0.57	0.49	-0.56	-0.09
**RNL**	0.47	0.46	0.46	0.51	0.42	0.41	0.41	0.66	0.05	0.25
**RNQ**	0.56	0.57	0.57	0.12	0.54	0.55	0.56	0.09	-0.45	-0.48
**RNS**	0.65	0.66	0.67	0.13	0.72	0.75	0.75	0.57	-0.54	-0.18
**RNZ**	0.34	0.35	0.35	0.08	0.43	0.45	0.45	0.06	-0.27	-0.40
**RP5**	0.79	0.81	0.82	0.13	0.70	0.71	0.71	0.71	-0.69	0.01
**RPA**	0.68	0.69	0.70	0.25	0.73	0.76	0.76	0.51	-0.45	-0.25
**RQ8**	0.64	0.64	0.62	1.30	0.70	0.65	0.63	2.17	0.68	1.55
**RQM**	0.81	0.81	0.82	0.49	1.15	1.11	1.11	1.00	-0.33	-0.11
**RQQ**	0.36	0.37	0.37	0.09	0.34	0.35	0.35	0.11	-0.28	-0.24
**RQW**	0.56	0.55	0.56	0.19	0.59	0.57	0.57	0.69	-0.37	0.12
**RQX**	0.74	0.74	0.73	1.02	0.97	0.97	0.97	0.86	0.29	-0.12
**RR1**	1.60	1.64	1.66	0.62	1.34	1.38	1.39	0.89	-1.04	-0.50
**RR7**	0.28	0.28	0.29	0.05	0.29	0.29	0.29	0.29	-0.24	0.00
**RR8**	1.40	1.38	1.39	0.64	1.47	1.43	1.43	1.34	-0.76	-0.08
**RRF**	0.45	0.45	0.46	0.20	0.42	0.43	0.43	0.40	-0.26	-0.03
**RRV**	0.74	0.75	0.76	0.37	1.35	1.35	1.36	0.94	-0.38	-0.42
**RTD**	0.97	0.98	1.00	0.26	1.25	1.29	1.30	0.57	-0.74	-0.73
**RTE**	0.93	0.95	0.97	0.11	0.98	1.03	1.04	0.17	-0.86	-0.87
**RTF**	0.47	0.48	0.49	0.08	0.34	0.35	0.36	0.00	-0.42	-0.36
**RTG**	0.84	0.86	0.87	0.32	0.79	0.79	0.78	1.17	-0.55	0.39
**RTH**	1.26	1.28	1.27	1.43	1.56	1.52	1.50	3.06	0.16	1.56
**RTK**	0.59	0.60	0.61	0.07	0.77	0.79	0.81	0.06	-0.54	-0.75
**RTP**	0.64	0.63	0.64	0.31	0.70	0.69	0.69	0.71	-0.33	0.02
**RTR**	0.80	0.80	0.81	0.20	0.91	0.93	0.95	0.17	-0.61	-0.77
**RTX**	0.51	0.52	0.52	0.25	0.41	0.42	0.43	0.23	-0.27	-0.20
**RV8**	0.74	0.75	0.76	0.27	0.61	0.59	0.60	0.49	-0.49	-0.11
**RVJ**	0.87	0.89	0.91	0.21	1.02	1.05	1.06	0.29	-0.70	-0.77
**RVL**	0.96	0.93	0.90	2.27	0.95	0.89	0.87	2.26	1.37	1.39
**RVR**	0.75	0.75	0.76	0.27	0.86	0.85	0.86	0.63	-0.49	-0.23
**RVV**	1.03	1.03	1.02	1.90	1.05	1.00	0.96	3.14	0.89	2.19
**RVW**	0.53	0.53	0.54	0.08	0.51	0.53	0.54	0.06	-0.46	-0.48
**RVY**	0.45	0.44	0.45	0.09	0.39	0.39	0.39	0.57	-0.37	0.18
**RW3**	1.27	1.27	1.28	0.93	1.48	1.43	1.41	2.52	-0.35	1.11

**RW6**	1.52	1.55	1.58	0.24	1.20	1.24	1.25	0.77	-1.34	-0.47
**RWA**	0.84	0.86	0.87	0.16	0.79	0.81	0.82	0.37	-0.71	-0.45
**RWD**	0.86	0.86	0.85	0.97	0.72	0.74	0.73	1.26	0.12	0.53
**RWE**	1.54	1.56	1.57	0.96	1.44	1.42	1.40	2.37	-0.61	0.97
**RWF**	0.76	0.77	0.78	0.33	0.98	1.01	1.02	0.51	-0.45	-0.50
**RWG**	0.82	0.84	0.85	0.33	0.91	0.89	0.88	1.03	-0.52	0.15
**RWH**	0.80	0.80	0.78	1.73	0.94	0.94	0.92	2.00	0.95	1.08
**RWJ**	0.54	0.56	0.57	0.08	0.52	0.55	0.55	0.40	-0.49	-0.15
**RWP**	0.87	0.87	0.89	0.28	0.89	0.83	0.84	0.71	-0.60	-0.12
**RWW**	0.48	0.49	0.50	0.22	0.44	0.45	0.46	0.23	-0.28	-0.23
**RWY**	0.86	0.87	0.88	0.43	0.78	0.79	0.79	0.86	-0.46	0.07
**RX1**	1.50	1.53	1.55	0.77	1.60	1.58	1.57	2.03	-0.78	0.46
**RXC**	0.58	0.59	0.60	0.13	0.49	0.49	0.49	0.54	-0.47	0.06
**RXF**	0.98	1.00	1.02	0.19	0.79	0.83	0.84	0.20	-0.83	-0.64
**RXH**	0.83	0.82	0.83	0.33	1.08	1.07	1.06	1.51	-0.50	0.45
**RXK**	0.89	0.89	0.90	0.43	0.68	0.67	0.66	1.00	-0.47	0.34
**RXL**	0.44	0.45	0.46	0.16	0.39	0.41	0.41	0.17	-0.30	-0.24
**RXN**	0.67	0.68	0.69	0.52	0.61	0.62	0.61	0.74	-0.16	0.13
**RXP**	0.83	0.85	0.86	0.19	0.72	0.74	0.74	0.40	-0.67	-0.34
**RXQ**	0.81	0.82	0.83	0.30	0.78	0.80	0.81	0.29	-0.53	-0.53
**RXR**	0.97	0.96	0.97	0.88	0.82	0.84	0.84	0.69	-0.09	-0.15
**RXW**	0.75	0.77	0.78	0.18	0.76	0.79	0.80	0.46	-0.60	-0.34
**RYJ**	1.43	1.44	1.46	0.47	2.38	2.32	2.33	1.69	-0.99	-0.65
**RYQ**	1.50	1.53	1.55	0.48	1.61	1.67	1.68	0.80	-1.07	-0.88
**RYR**	0.79	0.80	0.80	0.47	0.81	0.80	0.80	0.69	-0.33	-0.11
**XXX**	0.00	0.00	0.00	0.00	0.00	0.00	0.00	0.00	0.00	0.00
**Total**	6,468,586	6,268,013	6,138,487	129,526	208,326	196,769	193,270	3,499		
**χ^2 test**	$p < .001$ ($\chi^2 = 6267610.79$, df = 151)				$p < .001$ ($\chi^2 = 9154.95$, df = 151)					
