# Supplementary material for: Linkage of Maternity Hospital Episode Statistics data to birth registration and notification records for births in England 2005–2014: Quality assurance of linkage of routine data for singleton and multiple births
Source: BMJ Open. 2018 Mar 1;8(3):e017898. doi: 10.1136/bmjopen-2017-017898 (PMC5855305; doi:10.1136/bmjopen-2017-017898)
Supplement: Author's manuscript [file bmjopen-2017-017898.draft_revisions.pdf]

BMJ Open

BMJ Open is committed to open peer review. As part of this commitment we make the peer review history of every article we publish publicly available.

When an article is published we post the peer reviewers' comments and the authors' responses online. We also post the versions of the paper that were used during peer review. These are the versions that the peer review comments apply to.

The versions of the paper that follow are the versions that were submitted during the peer review process. They are not the versions of record or the final published versions. They should not be cited or distributed as the published version of this manuscript.

BMJ Open is an open access journal and the full, final, typeset and author-corrected version of record of the manuscript is available on our site with no access controls, subscription charges or pay-per-view fees (http://bmjopen.bmj.com).

If you have any questions on BMJ Open's open peer review process please email
editorial.bmjopen@bmj.com

Quality Assuring Linked ONS Birth Records and Maternity Hospital Episode Statistics Delivery Records for Singleton and Multiple Births in England, 2005 to 2014

Journal:	*BMJ Open*
Manuscript ID	bmjopen-2017-017898
Article Type:	Research
Date Submitted by the Author:	30-May-2017
Complete List of Authors:	Harper, Gillian; City, University of London, School of Health Sciences
**Primary Subject Heading**:	Research methods
Secondary Subject Heading:	Health services research
Keywords:	Data linkage, Quality assurance, Births and maternities, Administrative data

SCHOLARONE™ Manuscripts

For peer review only

Quality Assuring Linked ONS Birth Records and Maternity Hospital Episode Statistics Delivery Records for Singleton and Multiple Births in England, 2005 to 2014

Author: Gill Harper, Research Fellow, City, University of London, London, United Kingdom

Correspondence address

City, University of London,
Centre for Maternal and Child Health Research,
1 Myddelton Street,
London. EC1R 1UW.
Tel: 0207 040 5315
Email: gill.harper@city.ac.uk

ORCID Number: 0000-0002-3492-2076

For peer review only

Disclaimer

It is essential that any copyright agreement I sign is in the form of a non-exclusive agreement as, under the terms of my current contract, the Crown has been assigned an irrevocable non-exclusive licence in respect of any information, Intellectual Property, Results, Materials and conclusions arising from the research project.

Funding

This work was supported by the National Institute for Health Research. HS&DR Programme, project number HS&DR 12/136/93. Project title: 'Births and their outcome: analysing the daily, weekly and yearly cycle and their implications for the NHS'

Word count: 6,830

Number of tables: 5

Competing interests: None

Author contribution

The author Gill Harper was responsible for the design of the methodology, writing all parts of the paper, and giving final approval of the version to be published. I agree to be accountable for all aspects of the work in ensuring that questions related to the accuracy or integrity of any part of the work are appropriately investigated and resolved.

Data sharing statement

The author does not have permission to supply data or identifiable information to third parties, including other researchers but the research team of which she is a member has permission under Section 251 of the Health Service (Control of Patient Information) Regulations 2002 to analyse patient identifiable data for England and Wales without consent and create a research database which could be accessed by other researchers using the VML at the Office for National Statistics subject to permission from the Confidentiality Advisory Group of the Health Research Authority to access patient identifiable data without consent.

Keywords

Data linkage, Quality assurance, Administrative data, Births and maternities

Abstract

Objectives

To quality assure a Trusted Third Party linked dataset to prepare it for analysis.

Setting

Birth registration and notification records from the Office for National Statistics (ONS) for all **births** in England 2005-2014 linked to Maternity Hospital Episode Statistics (HES) delivery records by NHS Digital using mothers' identifiers.

Participants

All 6,676,912 births that occurred in England 1st January 2005 to 31st December 2014.

Primary and secondary outcome measures

Every link between a registered birth and a HES delivery record for the study period was categorised as either the same birth or a different birth to the same mother, or as a no-match, by comparing common baby data items and valid values in key fields with step-wise deterministic rules. Rates of preserved and discarded links were calculated and which features were more common in each group were assessed.

Results

98% of births originally linked to HES were left with one preserved link. The majority of discarded links were due to duplicate HES delivery records. Of the 4,854 discarded links categorised as no-match, clerical checks found 85% were false positives links, 13% were quality assurance false negatives, and 2% were undeterminable.

Births linked using a less reliable stage of the linkage algorithm, births at home and in the London region, and with birth weight or gestational age values missing in HES were more likely to have all links discarded.

Conclusions

Linkage error, data quality issues, and false negatives in the quality assurance procedure were uncovered. The procedure could be improved by allowing for transposition in date fields, and more discrimination between missing and differing values. The availability of identifiers in the datasets supported clerical checking. Other research using Trusted Third Party linkage should not assume the linked dataset is error-free or optimised for their analysis, and allow sufficient resources for this.

For peer review only

Strengths and Limitations

1. The study is the first time linked birth registration and hospital delivery records for ten years of data at the national level has been quality assured
2. A high linkage rate was maintained after quality assurance
3. The methodology was able to draw upon the availability of identifiers in both the ONS and HES data to inform the choice of rules at each stage in the methodology, to carry out clerical checks to ensure the accuracy of the results, and to explore data and linkage quality issues
4. Carrying the work out in the secure environment of ONS' VML safeguarded the data but limited software options and increased processing time
5. Any bias arising from non-submission of HES records by some trusts is likely to lead to bias in the extent to which records could be quality assured

For peer review only

Introduction

The quality assurance procedure described here was developed to support a project[1] for which data recorded at birth registration, birth notification and clinical information on maternity care at delivery were linked for analysis. After piloting in an earlier project,[2] the Office for National Statistics (ONS) mainstreamed the linkage of data recorded at birth registration in England and Wales to data recorded at birth notification, originally in an interim system, NHS Numbers for Babies (NN4B). These linked records of births from 2005 to 2014 were then linked to records from the Maternity Hospital Episode Statistics (HES) dataset for births that occurred in England and from the National Community Child Health Database /Patient Episode Database Wales (NCCHD / PEDW) for births that occurred in Wales. This linkage brought together key demographic and clinical data items unique to each dataset and not otherwise available together at a national level, enabling new analyses. For example, it made it possible to compare time of birth, recorded in birth notifications with birth outcomes such as mortality and conditions reported at birth, as ONS routinely links child death registration to birth registration records and reports. The further linkage with Maternity HES made it possible to analyses the outcomes of birth by onset of labour, mode of delivery, gestational age, time of day and day of the week. The history of the project and details of the linkage are described elsewhere[3].

The routinely linked ONS birth registration and birth notification records from here on will be referred to collectively as ONS birth records.

This paper describes quality assurance of the linked data for England, as data for Wales were linked separately.

Quality assuring this linkage aims to achieve two goals simultaneously. The first is to ensure that the ONS birth record has linked to the correct HES delivery record. As HES can contain more than one episode record for a delivery, the second aim of quality assurance is to select the most relevant HES delivery episode record with the most information from all the available correctly linked records.

Data sources

The ONS birth records were for 6,468,586 singleton and 208,326 multiple births in England from 1st January 2005 to 31st December 2014.

Only the Maternity HES delivery record which contains information on onset of labour and method of delivery was used. Maternity HES also contains a birth record for the baby, but linkage to these data and the further linkage to subsequent admissions of mothers and babies is not considered in this paper.

HES delivery records are usually grouped by financial year and were provided for the financial years 1st April 2004 to 31st March 2015 in order to include the calendar years used by ONS for its publications and analyses. They are for finished consultant episodes (FCEs) which capture information about diagnoses and procedures during the time a patient spends under the care of an individual consultant, or in the case of maternity, a midwife. Each delivery record contains an admitted patient care record with additional fields about the mother in an appended maternity tail, and information on the baby in an appended baby tail for up to nine babies.

Singleton and multiple birth deliveries are not explicitly identified in HES, but multiple delivery records can be identified where the diagnostic codes and the number of babies indicate a multiple

For peer review only - http://bmjopen.bmj.com/site/about/guidelines.xhtml

birth, and vice versa for singletons. On this basis, 7,040,590 HES delivery records relating to singleton births, and 123,497 relating to multiple births were identified. As stated above, the singleton and multiple births were quality assured separately. There can be more than one episode record per delivery, and some records are duplicated, therefore the number of HES delivery records is higher than the number of births.

Full details of the ONS and HES data are given elsewhere[3, 4] along with assessments of their data quality. [5-7] HES, particularly Maternity HES, has well recognised data quality problems[8] such as large numbers of missing data for some variables and missing maternity tails. The data recorded at birth registration is overseen by the General Register Office and processed by ONS for statistical purposes. ONS official statistics are considered as a 'gold' standard. Data quality has been found to influence the accuracy of linkage and create bias[2, 5-7, 9, 10]. Methods of cleaning HES data in isolation and by trust are discussed elsewhere.[11-14]

Linkage

Linkage of the ONS birth records to the Maternity HES delivery records was carried out by the Health and Social Care Information Centre, now known as NHS Digital, using maternal identifiers because HES delivery records are associated with mothers rather than their babies. It consists of a hierarchical step-wise linkage based on combinations of NHS Number, date of birth, sex, and postcode of the mother's place of residence (see Box 1). Every link is assigned the step of the algorithm that made the link, known as the match rank. On this basis, registered births to a mother during the study period 2005 to 2014 should link to all available delivery records for every birth that mother has had in those ten years.

Step/match rank	NHS number	DoB	Sex	Postcode	
**1**	Exact	Exact	Exact	Exact	
**2**	Exact	Exact	Exact		
**3**	Exact	Partial	Exact	Exact	
**4**	Exact	Partial	Exact		
**5**	Exact			Exact	
**6**		Exact	Exact	Exact	where NHSNO does not contradict the match and DOB is not 1 January and the POSTCODE is not in the 'ignore' list
**7**		Exact	Exact	Exact	where NHSNO does not contradict the match and DOB is not 1 January
**8**	Exact				

Box 1: NHS Digital hierarchical step-wise linkage algorithm used to link ONS birth records to HES delivery records

Mothers' identifiers from ONS birth records were linked first of all to the HES Patient Index to assign a HESID to each, then the HES delivery records associated with these HESIDs were extracted. Table 1 gives a summary of the linkage rates by calendar year, showing that 97% of singleton births and 95% of multiple births were linked to at least one HES delivery record.

For peer review only - http://bmjopen.bmj.com/site/about/guidelines.xhtml

Table 1: Summary of linkage rates for singleton births by calendar year

Instances where the linkage algorithm did not link a registered birth to a HES delivery record could be because no delivery record existed, for example for home births and births in private health facilities which are not usually captured by HES, non-submission of HES data by particular trusts at particular times, or due to linkage error. Overall, 1.7% of singleton and 4.3% of multiple ONS birth records without a link to a HES delivery record did have a link to the mother's HESID. These problems have not been investigated further in this paper.

This paper focuses only on validating the records that were linked to a mother's HESID and HES delivery record by NHS Digital's algorithm. Singleton and multiple births were assessed separately due to the extra complexity associated with multiple birth registration records. This paper sets out the quality assurance procedures and results for both.

Methods

The aim was to assess the linked ONS birth and HES delivery records at the national level to efficiently identify one correctly linked HES record with the maximum amount of delivery information from all possible links to create a file to be used in analysis.

Both mothers' and babies' identifiers were available for the ONS birth and HES delivery records to use in the quality assurance process. These were mother's date of birth, NHS number and postcode of usual residence, and baby date of birth, NHS number and sex.

The work was carried out in the secure environment of ONS' Virtual Microdata Laboratory (VML). Microsoft Access database software was used as it was the only database software available in the VML at that time, and Structured Query Language (SQL) code was used for replicability. The relatively large size of the data files and restrictions on database size and processing speed constrained the design of the methods used.

Data preparation

It was necessary to normalise the baby information in the maternity tail section of each HES delivery record so that the data could be stored and interrogated efficiently in the database environment. This meant that if information was held on more than one baby in the maternity tail part of a delivery record, this was transformed into separate records for each baby. For example, this meant two records, one for each of the two babies in a twin delivery. Each record contained the same common episode identifier, admitted patient care fields, and maternity tail fields, but with the respective baby's information in each one.

For variables common to birth registration and birth notification, the maximum amount of reliable information was derived from the two sources. The baby's date of birth, birthweight and sex were taken from birth registration, and gestational age was taken from birth notification, unless values were missing.

Data types and formats, for example date fields and values for the baby's sex were made consistent between ONS and HES records.

Data quality

The extent of missing values for key items of data about the baby in the ONS birth records for 1st January 2005 to 31st December 2014 and HES normalised records for 1st April 2004 to 31st March

For peer review only - http://bmjopen.bmj.com/site/about/guidelines.xhtml

2015 is given in Table 2. A value would have to be missing on both birth registration and birth notification to be considered missing overall on ONS birth records in Table 2. While the total numbers may not be directly comparable due to definitional and date differences, the percentages of missing values per variable gives an indication of differences in data completeness.

Table 2: Comparison of percentages of missing values in key baby data items on ONS birth records and HES normalised delivery records for singleton and multiple births 2005 to 2014

The HES data have a much higher proportion of records with missing values than ONS birth records, for example up to 37% for gestational age in singleton births. The proportion of records with missing information is lower for multiple births in HES, as data items for the first baby are often repeated for their siblings in the same delivery.

Where the baby's date of birth was missing on HES, it was derived from operation dates if a delivery code was present. If sex of baby, gestational age or birthweight were missing, they could not be derived, however.

Gestational age and birthweight are known to have problems, notably implausible values, truncation and rounding in HES. ONS birth records can also have implausible values for birthweight but this has been improving with the introduction of 'warning' edits to birth notification systems.

Procedure

There are few examples of existing procedures to quality assure linked datasets. One example[15] cleaned and validated linked HES and mortality data for England by re-formatting and deriving variables, removing duplicates, and breaking erroneous links indicated where common data items were highly discordant.

A pilot was initially carried out on data for 2005 to help formulate the methodology. The following procedure is the final version that was carried out on all ten years of the linked data. Clerical checks were carried out throughout the development phase using relevant variables and identifiers to inform and improve the procedure.

On the assumption that the maternal linkage was correct, the links between the ONS birth records and HES delivery records were categorised as either 'same mother same birth' (SMSB) or 'same mother different birth' (SMDB). The category 'wrong birth' (WB) was for where neither of the previous categories apply, suggesting incorrect maternal linkage or data quality problems. These scenarios are summarised in Box 2. This excludes occasions when a birth registration record did not link to any HES delivery records.

An ONS birth record may link to one correct or incorrect HES delivery record, or to more than one HES delivery record with one, none or some of them being correct for any of the reasons in Box 2.

Category	Summary	Details	Action
Same mother same birth (SMSB)	ONS birth record linking to one relevant HES delivery record	One-to-one linkage as expected	Keep link
Same mother same birth (SMSB)	ONS birth record linking to many relevant HES delivery records, no clear episode order sequence	Due to duplicate HES records, or pre- and post-delivery HES records	Keep link to one of the duplicates or the most appropriate delivery record<br>Move others to unlinked file
Same mother same birth (SMSB)	ONS birth record linking to many relevant HES records, with a clear episode order sequence	Due to multiple episodes as part of a hospital spell	Keep link to one episode record and maximise delivery information

			Move others to unlinked file
Same mother different birth (SMDB)	ONS birth record linking to one or more incorrect HES delivery record (same mother)	Another birth to the same mother within the study period	Move to unlinked file
Wrong birth (WB)	ONS birth record linking to one or more incorrect HES delivery record	The maternal linkage is incorrect or poor data quality	Move to unlinked file

Box 2: Categories of linked ONS birth records and HES delivery records

The quality assurance exercise aimed to keep 'same mother same birth' linked records (with the correct normalised record for multiple births), and move all others to the unlinked file. If more than one correct link was available, only one linked delivery record containing the maximum delivery information was chosen. This was to create a one-to-one relationship and support ease of analysis.

The complexity of quality assurance was greater for multiple births because each ONS birth record linked to all HES normalised records for that delivery through the mother's identifiers, by corresponding to each of the siblings.

Baby data items were compared to ascertain if the linked records related to the same baby. These were baby's date of birth (derived date of birth in HES where applicable), sex, gestational age, birthweight, and location of birth.

The place of birth codes used differed between the datasets. These were translated into a consistent common location code, designed specifically for the study.[16]**Error! Bookmark not defined.** This accounted for NHS organisational changes which took place over the time period. Many maternity services continued to operate in the same location, while the NHS trusts responsible for them and, consequently their organisation code, changed over the study period. In addition, groups of location codes were identified that were within a reasonable geographical distance of each other that might transfer women between them, for example, if they developed problems which made it advisable to transfer to a hospital with a specialist neonatal unit. The presence of codes within these groups on the linked data could relate to the same birth.

Locations of births out-with an NHS trust, such as home births, were all treated as potential location matches because they could ultimately be transferred to an NHS hospital and have a HES delivery record, and the recording of the location for such births in HES can be unreliable. For these cases the other baby data items were relied on to establish if records were for the same baby.

Four combinations of these variables were used to confirm whether or not a linked ONS birth record and HES delivery record related to the same baby (see Box 3), in order of decreasing certainty from 1 to 4. Combination 1 required all the data items to match. Combination 2 required location and date of birth to match plus either birthweight or gestational age or sex. Combination 3 required only location and date of birth to match and birthweight, gestational age and sex will not match in these cases. Combinations 2 and 3 took into account the high occurrence of missing values and poor data quality in HES. Combination 4 accounted for a degree of data entry error in the date of birth by allowing it to differ by up to 4 days, but only if backed up by the location of birth matching, and birthweight and gestational age and sex not differing very much between ONS and HES if the values were not missing. If they were different and the date of birth was not an exact match, it was considered to be a different baby. The four combinations were applied in order so the most reliable matches are identified first.

For peer review only - http://bmjopen.bmj.com/site/about/guidelines.xhtml

Combination	ONS birth record and HES location of birth matches		ONS birth record and HES baby date of birth matches		ONS birth record and HES birthweight matches		ONS birth record and HES gestational age matches		ONS birth record and HES sex of baby matches
1	**Y**	**and**	**Y**	**and**	**Y**	**and**	**Y**	**and**	**Y**
2	**Y**	**and**	**Y**	**and**	**(Y**	**or**	**Y**	**or**	**Y)**
3	**Y**	**and**	**Y**						
4	**Y**	**and**	**Differs by up to 4 days***	**and**	**Missing or not completely different**	**and**	**Missing or not completely different**	**and**	**Missing or not completely different**

*by up to 10 days for multiple births

Box 3: The four combinations of baby data items that confirm that the link between the ONS birth record and HES relates to the same baby

For peer review only

If the linked records referred to the same baby and there was more than one linked HES delivery record to choose from, then the record with the maximum onset of delivery and delivery method information was chosen, as these variables were of key importance for the analysis. The presence of valid values in these and other fields were used to inform this.

The procedure was implemented using deterministic step-wise rules to be carried out in order, with any linked records dealt with at each stage excluded from subsequent stages. A summary is given in Box 4. Fuller details are given in Appendix A. It is slightly different for singleton and multiple births to account for the increased complexity in the multiple births linkage.

**Stage 1 - Cleaning**
Exclude links to invalid HES delivery records<br>Exclude links to surplus x 6 or x 9 duplicate HES delivery records
**Stage 2 - Same mother different birth**
Exclude links to HES delivery records where the HES baby date of birth is too far away from the ONS baby date of birth to be the same birth
**Stage 3 – Only one HES delivery record linked to an ONS birth record**
Keep if baby data items match<br>Exclude if baby data items do not match
**Stage 4 – Multiple HES delivery records all in same spell linked to an ONS birth record**
Keep episode if baby data items match and contains maximum delivery information<br>Exclude episode(s) if baby data items do not match and/or episode contains less delivery information
**Stage 5 – Multiple HES delivery records containing duplicate information linked to an ONS birth record**
Keep record if baby data items match with latest episode dates<br>Exclude record(s) if baby data items do not match and/or earlier episode dates
**Stage 6 - Multiple HES delivery records containing different information linked to an ONS birth record**
Keep record if baby data items match and contains maximum delivery information<br>Exclude record(s) if baby data items do not match and/or contains less delivery information

Box 4: Summary of quality assurance procedure

The first two rules cleaned the HES data by eliminating invalid linked records and duplicates. It is known that some trusts generate multiple copies of the same delivery episode record, usually six or nine copies of the same record. In these cases only one was kept to be quality assured and the others were discarded for efficiency.

Checks

Once all rules in the procedure had been carried out, it was confirmed that all linkages had been assigned a decision, and there were no more records to check.

Further checks were made on:

1. Private hospital births should not have a link to a HES delivery record unless the mother or baby were later transferred to an NHS hospital for any reason
2. Each HES delivery record should link to the correct number of ONS birth records depending if it is for a singleton or multiple delivery
3. The ONS birth record baby date of birth fell within the HES delivery admission period (no admission dates for home births). This was not always the case even when it was a confident link
4. If there was more than one correct and relevant linked HES delivery record for an ONS birth record, any missing onset of delivery and method of delivery information was filled in from these

Results

The quality assurance procedure took the equivalent of 55 working days for singleton births and 16 days for multiple births. The database software and processing limitations of the VML at that time slowed the procedure down. SQL Server has since been made available in VML and would speed this up considerably.

Seventeen per cent of singleton births linked records were dealt with in the first two cleaning stages, and a further 80% were dealt with by rules 3 and 4. All the remaining rules were required to deal with the residual 3% of more complex cases.

Five per cent of multiple births linked records were dealt with in the first two cleaning stages, and a further 60% were dealt with by rules 3 and 4. This left 35% as residual records, reflecting the greater complexity in multiple birth records.

For each year, the complex residual cases were clustered within particular trusts. This was probably due to IT problems and changes to systems.

A summary of the results for singleton and multiple births is given in Tables 3a) and 3b), broken down by calendar year.

Table 3a: Summary of quality assurance results for singleton births by calendar year (QA = quality assurance)
Table 3b: Summary of quality assurance results for multiple births by calendar year (QA = quality assurance)

Of the total 6,468,586 singleton ONS birth records, 97% had been linked to one or more HES delivery records and could be quality assured. Of these, 98% remained linked to one HES delivery record and 2% had all HES links discarded after quality assurance. Ninety five per cent of all ONS singleton birth records overall were left with a link to a HES record for analysis.

Of the 208,326 multiple births, 95% were linked to one or more HES delivery records and could be quality assured. Of these, 98% remained linked to one HES delivery record and 2% had all HES links discarded after quality assurance. Ninety three per cent of all ONS multiple birth records overall were left with a link to a HES record for analysis.

For peer review only - http://bmjopen.bmj.com/site/about/guidelines.xhtml

For singleton births, the percentage of all birth records left linked to one HES delivery record after quality assurance increased more or less year on year from 93% in 2005 to 95% in 2014. For multiple births, the rate increased from 94% in 2005 to 97% on 2010, but then started to decrease again to 87% in 2014. This reflects the same patterns as the percentage of ONS birth records that were originally linked to a HES delivery record and are biased by the number of records that linked to a HESID but no delivery record that were excluded from quality assurance.

Reasons for discarding HES records linked to an ONS birth

An ONS birth record may have been linked to one or more HES records. After quality assurance, all or some of the available linked HES records will have been discarded, leaving only a link to one correct HES record, or to none at all. Discarding all the linked HES records for a birth suggests incorrect linkage and is discussed in the next section. Discarding all but one is due to a variety of reasons why there may be more than one HES record available for a birth.

Seventy six per cent of all the linked HES records that were discarded were due to duplicate copies of HES delivery episodes, and 0.5% were due to invalid delivery records, dealt with in the cleaning stages 1 and 2 of the methodology. A summary of the remaining reasons by year for singleton births is given in Table 4.

Table 4: Breakdown of reasons for discarding HES delivery records linked to an ONS birth excluding duplicate copies of HES delivery episodes and invalid deliveries for England singleton births by year

'Same mother different births' (column a) accounted for 60% of all discarded links. This is expected when the study period covers ten years and each birth is linked by the mothers' identifiers to all other deliveries to that mother in that period. The second main reason which accounted for 21% of discarded links, was when there were additional episodes relating to a delivery, usually a pre- or post-delivery admission (column b). The numbers of these generally decreased over time, suggesting data quality is a factor. The third main reason, accounting for 16%, was when links to other episodes in a spell have been discarded, keeping only the link to the episode with the most amount of delivery information (column c).

The remaining three reasons each accounted for fewer than 2% of discarded links. Column d is when multiple delivery records contained the same information and links to superfluous records were discarded. This is also a data quality issue. There was a large increase in these in 2014. Column f relates to a small number of linked singleton birth records that were identified as 'potential multiple births', described later in this paper.

Column e are 'no matches'; links discarded where there are no matching variables or insufficient numbers of matching variables according to the quality assurance methodology, and the baby's date of birth did not indicate a 'same mother different birth'. This group is of interest because it could be indicating erroneous false positive links by the linkage algorithm, or it could indicate erroneous false negatives in the quality assurance methodology.

Fifty seven per cent (2,743) of this group were linked by match rank 1 of the linkage algorithm, the most reliable stage of the linkage algorithm that matches all available identifiers including the NHS number, suggesting they should be correct maternal linkages. These were checked in greater detail clerically for explanations.

Of these, 2,323 (85%) of these were true no matches where either none of the five baby data items matched at all, or only the place of birth matched, or only sex matched, or only gestation matched.

The baby's date of birth has not been transposed nor were 'same mother different birth', suggesting incorrect maternal linkage or some other data quality issue. This is 0.04% of all singleton birth records originally linked to a HES delivery record.

It appeared that 366 (13%) were potential false negatives made in error by the quality assurance methodology. The dates of birth in these do not match due to transposition in the date of birth elements. If this had been factored into the methodology, these links would have not been discarded.

The remaining 94 (3%) were undeterminable, with some discriminatory baby data items matching and some not. For example, the date of birth may match or have been transposed, but birthweight and/or place of birth may be very different.

Some of the categories of problem shown in Table 4 may have been restricted to particular trusts but further investigation would be needed to check this.

Differences between records with correct and incorrect linkage

Checks were carried out to ascertain if the ONS birth records that remained correctly linked to one HES delivery record after the quality assurance procedure, and the ONS birth records with all links to HES discarded after the quality assurance procedure differed significantly by key variables. Discarding all links could indicate linkage error, data quality issues, or false negatives in the quality assurance methodology.

Appendix B gives tables to compare the proportions of values for the key variables for both groups, and chi-square tests results for testing the null hypothesis that the distribution of a variable is the same in the two groups. Variables from the ONS birth record include the match rank of the linkage algorithm, the region of birth, the year of birth, the month of birth, the day of birth (8) based on an 8 day categorisation including public holidays, the day of birth (11) based on an 11 day categorisation including specific public holidays and the days before and after them, the hour of birth, the age of the mother, the sex of the baby, the ethnicity of the baby, gestational age, if a stillbirth, and the location of birth. Missing gestational age and missing birthweight in HES were also included.

The null hypothesis is rejected for all variables except for sex, day (8), and day (11) in multiple births. Finding significant differences is to be expected because the dataset is large and the chi-square test can pick up subtle differences, but this does not tell us very much. Comparing the difference in the percentage distribution of values for each variable for the two groups provides more information on how they differ. This is given in Table 5 for singleton and multiple births where the difference is greater than 1%.

Table 5: Comparing the difference in the percentage distribution of values for key variables for ONS birth records that remained correctly linked to one HES delivery record after the quality assurance procedure, and the ONS birth records with all links to HES discarded after the quality assurance, for singleton and multiple births

These differences give an indication of when incorrect linkage is more likely to occur, or indeed false negatives in the quality assurance. Differences between singleton and multiple births are seen. Some of these associations with incorrect linkage make sense. Home births are less likely to have a HES delivery record as many are not captured by hospital-based clinical systems from which HES records are derived. Match rank 6 is a less reliable stage of the linkage algorithm because it does not match on the NHS number. HES data quality is previously known to be poorer in years 2006 and 2007, although for multiple births, years 2012 to 2014 are more likely to be incorrectly linked. Missing data

For peer review only - http://bmjopen.bmj.com/site/about/guidelines.xhtml

for gestational age and birthweight in HES values means there is less information to make a correct link. The other factors require more investigation or could be simply due to chance with the large numbers involved.

Table B.1 in Appendix B breaks down the proportions of each group by the match rank of the NHS Digital linkage algorithm, providing useful information about the quality of their linkage at each stage.

Data and linkage quality issues

Quality assurance of the linked dataset for such a large population revealed issues with the quality of the data within each of the datasets and also the quality of linkage that otherwise might not be detectable. While it is already known that HES has data quality issues, issues were found in the ONS birth records that warranted further investigation.

Firstly, the consistent common location code that was assigned to the place of birth codes was found to not match between birth registration and notification for 22% of the singleton births with a correct HES link after quality assurance. This suggested the linkage between birth registration and notification could be incorrect. A sample of these records that also had very discordant mother and baby identifiers and baby tail information was given to ONS, which carried out this linkage, for checking.

ONS confirmed that the baby NHS number or the sequence number[17] matched for these cases and therefore the linkage was correct between ONS birth registration and birth notification, regardless of the discordant variables. However, no quality assessment of the sequence number assignment process was available, so 542 of the most suspect cases were flagged. It was decided to exclude these from analyses.

Secondly, a small number of singleton birth records appeared to be multiple birth records (see Table 4 column f) based on a combination of HES variables suggesting a multiple birth delivery, and other birth registrations existing to the same mother on the same day, yet these were not labelled as a multiple birth by ONS. Again, these cases were checked by ONS which confirmed that they should be multiple births. The error was explained by a delay in some records being registered and received, possibly causing issues with finding the corresponding record in its system.

Lastly, after quality assurance, a small number of multiple births did not have all the expected number of ONS birth records, for example, a twin birth only had one ONS birth record associated with it rather than two. These were not always explained by stillbirths prior to 24 weeks gestation which should not be registered as a birth. Investigation revealed that the missing ONS birth records could often be found in the 'unlinked' ONS birth records file, to which no HES records were linked. This could be the case even if the mothers' identifiers, including NHS number, clearly matched.

This was discussed with our contact at NHS Digital who investigated this. It was explained that these missed links were due either to the HES patient index having been updated since the linkage was done originally, or linkage error. The project did not attempt to find any further missing links.

The advantage of having the identifiers available to the project team for both ONS birth and HES delivery records made it possible to investigate and explain the above issues, but other research projects may not. Overall, this highlights how researchers and analysts should not assume that any routinely collected administrative datasets and linkages carried out between them are error-free.

For peer review only - http://bmjopen.bmj.com/site/about/guidelines.xhtml

Discussion

Main findings

This paper describes the quality assurance process that was carried out on ten years of linked ONS birth records and Maternity HES delivery records at a national level using patient identifiers. The aim was to prepare the linked dataset for analysis but the results also shed light on the quality of the data and the linkage itself. This makes it informative for other users of similarly linked data and for NHS Digital staff doing the linkage.

The method adopted compares common baby data items, giving most importance to matching babies' date of birth and location of birth. Altogether, 95% of all singleton births and 93% of all multiple births in England in the period 2005 to 2014 were linked to one correct HES delivery record after quality assurance.

Administrative duplicate HES records were found to be the main reason for discarding links. Other reasons were due to other births to the same mother within the study period, the presence of multiple HES records per delivery for various reasons, and error in the linkage and in the quality assurance method itself.

Certain features were more common in incorrectly linked records than correctly linked records. These included births linked using match rank 6 of the linkage algorithm, births at home and in the London region, and births where the birthweight or gestational age was missing in HES. This suggests some bias in the linkage and often relates to data quality issues. These patterns differed between singleton and multiple births.

Missed links were also discovered, which together with the previous findings provides further evidence on the quality of the linkage.

It was possible to identify aspects of the quality of the ONS birth records data and have ONS investigate them.

Strengths and limitations, and future work

The quality assurance procedure was able to draw upon the availability of identifiers in both the ONS and HES data to inform the choice of rules at each stage in the methodology, to carry out clerical checks to ensure the accuracy of the results, and to explore data and linkage quality issues. The procedure successfully identified correct and incorrect linkages to prepare the large linked dataset for analysis within the resources available.

Because personally identifiable data was used, the work was carried out in the secure environment of ONS' VML. While security was safeguarded, this led to many delays and disruption due to software and processing limitations of systems and servers. This was costly in terms of researcher time and has limited the extent of analytical outputs from the study.

Quality assurance could only be carried out on ONS birth records that had linked to a HES delivery record. Links where a HESID was found but no delivery record was available were excluded. If the main reason for this is because some trusts did not submit the HES delivery record, then bias will be inherently present in which linked records could be quality assured and which couldn't.

The methodology to decide if the linked records related to the same baby was purely deterministic and did not allow for transposition of date of birth elements, or for rounding and truncating in the

For peer review only - http://bmjopen.bmj.com/site/about/guidelines.xhtml

birthweight and gestational age values. It did not strongly discriminate between when values were missing rather than different.

A disproportionate amount of effort was spent on the approximately 4% of complex linked records that remained after stages 1-4 of the methodology.

Future work could address these issues by incorporating more flexible matching criteria and more thoroughly preparing the birthweight and gestational age variables for data entry errors beforehand. Ways to simplify the methodology to fewer variable combinations or rules to identify correct and incorrect links could be explored. Future work could also assess how variation in data quality at trust level affects linkage quality and the results.

Implications

The linkage error identified by quality assuring the linked dataset suggests that linkage carried out using similar algorithms and by 'Trusted Third Parties' generally, cannot be assumed to be error-free and may affect any analyses carried out on them. This has implications for other research projects that are not informed of the quality of the linkage, or have the means to quality assure it themselves using identifiers. In particular, no assessment of the quality of the linkage algorithm is routinely published by NHS Digital.

A very similar linkage algorithm is used to routinely link HES data to ONS death registration data, differing only at match rank 8,[18] although different approaches were used earlier by the National Centre for Health Outcomes Development[19] and the Department of Health. This linked data is used to analyse outcomes of hospital care such as immediate and long-term survival rates and hospital-based mortality indices including the Standard Hospital Mortality Index (SHMI) and indices of 30-day mortality. Concerns over how quality of this linkage can affect analyses using the 30-day mortality indicator have been reported.[20] On the other hand, an evaluation of the mortality linkage for SHMI purposes found that 'the proportion of erroneous linkages is sufficiently small to not impact on a SHMI result', but its report did not explicitly describe the linkage algorithm used.[21] The relevant member of its study team has confirmed that the team did not know what linkage algorithm was used by the then Information Centre for Health and Social Care to link the mortality data for SHMI purposes, and they were not provided with NHS numbers or other identifiable variables to enable performing the linkage themselves or indeed to investigate the quality of the linkage (Fotheringham J, personal communication).

Linkage of identifiable data for research purposes is mostly carried out by 'Trusted Third Parties' such as NHS Digital. The Trusted Third Party model replaces identifiers with an encrypted code, and only the de-identified data are made available to researchers in a secure environment. The Digital Economy Bill[22] announced in May 2016, sought increased access to de-identified data for research, meaning that reliance on Trusted Third Party linkage would increase. The Bill implies that data accessed under the new legislation must only be linked using the Trusted Third Party model. However, responses to this and to The Cabinet Office consultation document 'Better Use of Data in Government'[23] from bodies such as the Royal Statistical Society[24] and the Health Statistics User Group[24] have expressed concern over this and also that the proposed legislation does not include health and social care data. Elements of the Digital Economy Bill could be interpreted as allowing flexibility in who can access and link identifiable data so long as disclosure is avoided and other conditions and codes of practice are in place. Clarification on this is required so that it can be established if researchers will be restricted only to pre-linked data without any means to assess their quality.

For peer review only - http://bmjopen.bmj.com/site/about/guidelines.xhtml

In the context of the use of linked data for statistics, the ONS has expressed a preference to be able to link data with identifiers rather than encryption to achieve better quality official population statistics in the future[25].

Conclusion

Carrying out quality assurance of linked records has informed knowledge of both the quality of the data and of the linkage, providing benefits over cleaning each dataset in isolation. Other research projects using large linked routinely collected datasets should not assume the linked datasets are error-free or optimised for their analysis. This has implications especially for users of anonymised linked data. The resources needed to do quality assurance and prepare effectively prior to generating descriptive or analytic reports are considerable and need to be addressed when planning and costing large data linkage projects.

For peer review only

Acknowledgement

The author would like to thank all the relevant colleagues in the Office for National Statistics and NHS Digital, formerly the Health and Social Care Information Centre for their help. In particular, to thank Emma Gordon, Joanne Evans, Claudia Wells, Alex Lloyd, Justine Pooley, Elizabeth Mclaren and members of the VML Team at the Office for National Statistics, and Ariane Alamdari and Garry Coleman at NHS Digital. I would also like to thank the members of the project team and its Study Advisory Group for their help and advice.

Disclaimers

The views and opinions expressed here are those of the author and do not necessarily reflect those of the HS&DR programme, NIHR, NHS or the Department of Health.

The data were processed in the secure environment of the Office for National Statistics' Virtual Microdata Laboratory and the following disclaimer applies:

This work contains statistical data from ONS which is Crown Copyright. The use of the ONS statistical data in this work does not imply the endorsement of the ONS in relation to the interpretation or analysis of the statistical data. This work uses research datasets which may not exactly reproduce National Statistics aggregates.

Approvals

Ethics approval 05/Q0603/108 and subsequent substantial amendments were granted by East London and City Local Research Ethics Committee 1 and its successors.

Permission to use patient identifiable data without consent under Section 60 of the Health and Social Care Act 2001 was initially granted by the Patient Information Advisory Group PIAG 2-10(g)/2005. Renewals and amendments and a second permission CAG 9-08(b)2014 under Section 251 of the Health Service (Control of Patient Information) Regulations 2002 were granted by its successor bodies, the Ethics and Confidentiality Committee of the National Information Governance Board and the Confidentiality Advisory Group of the Health Research Authority.

A second permission CAG 9-08(b)2014 to use patient identifiable data without consent under Section 251 of the Health Service (Control of Patient Information) Regulations 2002 and create a research database held at the Office for National Statistics for analyses relating to inequalities in the outcome of pregnancy and to inform maternity service users about the outcome of midwifery, obstetric and neonatal care was granted by the Confidentiality Advisory Group of the Health Research Authority.

Permission to access data from the Office for National Statistics in the VML was granted by ONS' Microdata Release Panel. All members of the research team successfully applied for ONS Approved Researcher Status.

Permission to link and analyse data held by the Health and Social Care Information Centre, now NHS Digital was granted under Data Sharing Agreement NIC-273840-N0N0N.

Copyright

It is essential that any copyright agreement I sign is in the form of a non-exclusive agreement as, under the terms of my current contract, the Crown has been assigned an irrevocable non-exclusive licence in respect of any information, Intellectual Property, Results, Materials and conclusions arising from the research project.

For peer review only

References

1. 'Births and their outcome: analysing the daily, weekly and yearly cycle and their implications for the NHS' funded by the National Institute for Health Research. HS&DR Programme, project number HS&DR 12/136/93.
2. Hilder L, Moser K, Dattani N, Macfarlane A. Pilot linkage of NHS Numbers for Babies data with birth registrations. Health Statistics Quarterly. 2007(33):25-33.
3. Dattani N. Linkage of Maternity Hospital Episode Statistics data to birth registration and NHS Numbers for babies records, England 2005-2014: methods. Forthcoming.
4. Ghosh RE, Ashworth DC, Hansell AL, Garwood K, Elliott P, Toledano MB. Routinely collected English birth data sets: comparisons and recommendations for reproductive epidemiology. Arch Dis Child Fetal Neonatal Ed. 2016;101(5):F451-7.
5. Hockley C, Quigley M, Johnson J, Rosenberg R, Dezateux C, Joshi H. Millennium Cohort Study: Birth Registration and Hospital Episode Statistics Linkage. A Guide to the Dataset. Centre for Longitudinal Studies Bedford Group for Lifecourse & Statistical Studies Institute of Education, University of London, 2007.
6. Dattani N, Datta-Nemdharry P, Macfarlane A. Linking maternity data for England, 2005-06: methods and data quality. Health Stat Q. 2011(49):53-79.
7. Dattani N, Datta-Nemdharry P, Macfarlane AJ. Linking maternity data for England 2007: methods and data quality. Health Statistics Quarterly. 2012;53:4-21.
8. HES Data Quality: NHS Digital. Available from: http://content.digital.nhs.uk/article/1825/The-processing-cycle-and-HES-data-quality.
9. Hagger-Johnson G, Harron K, Fleming T, Gilbert R, Goldstein H, Landy R, et al. Data linkage errors in hospital administrative data when applying a pseudonymisation algorithm to paediatric intensive care records. BMJ Open. 2015;5.
10. Knight HE, Gurol-Urganci I, Mahmood TA, Templeton A, Richmond D, van der Meulen JH, et al. Evaluating maternity care using national administrative health datasets: how are statistics affected by the quality of data on method of delivery? BMC Health Serv Res. 2013;13:200.
11. Raleigh VS, Foot C. Getting the Measure of Quality: Opportunities and Challenges. London: 2010.
12. Sinha S, Peach G, Poloniecki JD, Thompson MM, Holt PJ. Studies using English administrative data (Hospital Episode Statistics) to assess health-care outcomes—systematic review and recommendations for reporting. Eur J Public Health. 2013;23(1):86-92.
13. Royal College of Obstetricians and Gynaecologists. Patterns of Maternity Care in English NHS Hospitals 2011/12. London: 2013.
14. Sandall J, Murrells T, Dodwell M, al e. The efficient use of the maternity workforce and the implications for safety and quality in maternity care: a population-based, cross-sectional study. Health Services and Delivery Research. 2014;2(38).
15. RCPCH Child Health Reviews – UK Clinical Outcome Review Programme. Overview of child deaths in the four UK countries. September 2013.
16. Dodwell M. Assigning pseudo trusts to birth locations. Forthcoming.
17. Pregnancy and ethnic factors influencing births and infant mortality: 2013: ONS. Available from: https://www.ons.gov.uk/peoplepopulationandcommunity/healthandsocialcare/causesofdeath/bulletins/pregnancyandethnicfactorsinfluencingbirthsandinfantmortality/2015-10-14#data-sources-and-linkage.
18. Guide to linked mortality data from HES and the Office for National Statistics: NHS Digital. Available from: http://content.digital.nhs.uk/media/11668/HES-ONS-Mortality-Data-Guide/pdf/mortality_guide.pdf.
19. Gill LE, Goldacre MJ. English National Record Linkage of Hospital Episode Statistics and Death Registration Records. Report to the Department of Health, 2003. National Centre for Health Outcomes Development, Unit of Health-Care Epidemiology, University of Oxford, 2003.

For peer review only

20. Cooper Z, Gibbons S, Jones S, McGuire A. Does hospital competition save lives? Evidence from the NHS patient choice reforms. Economic Journal. 2011;121:228-60.

21. Campbell MJ, Jacques RM, Fotheringham J, Pearson T, Maheswaran R, Nicholl J. An evaluation of the Summary Hospital Mortality Index. Final report. Sheffield: ScHARR, The University of Sheffield, 2011.

22. Digital Economy Bill: House of Commons. Available from: https://www.publications.parliament.uk/pa/bills/cbill/2016-2017/0045/cbill_2016-20170045_en_1.htm.

23. Better use of data in government: Cabinet Office. Available from: https://www.gov.uk/government/consultations/better-use-of-data-in-government.

24. Statement on the Cabinet Office consultation 'Better use of data in government': Royal Statistical Society; 1 April 2016. Available from: http://www.rss.org.uk/Images/PDF/influencing-change/2016/RSS-Statement-on-Better-Use-of-Data-consultation-April-2016.pdf.

25. Methodology of Statistical Population Dataset V2.0: ONS. Available from: https://www.ons.gov.uk/census/censustransformationprogramme/administrativedatacensusproject/methodology/methodologyofstatisticalpopulationdatasetv20#summary-and-future-developments.

For peer review only

Tables

Table 1a: Summary of linkage rates for singleton births by calendar year

Year	Total singleton ONS birth records	Total singleton ONS births linked to any Maternity HES by NHS Digital	Total singleton ONS births not linked to any Maternity HES by NHS Digital	Singleton ONS birth records linked to HESID but no delivery record	% Total singleton ONS births linked to any Maternity HES by NHS Digital
2005	599,237	565,559	33,678	16,349	94.4
2006	620,730	589,127	31,603	15,802	94.9
2007	638,995	612,782	26,213	12,889	95.9
2008	656,196	635,411	20,785	9,908	96.8
2009	653,322	636,284	17,038	9,099	97.4
2010	665,599	652,533	13,066	7,009	98.0
2011	666,582	653,552	13,030	8,329	98.0
2012	676,399	661,511	14,888	11,444	97.8
2013	647,666	633,222	14,444	11,913	97.8
2014	643,860	628,032	15,828	13,514	97.5
**Total**	**6,468,586**	**6,268,013**	**200,573**	**116,256**	**96.9**

Table 1b: Summary of linkage rates for multiple births by calendar year

Year	Total multiple ONS birth records	Total multiple ONS births linked to any Maternity HES by NHS Digital	Total multiple ONS births not linked to any Maternity HES by NHS Digital	Multiple ONS birth records linked to HESID but no delivery record	% Total multiple ONS births linked to any Maternity HES by NHS Digital
2005	18,376	17,404	972	510	94.7
2006	19,541	18,522	1,019	602	94.8
2007	20,066	19,257	809	450	96.0
2008	20,803	20,100	703	378	96.6
2009	22,008	21,338	670	369	97.0
2010	21,501	21,033	468	276	97.8
2011	22,099	21,199	900	722	95.9
2012	22,058	20,166	1,892	1,805	91.4
2013	20,767	18,735	2,032	1,917	90.2
2014	21,107	19,015	2,092	2,009	90.1
**Total**	**208,326**	**196,769**	**11,557**	**9,038**	**94.5**

Table 2: Comparison of %s of missing values in key baby data items on ONS birth records and HES normalised delivery records for singleton and multiple births 2005 to 2014

For peer review only

Variable	ONS singleton birth records	HES singleton delivery records	ONS multiple birth records	HES multiple delivery records
Total	6,468,586	7,040,590	208,326	230,019
% with missing values for:				
Baby's date of birth	0.00	19.4	0.00	16.3
Sex of baby	0.00	23.1	0.00	16.1
Gestational age	0.71	37.2	0.80	27.8
Birthweight	0.56	22.7	1.70	15.3

For peer review only

Table 3a: Summary of quality assurance results for singleton births by calendar year (QA = quality assurance)

Year	Total singleton ONS birth records	Total singleton ONS births linked to any Maternity HES by NHS Digital	% Total singleton ONS births linked to any Maternity HES by NHS Digital	Of singleton ONS births linked to any Maternity HES by NHS Digital, total left with one link to HES after QA	Of singleton ONS births linked to any Maternity HES by NHS Digital, % left with one link to HES after QA	Of singleton ONS births linked to any Maternity HES by NHS Digital, total left with no link to HES after QA	Of singleton ONS births linked to any Maternity HES by NHS Digital, % left with no link to HES after QA	% of total singleton ONS births left with one link to HES after QA	% of total singleton ONS births left with no link to HES after QA
2005	599,237	565,559	94.4	554,566	98.1	10,993	1.9	92.5	7.5
2006	620,730	589,127	94.9	573,770	97.4	15,357	2.6	92.4	7.6
2007	638,995	612,782	95.9	595,585	97.2	17,197	2.8	93.2	6.8
2008	656,196	635,411	96.8	621,006	97.7	14,405	2.3	94.6	5.4
2009	653,322	636,284	97.4	621,423	97.7	14,861	2.3	95.1	4.9
2010	665,599	652,533	98.0	641,167	98.3	11,366	1.7	96.3	3.7
2011	666,582	653,552	98.0	642,263	98.3	11,289	1.7	96.4	3.6
2012	676,399	661,511	97.8	648,501	98.0	13,010	2.0	95.9	4.1
2013	647,666	633,222	97.8	622,943	98.4	10,279	1.6	96.2	3.8
2014	643,860	628,032	97.5	617,263	98.3	10,769	1.7	95.9	4.1
Total	6,468,586	6,268,013	96.9	6,138,487	97.9	129,526	2.1	94.9	5.1

For peer review only

Table 3b: Summary of quality assurance results for multiple births by calendar year (QA = quality assurance)

Year	Number of multiple ONS birth records	Number of multiple ONS births linked to any Maternity HES by NHS Digital	Percentage of multiple ONS births linked to any Maternity HES by NHS Digital	Number left with one link to HES after QA	Percentage left with one link to HES after QA	Number left with no link to HES after QA	Percentage left with no link to HES after QA	Percentage of all multiple ONS births left with one link to HES after QA	Percentage of all multiple ONS births left with no link to HES after QA
2005	18,376	17,404	94.7	17,209	98.9	195	1.1	93.6	1.1
2006	19,541	18,522	94.8	18,258	98.6	264	1.4	93.4	1.4
2007	20,066	19,257	96.0	18,957	98.4	300	1.6	94.5	1.5
2008	20,803	20,100	96.6	19,894	99.0	206	1.0	95.6	1.0
2009	22,008	21,338	97.0	21,085	98.8	253	1.2	95.8	1.1
2010	21,501	21,033	97.8	20,847	99.1	186	0.9	97.0	0.9
2011	22,099	21,199	95.9	20,872	98.5	327	1.5	94.4	1.5
2012	22,058	20,166	91.4	19,554	97.0	612	3.0	88.6	2.8
2013	20,767	18,735	90.2	18,165	97.0	570	3.0	87.5	2.7
2014	21,107	19,015	90.1	18,429	96.9	586	3.1	87.3	2.8
**All years**	**208,326**	**196,769**	**94.5**	**193,270**	**98.2**	**3,499**	**1.8**	**92.8**	**1.7**

For peer review only

Table 4: Breakdown of reasons for discarding HES delivery records linked to an ONS birth excluding duplicate copies of HES delivery episodes and invalid deliveries for England singleton births by year

Year	All<br>Total links broken (excluding exact duplicates and invalid records)	a<br>Same mother different birth	b<br>Multiple episodes - pre/post delivery admission	c<br>Multiple episodes - part of spell	d<br>Multiple episodes - exact duplicates with different epikeys	e<br>No match	f<br>Potential multiple birth
2005	46,164	24,457	14,941	5,805	104	817	40
2006	49,706	25,082	16,565	6,984	204	782	89
2007	48,999	26,304	13,187	8,301	323	799	85
2008	47,748	26,032	9,466	11,473	89	614	74
2009	40,519	25,688	6,137	7,680	283	673	58
2010	37,007	24,993	5,176	5,819	366	599	54
2011	37,013	24,679	6,033	5,367	741	145	48
2012	36,176	24,966	5,899	4,298	838	146	29
2013	34,285	23,071	5,709	5,018	281	169	37
2014	31,690	21,980	3,662	3,957	1,863	110	118
**All years**	**409,307**	**247,252**	**86,775**	**64,702**	**5,092**	**4,854**	**632**
Percentage	***100***	***60.41***	***21.2***	***15.81***	***1.24***	***1.19***	***0.15***

For peer review only

For peer review only - http://bmjopen.bmj.com/site/about/guidelines.xhtml

Table 5: Comparison of differences in the percentage distributions of values for key variables for ONS birth records that remained correctly linked to one HES delivery record after the quality assurance procedure, and the ONS birth records with all links to HES discarded after the quality assurance, for singleton and multiple births

	Singleton births		Multiple births	
**Variable**	**Value**	**Difference in percentages***	**Value**	**Difference in percentages***
Match rank of linkage algorithm	6	5.4		
Region of birth	Elsewhere	2.3	East of England	1.9
	Home	38.3	London	5.8
			Home	2.8
Year of birth	2006	2.5	2012	7.4
	2007	3.6	2013	6.9
	2009	1.4	2014	7.2
Month of birth	March	2.0	March	1.7
			April	1.5
Day of birth (8)			Saturday	1.2
Day of birth (11)			Saturday	1.2
Hour of birth	3.00 - 5.59	1.7	6.00 - 8.59	1.4
	6.00 - 8.59	1.8	9.00 -11.59	1.5
Sex of baby			Male	1.1
Age of mother	30-34	1.7	30-34	1.2
	35-39	1.3	35-39	2.4
			40-44	3.9
Ethnicity of baby	Not known	3.4	Black African	1.9
			Not known	1.4
Gestational age	Pre-term	1.1	Pre-term	1.1
Stillbirth	Yes	2.3	Yes	2.5
Gestational age missing on HES	Yes	20.1	Yes	10.9
Birthweight missing on HES	Yes	12.3	Yes	7.0
Trust	PPL (Portland)	1.7	PPL (Portland)	2.5
	RGQ (Ipswich)	2.0	RGQ (Ipswich)	1.5
	RKE (Whittington)	4.0	RJ6 (Croydon)	1.3
	RVL (Barnet and Chase Farm)	1.4	RKE (Whittington)	3.9
			RQ8 (Mid Essex)	1.6
			RTH (Oxford University Hospitals)	1.6
			RVL (Barnet and Chase Farm)	1.4
			RVV (East Kent)	2.0
			RW3 (Central Manchester)	1.1
			RWH (East and North Hertfordshire)	1.1

* Over one per cent

For peer review only

For peer review only - http://bmjopen.bmj.com/site/about/guidelines.xhtml

Appendix A

Quality assurance procedures for singleton and multiple births to select only one correct linked HES record for each ONS birth record and discard incorrect linkages (detailed version)

1: Singleton births

RULE	CATEGORY	APPLIES TO	K (Keep the link)	D (Discard the link)	NOTES
1	Cleaning	Linked to HES records that have no birth or op dates or is an invalid delivery		HES records where baby date of birth = null or valid delivery = false	
2	Cleaning	Duplicate HES records (typically x 6 or x 9) linked to same ONS birth record where baby tail information and identifier is identical	The first version of the duplicate HES record goes through to the subsequent cleaning stages	All other versions of the duplicate HES record	Due to IT administrative issue with some NHS Trusts
3	SMDB	ONS birth record linked to HES record where baby date of birth differs between them by 24 weeks (168 days) or more		If HES baby date of birth > 24 weeks (168 days) from ONS birth record baby date of birth	The size of the date difference implies a different birth to the same mother<br><br>Kings Project used 30 weeks
4	SMSB	ONS birth record linked to one HES record (originally or remaining)	If ONS birth and HES variables match as listed in Box 3	If ONS birth and HES variables do not match as listed in Box 3	
5	SMSB	ONS birth record linked to >1 HES record all from same spell with genuine epiorder sequence (or a combination of genuine spell and additional delivery records)	If ONS birth and HES variables match as listed in Box 3 (baby date of birth must be exact match) and episode is part of spell and onset of delivery and delivery method and birth status values are valid	If ONS birth and HES variables do not match as listed in Box 3 or are the other episodes in that spell	Identifies where ONS birth records are linked to >1 HES record because some or all form a hospital spell. A spell is identified as where episodes have a sequence of epiorder numbers (e.g. 1 and 2) and the episode start date of the subsequent episode = the episode end date of the previous episode, all have the same admission date, and the discharge date is only recorded on the final episode.<br><br>The episode in the spell with the maximum and valid delivery information is chosen
6	SMSB	ONS birth record linked to >1 HES record with different epikeys but duplicate (admidate, disdate, epistart, epiend,	If ONS birth and HES variables match as listed in Box 3 and highest epikey	If ONS birth and HES variables do not match as listed in Box 3 or if	Highest epikey is the latest HES record and should contain the most information

For peer review only - http://bmjopen.bmj.com/site/about/guidelines.xhtml

		epiorder, date of birth, gestat, birweit, baby sex, numbaby, birstat, birordr, delonset, delmeth, ICD code count, OP code count, procode, sitetret)		not highest epikey	
7	SMSB	ONS birth record linked to >1 HES record with different epikeys but duplicate (epiorder, date of birth, gestat, birweit, baby sex, numbaby, birstat, birordr, delonset, delmeth, procode, sitetret)<br><br>Different admidate, disdate, epistart, epiend, ICD code count, OP code count	If ONS birth and HES variables match as listed in Box 3 and highest epikey	If ONS birth and HES variables do not match as listed in Box 3 or if not highest epikey	Highest epikey is the latest HES record and should contain the most information<br><br>The additional HES records can be pre- or post- hospital admissions relating to a delivery
8	SMSB	ONS birth record linked to >1 HES record with different epikeys not duplicates	If ONS birth and HES variables match as listed in Box 3 (baby date of birth must be exact match) and ONS and onset of delivery and delivery method and birth status and birth order values are valid	If ONS birth and HES variables match as listed in Box 3 but baby date of birth is not an exact match or HES baby date of birth is null) and onset of delivery and delivery method and birth status and birth order values are not valid and there is a corresponding K record	Chooses the linked HES record with the maximum valid delivery information
10	SMSB	ONS birth record linked to 2 HES records, both match on baby date of birth and location of birth	If ONS birth and HES variables match as listed in Box 3 (HES derived and original baby date of birth must be exact match to ONS) and onset of delivery and delivery method and birth status and birth order values are valid. Most escalated onset of delivery and delivery method	If ONS birth and HES variables match as listed in Box 3 (HES derived baby date of birth is exact match to ONS but HES original baby date of birth is null) and onset of delivery and delivery method and birth status and birth order values are valid (but less escalated)	A small number of records that can be differentiated by one containing maximum delivery information and one doesn't where they both match on location of birth and baby dob
11	SMSB	ONS birth record linked to HES records with differing baby date of birth values (within 14	If ONS birth and HES variables match as listed in Box 3 (baby date of birth	If HES baby date of birth <=-14 days from ONS baby date of birth	Differentiates between the HES linked records by HES baby date of birth. The HES records that have HES baby date of

For peer review only - http://bmjopen.bmj.com/site/about/guidelines.xhtml

For peer review only

		days of ONS baby date of birth)	must be exact match) and valid onset of delivery and delivery method value	or HES baby date of birth <=-1 and >-14 days before ONS baby date of birth<br><br>or<br><br>if already a K record and HES baby date of birth >=1-14 days from ONS baby date of birth	birth <=-14 days from ONS baby date of birth, then <=-1 and >-14 days before ONS baby date of birth, then >=1-14 days from ONS baby date of birth, are discarded and the remaining 1 linked HES record with HES baby date of birth = ONS baby date of birth and valid method of delivery value is kept (if matching).<br><br>The discarded HES records look like maternal pregnancy care records based on ICD codes
12	SMWB	ONS birth record linked to HES records with differing baby date of birth values (within 15 to 168 days of ONS baby date of birth)	If ONS birth and HES variables match as listed in Box 3 (baby date of birth must be exact match) and valid onset of delivery and delivery method value	If already a K record and HES baby date of birth >14-168 days from ONS baby date of birth and different baby tail values	The discarded records look like different births approximately 2 or 3 months later to the same mother, some of which are clearly premature births.<br><br>Could have been incorporated in Rule 3
13		ONS birth record linked to >1 HES record with different epikeys but duplicate (date of birth, gestat, birweit, baby sex, delonset, delmeth)<br><br>]]	If ONS birth and HES variables match as listed in Box 3 and highest epikey	If not highest epikey	
14	SMSB	Clerical check to identify potential multiple birth records where >1 HES baby tail with same epikey linking to ONS birth record and/or where HES multibirth ICD is true and/or the epikey is linked to >1 ONS birth record, but not due to SMDB	Keep as a singleton matched birth if ONS birth and HES variables match as listed in Box 3<br><br>and<br><br>HES multiple birth ICD but matches only one ONS birth record<br><br>or<br><br>Either ONS birth or HES say multiple or stillbirth, baby tails identical, keep only 1 HES record with field order 1 (genuine duplicates)<br><br>or<br><br>ONS birth singleton and HES multiple,	Identify as potential multiple birth if:<br><br>epikey links >1 ONS birth record with same baby date of birth<br><br>or<br><br>2 different HES baby tails, 1 still birth, and only 1 ONS birth record for livebirth<br><br>or<br><br>HES multiple birth ICD and HES baby tails different	Any records identified as potential multiple births are separated and given to ONS to check

For peer review only - http://bmjopen.bmj.com/site/about/guidelines.xhtml

			treat as singleton if baby tails are the same	D all if ONS birth and HES variables do not match as listed in Box 3	
15	Check	If previous stages have kept > correct number of linked HES records per ONS birth	The highest epikey	Version (s) with earlier or other epikeys	
16	Check	If previous stages have kept no links per ONS birth	If birth location match, ONS baby date of birth within HES admission date ranges, and gestation, birth weight and sex match or are null and match rank 1 and is not a SMDB or invalid record.		Clerical
			Or if birth location does not match but everything else does		

For peer review only

2: Multiple Births

RULE	CATEGORY	APPLIES TO	K (Keep the link)	D (Discard the link)	NOTES
1	Cleaning	Linked to HES records that are invalid deliveries		HES records where valid delivery = false	Do not exclude those where HES baby date of birth = null because may be ghost baby tail records
2	Cleaning	Duplicate HES records (typically x 6 or x 9) linked to same ONS birth record where baby tail information and identifier is identical	The first version of the duplicate HES record goes through to the subsequent cleaning stages	All other versions of the duplicate HES record	Due to IT administrative issue with some NHS Trusts
3	SMDB	ONS birth record linked to HES record where baby date of birth differs between them by 24 weeks (168 days) or more		If HES baby date of birth > 24 weeks (168 days) from ONS birth record baby date of birth	The size of the date difference implies a different birth to the same mother<br><br>Kings Project used 30 weeks
4	SMSB	ONS birth records in MULTBTNO linked to one epikey and the correct number of baby tails (originally or remaining)<br><br>Correct number of baby tails<br><br>Correct number of ONS birth records<br><br>1 epikey per MULTBTNO	If ONS birth and HES variables match as listed in Box 3 and field order 1 for 1st baby, field order 2 for 2nd baby etc. so that ONS birth record corresponds to correct baby tail	If ONS birth and HES variables do not match as listed in Box 3.<br><br>Or field and birth orders don't match and sex and birth weight are wrong way round and already 2 correct K records	
5	SMSB	ONS birth records in MULTBTNO linked to one epikey and more than the correct number of baby tails<br><br>> correct number of baby tails<br><br>(duplicates)	If ONS birth and HES variables match as listed in Box 3 and field order 1 (or lowest) for 1st baby, field order 2 (or plus 1) for 2nd baby etc. so that birth record corresponds to correct baby tail	If ONS birth and HES variables do not match as listed in Box 3.<br><br>Or field and birth orders don't match and sex and birth weight are wrong way round and already 2 correct K records	

For peer review only - http://bmjopen.bmj.com/site/about/guidelines.xhtml

		Correct number of ONS birth records<br><br>1 epikey per MULTBTNO			
6	SMSB	ONS birth records in MULTBTNO linked to more than one epikey<br><br>Correct number of ONS birth records<br><br>>1 epikey per MULTBTNO	Where epikey matches by baby date of birth, ONS baby date of birth is within HES admission date range, and has valid/most escalated onset of delivery and delivery method	All others	Births with >1 epikey correctly associated with them, but not spells<br><br>Due to first baby linked to 1 epikey and the second baby to the other or duplicates with epikey number increasing by one each time, or non-delivery records
7	SMSB	Remaining MULTBTNO with < correct number of ONS birth records	If ONS birth and HES variables match as listed in Box 3		Some of the missing ONS birth records found in singleton file and unlinked file
8	Check	If previous stages have kept > correct number of links per MULTBTNO. Epikeys are exact duplicates	Version with highest epikey or version within admission date range	Version (s) with earlier or other epikeys	
9	Check	If previous stages have kept no links per MULTBTNO	If birth location match, ONS baby date of birth within HES admission date ranges, and gestation, birth weight and sex match or are null and match rank 1 and is not a SMDB or invalid record<br><br>Or if birth location does not match but everything else does		Clerical

For peer review only

For peer review only - http://bmjopen.bmj.com/site/about/guidelines.xhtml

Appendix B

Differences between records with correct and incorrect linkage

Comparison of distributions of key variables for singleton and multiple births in England 2005 to 2014, for 4 groups of data:

1) All birth registrations (n = 6,468,586 singleton births, n = 208,326 multiple births)

2) All birth registrations linked to HES by NHS Digital with a HES delivery record (n = 6,268,013 singleton births, n = 196,769 multiple births)

3) Those of 2) that remain linked to HES after QA (n = 6,138,487 singleton births, n = 193,270 multiple births)

4) Those of 2) left with no link after QA (n = 129,526 singleton births, n = 3,499 multiple births)

Tables B.13 and B.14 are based on total links (a birth registration record can link to >1 HES record):

1) All birth registrations (n = 6,468,586 singleton births, n = 208,326 multiple births)

2) All birth registrations and HES links by NHS Digital (n = 7,862,903 singleton births, n = 395,202 multiple births)

3) Those of 2) that remain linked to HES after QA (n = 6,138,487 singleton births, n = 193,270 multiple births)

4) Those of 2) total broken links after QA (n = 1,724,416 singleton births, n = 201,932 multiple births)

Total number of links are inflated for multiple births because each birth registration within a multiple delivery links with every possible HES baby tail i.e. 4 linked records minimum

Chi-square tests are conducted to test the null hypothesis that the distribution of a demographic variable is the same in the “linked to HES after QA” as in the “No link to HES after QA"

Any rows with values of 0 are excluded from the Chi-square tests

For peer review only - http://bmjopen.bmj.com/site/about/guidelines.xhtml

	Singleton births England				Multiple births England				Difference between no link to HES and linked to HES after QA	
	Percentage distribution of variables				Percentage distribution of variables				Singletons	Multiples
**B.1: Match rank**	**All birth registrations**	**All birth registrations linked to HES by NHS Digital with a HES delivery record**	**Linked to HES after QA**	**No link to HES after QA**	**All birth registrations**	**All birth registrations linked to HES by NHS Digital with a HES delivery record**	**Linked to HES after QA**	**No link to HES after QA**		
1	80.51	81.92	82.06	75.13	80.3	81.4	81.4	80.9	-6.93	-0.59
2	2.65	2.67	2.65	3.68	2.0	2.1	2.1	2.8	1.03	0.71
3	1.39	1.42	1.42	1.27	1.3	1.3	1.3	1.1	-0.15	-0.16
4	0.06	0.06	0.06	0.09	0.0	0.0	0.0	0.1	0.03	0.08
5	0.45	0.46	0.46	0.51	0.4	0.4	0.4	0.5	0.06	0.14
6	13.26	13.16	13.05	18.40	14.3	14.4	14.4	13.6	5.35	-0.85
7	0.28	0.27	0.27	0.38	0.3	0.3	0.3	0.1	0.11	-0.25
8	0.05	0.04	0.03	0.53	0.0	0.0	0.0	0.9	0.50	0.93
Null	1.36	0.00	0.00	0.00	1.3	0.0	0.0	0.0	0.00	0.00
**Total**	6,468,586	6,268,013	6,138,487	129,526	208,326	196,769	193,270	3,499		
**χ^2 test**	$p < .001$ (χ^2 = 11815.13 df = 7)				$p < .001$ (χ^2 = 903.69, df = 7)					
**B.2: Region of birth**										
East Midlands	6.84	6.95	7.02	3.56	6.61	6.66	6.64	7.66	-3.46	1.02
East of England	10.05	10.08	10.12	8.09	10.50	10.46	10.43	12.35	-2.03	1.92
London	19.36	18.92	18.93	18.34	21.91	21.14	21.03	26.86	-0.59	5.83
North East	4.61	4.66	4.73	0.97	4.62	4.75	4.81	1.54	-3.77	-3.26
North West	13.02	13.18	13.32	6.63	12.35	12.49	12.52	10.89	-6.69	-1.63
South Central	6.92	7.03	7.08	4.56	7.39	7.47	7.47	7.69	-2.52	0.22
South East Coast	7.68	7.76	7.83	4.38	8.48	8.53	8.52	8.72	-3.45	0.19
South West	8.48	8.63	8.73	3.74	8.91	9.17	9.25	4.74	-5.00	-4.51
West Midlands	10.64	10.78	10.93	3.83	10.03	10.05	10.09	7.75	-7.09	-2.35
Yorkshire/Humber	9.69	9.85	9.97	4.07	8.92	9.05	9.07	8.46	-5.90	-0.61

For peer review only

For peer review only - http://bmjopen.bmj.com/site/about/guidelines.xhtml

For peer review only

Elsewhere	0.17	0.15	0.10	2.34	0.05	0.04	0.04	0.43	2.24	0.39
Home	2.54	2.01	1.22	39.49	0.23	0.19	0.14	2.92	38.27	2.77
Not known	0.00	0.00	0.00	0.00	0.00	0.00	0	0	0.00	0.00
**Total**	6,468,586	6,268,013	6,138,487	129,526	208,326	196,769	193,270	3,499		
**χ^2 test**	p < .001 (χ^2 = 992568.53, df = 11)				p < .001 (χ^2 = 1768.25, df = 11)					
**B.3: Year of birth**										
2005	9.26	9.02	9.03	8.49	8.82	8.84	8.90	5.57	-0.55	-3.33
2006	9.60	9.40	9.35	11.86	9.38	9.41	9.45	7.55	2.51	-1.90
2007	9.88	9.78	9.70	13.28	9.63	9.79	9.81	8.57	3.57	-1.23
2008	10.14	10.14	10.12	11.12	9.99	10.22	10.29	5.89	1.00	-4.41
2009	10.10	10.15	10.12	11.47	10.56	10.84	10.91	7.23	1.35	-3.68
2010	10.29	10.41	10.45	8.78	10.32	10.69	10.79	5.32	-1.67	-5.47
2011	10.30	10.43	10.46	8.72	10.61	10.77	10.80	9.35	-1.75	-1.45
2012	10.46	10.55	10.56	10.04	10.59	10.25	10.12	17.49	-0.52	7.37
2013	10.01	10.10	10.15	7.94	9.97	9.52	9.40	16.29	-2.21	6.89
2014	9.95	10.02	10.06	8.31	10.13	9.66	9.54	16.75	-1.74	7.21
**Total**	6,468,586	6,268,013	6,138,487	129,526	208,326	196,769	193,270	3,499		
**χ^2 test**	p < .001 (χ^2 = 4645.84, df = 9)				p < .001 (χ^2 = 811.59, df = 9)					
**B.4: Month of birth**										
Jan	8.28	8.27	8.26	8.46	8.35	8.38	8.39	8.09	0.20	-0.30
Feb	7.59	7.58	7.56	8.40	7.57	7.56	7.57	6.97	0.84	-0.60
March	8.29	8.26	8.21	10.19	8.22	8.20	8.17	9.89	1.98	1.72
April	8.00	7.99	8.00	7.56	7.95	7.97	7.94	9.43	-0.44	1.49
May	8.43	8.44	8.45	7.72	8.38	8.38	8.39	7.92	-0.74	-0.48
June	8.27	8.28	8.29	7.91	8.55	8.52	8.52	8.57	-0.38	0.06
July	8.68	8.69	8.70	8.10	8.68	8.70	8.70	8.92	-0.60	0.22
Aug	8.60	8.61	8.62	8.17	8.07	8.14	8.15	7.57	-0.45	-0.58
Sep	8.65	8.67	8.68	8.21	8.39	8.43	8.46	6.89	-0.47	-1.57
Oct	8.67	8.69	8.70	8.24	8.97	8.91	8.91	9.03	-0.46	0.12
Nov	8.21	8.22	8.22	8.08	8.43	8.40	8.41	7.89	-0.14	-0.53
Dec	8.32	8.32	8.30	8.95	8.44	8.39	8.38	8.83	0.65	0.45
**Total**	6,468,586	6,268,013	6,138,487	129,526	208,326	196,769	193,270	3,499		

**χ^2 test**		p < .001 (χ^2 = 1072.06, df = 11)				p < .001 (χ^2 = 42.23, df = 11)				
**B.5: Day of birth (8)**										
Mon	13.25	13.25	13.25	13.27	13.75	13.76	13.74	14.49	0.02	0.75
Tue	14.63	14.64	14.64	14.53	16.41	16.38	16.39	15.72	-0.11	-0.67
Wed	14.90	14.90	14.90	14.86	16.74	16.80	16.80	16.29	-0.04	-0.51
Thur	15.03	15.03	15.04	14.87	17.31	17.29	17.29	17.63	-0.17	0.35
Fri	14.73	14.73	14.73	14.65	16.66	16.65	16.68	15.06	-0.08	-1.62
Sat	12.96	12.95	12.94	13.32	9.77	9.76	9.74	10.95	0.38	1.21
Sun	12.14	12.15	12.15	12.10	7.75	7.75	7.75	8.00	-0.05	0.25
Hol	2.36	2.36	2.36	2.40	1.61	1.62	1.61	1.86	0.04	0.25
**Total**	6,468,586	6,268,013	6,138,487	129,526	208,326	196,769	193,270	3,499		
**χ^2 test**		p < .001 (χ^2 = 36.20, df = 7)				p = .0333 (χ^2 = 15.22, df = 7)				
**B.6: Day of birth (11)**										
Mon	13.05	13.05	13.05	12.99	13.54	13.55	13.54	14.32	-0.07	0.78
Tue	13.32	13.33	13.33	13.26	14.95	14.92	14.93	14.58	-0.06	-0.35
Wed	14.59	14.59	14.59	14.46	16.37	16.43	16.44	16.00	-0.12	-0.43
Thur	14.55	14.56	14.56	14.24	16.78	16.76	16.76	16.95	-0.32	0.19
Fri	13.59	13.59	13.59	13.55	15.39	15.37	15.40	13.63	-0.04	-1.77
Sat	12.96	12.95	12.94	13.47	9.77	9.76	9.74	10.95	0.53	1.21
Sun	12.14	12.15	12.15	12.24	7.75	7.75	7.75	8.00	0.09	0.25
a.hol	1.68	1.68	1.69	1.62	1.89	1.88	1.88	1.60	-0.07	-0.28
b.hol	1.75	1.75	1.75	1.72	1.95	1.96	1.96	2.11	-0.03	0.16
Christmas	0.43	0.43	0.43	0.50	0.29	0.29	0.29	0.37	0.07	0.09
Hol	1.93	1.93	1.93	1.95	1.33	1.33	1.33	1.49	0.03	0.16
**Total**	6,468,586	6,268,013	6,138,487	129,526	208,326	196,769	193,270	3,499		
**χ^2 test**		p < .001 (χ^2 = 60.47, df = 10)				p = .0525 (χ^2 = 18.15, df = 10)				
**B.7: Hour of birth**										
0.00 - 2.59	12.35	12.36	12.35	12.84	8.42	8.46	8.45	9.00	0.49	0.56
3.00 - 5.59	12.15	12.15	12.12	13.75	7.74	7.82	7.81	8.06	1.64	0.25
6.00 - 8.59	10.79	10.76	10.73	12.53	6.67	6.69	6.67	8.03	1.80	1.36
9.00 -11.59	16.06	16.10	16.13	14.39	26.62	26.56	26.53	27.98	-1.74	1.45

For peer review only

12.00 -14.59	12.88	12.87	12.90	11.80	17.35	17.27	17.28	16.95	-1.10	-0.33
15.00 -17.59	11.86	11.85	11.87	10.93	13.41	13.37	13.40	11.77	-0.94	-1.63
18.00 -20.59	11.19	11.18	11.19	11.02	9.65	9.65	9.67	8.49	-0.16	-1.19
21.00 -23.59	11.81	11.81	11.81	11.80	9.18	9.21	9.22	8.69	-0.01	-0.53
Null	0.91	0.91	0.91	0.93	0.96	0.96	0.96	1.03	0.03	0.07
**Total**	6,468,586	6,268,013	6,138,487	129,526	208,326	196,769	193,270	3,499		
**χ^2 test**	p < .001 (χ^2 = 1146.18, df = 8)				p = < .001 (χ^2 = 26.99, df = 8)					
**B.8: Age of mother**										
Under 15	0.03	0.03	0.03	0.02	0.01	0.01	0.01	0.00	-0.01	-0.01
15-19	3.16	3.16	3.18	2.28	1.24	1.26	1.27	0.86	-0.89	-0.41
20-24	18.48	18.57	18.59	17.39	11.14	11.41	11.46	8.75	-1.20	-2.72
25-29	27.29	27.42	27.42	27.44	22.70	23.10	23.19	18.12	0.01	-5.07
30-34	28.72	28.73	28.70	30.39	32.75	32.85	32.83	33.98	1.69	1.16
35-39	16.13	15.99	15.96	17.20	23.75	23.44	23.40	25.81	1.24	2.41
40-44	3.60	3.53	3.54	3.19	5.99	5.64	5.57	9.46	-0.35	3.89
45 & more	0.20	0.19	0.19	0.19	1.34	1.17	1.15	2.14	0.00	0.99
Null	2.39	2.39	2.40	1.91	1.08	1.12	1.12	0.89	-0.49	-0.24
**Total**	6,468,586	6,268,013	6,138,487	129,526	208,326	196,769	193,270	3,499		
**χ^2 test**	p < .001 (χ^2 = 716.36, df = 8)				p < .001 (χ^2 = 196.19, df = 8)					
**B.9: Sex of baby**										
Female	48.69	48.69	48.66	49.65	49.87	49.91	49.93	48.84	0.99	-1.09
Male	51.31	51.31	51.34	50.35	50.13	50.09	50.07	51.16	-0.99	1.09
**Total**	6,468,586	6,268,013	6,138,487	129,526	208,326	196,769	193,270	3,499		
**χ^2 test**	p < .001 (χ^2 = 49.40, df = 1)				p = .2018 (χ^2 = 1.63, df = 1)					
**B.10: Ethnicity of baby**										
Bangladeshi	1.41	1.43	1.43	1.00	0.86	0.89	0.89	0.69	-0.43	-0.20
Indian	3.00	3.02	3.04	1.90	2.73	2.62	2.60	3.63	-1.14	1.03
Pakistani	4.12	4.15	4.18	2.81	2.98	2.99	2.98	3.06	-1.37	0.07
Black African	3.21	3.14	3.12	3.90	3.73	3.67	3.64	5.54	0.77	1.91
Black Caribbean	0.97	0.97	0.97	0.91	1.09	1.09	1.10	0.97	-0.06	-0.13
White British	63.44	63.99	63.97	64.75	65.11	65.85	65.90	62.79	0.78	-3.12

For peer review only

White Other	8.19	8.10	8.13	6.75	8.38	8.21	8.23	7.14	-1.38	-1.08
Other	9.43	9.34	9.35	8.71	8.85	8.65	8.65	8.69	-0.64	0.03
Not Known	6.20	5.87	5.80	9.21	6.26	6.02	5.99	7.40	3.41	1.41
Null	0.02	0.01	0.01	0.07	0.02	0.01	0.01	0.09	0.06	0.08
**Total**	6,468,586	6,268,013	6,138,487	129,526	208,326	196,769	193,270	3,499		
**χ^2 test**	$p < .001$ ($\chi^2 = 4730.98$ df = 9)				$p < .001$ ($\chi^2 = 151.29$, df = 9)					
**B.11: Gestational age**										
Missing or less than 22 weeks	0.79	0.78	0.76	1.29	0.93	0.92	0.90	1.83	0.53	0.92
Pre-term	6.08	6.07	6.05	7.13	54.10	53.86	53.84	54.93	1.08	1.09
Term	89.17	89.18	89.19	88.63	44.85	45.09	45.12	43.13	-0.56	-2.00
Post-term	3.96	3.97	4.00	2.95	0.13	0.13	0.13	0.11	-1.04	-0.01
**Total**	6,468,586	6,268,013	6,138,487	129,526	208,326	196,769	193,270	3,499		
**χ^2 test**	$p < .001$ ($\chi^2 = 1048.57$, df = 3)				$p < .001$ ($\chi^2 = 35.71$, df = 3)					
**B.12: Stillbirth**										
No	99.52	99.53	99.58	97.30	98.87	98.88	98.92	96.46	-2.28	-2.47
Yes	0.48	0.47	0.42	2.70	1.13	1.12	1.08	3.54	2.28	2.47
**Total**	6,468,586	6,268,013	6,138,487	129,526	208,326	205,684	193,270	12,414		
**χ^2 test**	$p < .001$ ($\chi^2 = 14053.04$, df = 1)				$p < .001$ ($\chi^2 = 188.14$, df = 1)					
**B.13: Gestational age missing on HES**	**All birth registrations**	**All birth registrations and HES links by NHS Digital**	**Linked to HES after QA**	**Broken links after QA**	**All birth registrations**	**All birth registrations and HES links by NHS Digital**	**Linked to HES after QA**	**Broken links after QA**		
No		68.94	73.50	52.70		74.18	79.73	68.86	-20.80	-10.87
Yes		31.06	26.50	47.30		25.82	20.27	31.14	20.80	10.87
**Total**	6,468,586	7,862,903	6,138,487	1,724,416	208,326	196,769	193,270	3,499		
**χ^2 test**	$p < .001$ ($\chi^2 = 272051.09$ df = 1)				$p < .001$ ($\chi^2 = 6086.08$, df = 1)					
**B.14: Birth weight missing on HES**										
No		80.69	83.38	71.12		85.88	89.52	82.40	-12.26	-7.13
Yes		19.31	16.62	28.88		14.12	10.48	17.60	12.26	7.13

For peer review only - http://bmjopen.bmj.com/site/about/guidelines.xhtml

**Total**	6,468,586	7,862,903	6,138,487	1,724,416	208,326	395,202	193,270	201,932
**χ^2 test**	p < .001 (χ^2 = 129812.25 df = 1)				p < .001 (χ^2 = 4814.69, df = 1)			

	**Singleton births England** Percentage distribution of variables				**Multiple births England** Percentage distribution of variables					
**B.15: Location of birth (assigned trust code)**	All birth registrations	All birth registrations linked to HES by NHS Digital with a HES delivery record	Linked to HES after QA	No link to HES after QA	All birth registrations	All birth registrations linked to HES by NHS Digital with a HES delivery record	Linked to HES after QA	No link to HES after QA		
**MMC**	0.00	0.00	0.00	0.00	0.00	0.00	0.00	0.00	0.00	0.00
**MML**	0.07	0.00	0.00	0.04	0.04	0.00	0.00	0.00	0.04	0.00
**MMS**	0.00	0.00	0.00	0.00	0.00	0.00	0.00	0.00	0.00	0.00
**PPJ**	0.04	0.01	0.00	0.26	0.00	0.00	0.00	0.06	0.26	0.06
**PPL**	0.30	0.04	0.00	1.74	0.40	0.05	0.00	2.52	1.74	2.51
**PPN**	0.00	0.00	0.00	0.01	0.00	0.00	0.00	0.00	0.01	0.00
**PPT**	0.00	0.00	0.00	0.08	0.00	0.00	0.00	0.00	0.08	0.00
**R1F**	0.18	0.18	0.18	0.27	0.18	0.18	0.18	0.31	0.09	0.13
**RA2**	0.48	0.49	0.49	0.21	0.57	0.59	0.60	0.23	-0.28	-0.37
**RA4**	0.22	0.23	0.23	0.11	0.18	0.19	0.18	0.37	-0.12	0.19
**RA7**	0.84	0.86	0.87	0.35	0.84	0.89	0.90	0.23	-0.52	-0.67
**RA9**	0.35	0.35	0.35	0.10	0.33	0.33	0.33	0.11	-0.25	-0.22
**RAE**	0.89	0.91	0.92	0.43	0.66	0.69	0.68	1.00	-0.49	0.32
**RAJ**	0.54	0.55	0.55	0.24	0.57	0.56	0.55	0.86	-0.31	0.31
**RAL**	0.48	0.48	0.49	0.13	0.44	0.44	0.44	0.34	-0.36	-0.10
**RAP**	0.55	0.55	0.55	0.21	0.49	0.49	0.49	0.34	-0.34	-0.15
**RAS**	0.60	0.61	0.62	0.15	0.57	0.59	0.59	0.40	-0.47	-0.19
**RAX**	0.83	0.84	0.85	0.32	0.99	0.93	0.93	1.23	-0.52	0.30
**RBA**	0.47	0.47	0.48	0.27	0.55	0.55	0.55	0.46	-0.21	-0.09
**RBD**	0.30	0.31	0.32	0.04	0.31	0.32	0.33	0.14	-0.28	-0.18
**RBK**	0.61	0.63	0.64	0.13	0.50	0.51	0.51	0.29	-0.50	-0.23
**RBL**	0.52	0.53	0.54	0.14	0.52	0.53	0.53	0.26	-0.40	-0.28

For peer review only

For peer review only - http://bmjopen.bmj.com/site/about/guidelines.xhtml

**RBN**	0.50	0.49	0.50	0.08	0.41	0.41	0.41	0.06	-0.43	-0.36
**RBT**	0.42	0.42	0.42	0.45	0.39	0.39	0.39	0.37	0.02	-0.02
**RBZ**	0.23	0.24	0.24	0.10	0.21	0.22	0.22	0.29	-0.14	0.07
**RC1**	0.46	0.46	0.46	0.39	0.48	0.46	0.46	0.60	-0.07	0.14
**RC3**	0.43	0.40	0.40	0.09	0.35	0.33	0.33	0.40	-0.32	0.07
**RC9**	0.74	0.75	0.76	0.50	0.81	0.80	0.80	0.66	-0.25	-0.15
**RCB**	0.49	0.50	0.51	0.05	0.48	0.51	0.51	0.34	-0.46	-0.17
**RCC**	0.25	0.25	0.23	1.04	0.17	0.17	0.16	0.69	0.81	0.52
**RCD**	0.29	0.30	0.31	0.04	0.25	0.26	0.27	0.00	-0.27	-0.27
**RCF**	0.36	0.37	0.38	0.08	0.31	0.32	0.32	0.06	-0.30	-0.27
**RCX**	0.34	0.34	0.35	0.05	0.33	0.34	0.34	0.06	-0.30	-0.29
**RD1**	0.72	0.72	0.72	0.43	0.74	0.75	0.76	0.34	-0.29	-0.42
**RD3**	0.72	0.73	0.74	0.18	0.72	0.74	0.75	0.26	-0.57	-0.49
**RD7**	0.75	0.76	0.78	0.16	0.69	0.69	0.68	0.74	-0.61	0.06
**RD8**	0.55	0.55	0.54	1.02	0.52	0.51	0.50	1.23	0.48	0.73
**RDD**	0.64	0.66	0.67	0.11	0.61	0.64	0.65	0.14	-0.57	-0.51
**RDE**	0.56	0.57	0.57	0.31	0.58	0.58	0.57	0.80	-0.26	0.23
**RDU**	0.72	0.74	0.75	0.31	0.79	0.80	0.80	0.69	-0.43	-0.11
**RE9**	0.22	0.23	0.23	0.05	0.16	0.17	0.17	0.06	-0.18	-0.11
**REF**	0.62	0.64	0.64	0.30	0.59	0.61	0.62	0.34	-0.34	-0.27
**REP**	1.22	1.24	1.26	0.20	1.59	1.64	1.66	0.74	-1.07	-0.91
**RF4**	1.35	1.32	1.35	0.34	1.35	1.31	1.31	1.09	-1.01	-0.23
**RFF**	0.42	0.43	0.43	0.11	0.37	0.38	0.39	0.11	-0.33	-0.27
**RFR**	0.42	0.42	0.42	0.12	0.41	0.41	0.41	0.46	-0.30	0.05
**RFS**	0.43	0.44	0.45	0.09	0.38	0.40	0.41	0.11	-0.36	-0.29
**RFW**	0.64	0.63	0.62	1.13	0.54	0.49	0.47	1.23	0.51	0.76
**RGC**	0.77	0.76	0.77	0.33	0.74	0.72	0.72	0.80	-0.43	0.08
**RGN**	0.64	0.65	0.66	0.11	0.60	0.60	0.60	0.63	-0.54	0.03
**RGP**	0.32	0.33	0.33	0.06	0.29	0.29	0.29	0.17	-0.27	-0.12
**RGQ**	0.54	0.52	0.48	2.48	0.53	0.49	0.46	1.94	2.00	1.48
**RGR**	0.37	0.38	0.39	0.04	0.35	0.36	0.36	0.17	-0.34	-0.19
**RGT**	0.80	0.82	0.84	0.08	1.05	1.10	1.11	0.11	-0.76	-1.00
**RH8**	0.58	0.59	0.60	0.16	0.65	0.67	0.68	0.40	-0.44	-0.28
**RHM**	0.85	0.86	0.87	0.45	0.86	0.87	0.88	0.34	-0.42	-0.54
**RHQ**	1.03	1.05	1.07	0.20	1.16	1.14	1.13	1.74	-0.87	0.62

For peer review only - http://bmjopen.bmj.com/site/about/guidelines.xhtml

**RHU**	0.86	0.87	0.88	0.30	0.95	0.99	1.00	0.40	-0.58	-0.60
**RHW**	0.83	0.85	0.86	0.39	0.95	0.99	0.99	0.74	-0.48	-0.25
**RJ1**	0.97	0.98	0.99	0.30	1.24	1.26	1.28	0.49	-0.70	-0.79
**RJ2**	0.55	0.53	0.53	0.46	0.52	0.53	0.53	0.29	-0.07	-0.25
**RJ6**	0.65	0.65	0.65	0.68	0.61	0.61	0.59	1.91	0.03	1.33
**RJ7**	0.74	0.75	0.76	0.26	0.99	1.00	1.01	0.77	-0.50	-0.24
**RJC**	0.40	0.41	0.41	0.14	0.36	0.36	0.36	0.29	-0.27	-0.07
**RJD**	0.31	0.31	0.32	0.08	0.28	0.27	0.28	0.06	-0.24	-0.22
**RJE**	0.85	0.86	0.87	0.29	0.77	0.79	0.80	0.31	-0.58	-0.48
**RJF**	0.54	0.54	0.54	0.46	0.48	0.50	0.50	0.34	-0.09	-0.16
**RJL**	0.69	0.70	0.71	0.46	0.59	0.61	0.61	0.57	-0.24	-0.04
**RJN**	0.30	0.31	0.31	0.02	0.30	0.31	0.32	0.09	-0.29	-0.23
**RJR**	0.47	0.48	0.49	0.08	0.47	0.48	0.49	0.11	-0.41	-0.37
**RJZ**	0.74	0.73	0.73	0.47	1.02	0.97	0.97	0.89	-0.26	-0.09
**RK5**	0.46	0.47	0.48	0.19	0.41	0.43	0.43	0.06	-0.29	-0.38
**RK9**	0.66	0.67	0.66	1.15	0.74	0.72	0.72	1.03	0.49	0.31
**RKB**	0.83	0.85	0.87	0.25	0.89	0.92	0.93	0.11	-0.62	-0.82
**RKE (Whittington)**	0.56	0.48	0.40	4.43	0.59	0.47	0.40	4.34	4.03	3.94
**RL4**	0.57	0.58	0.59	0.12	0.60	0.62	0.62	0.43	-0.47	-0.19
**RLN**	0.50	0.50	0.51	0.06	0.44	0.45	0.46	0.00	-0.45	-0.46
**RLQ**	0.27	0.28	0.29	0.07	0.26	0.25	0.25	0.29	-0.22	0.03
**RLT**	0.35	0.36	0.36	0.05	0.29	0.31	0.31	0.11	-0.32	-0.20
**RLU**	1.11	1.10	1.11	0.52	1.32	1.27	1.26	1.91	-0.58	0.66
**RM1**	0.84	0.86	0.88	0.04	0.82	0.86	0.87	0.20	-0.84	-0.67
**RM2**	0.56	0.56	0.55	1.04	0.55	0.52	0.50	1.43	0.49	0.93
**RM4**	0.11	0.11	0.11	0.24	0.08	0.08	0.08	0.17	0.13	0.09
**RMC**	0.74	0.76	0.78	0.13	0.61	0.63	0.64	0.11	-0.64	-0.53
**RMP**	0.40	0.41	0.41	0.12	0.31	0.32	0.32	0.17	-0.30	-0.15
**RN1**	0.41	0.42	0.43	0.16	0.44	0.44	0.44	0.51	-0.27	0.07
**RN3**	0.63	0.65	0.66	0.17	0.62	0.66	0.66	0.26	-0.49	-0.41
**RN5**	0.42	0.42	0.43	0.07	0.45	0.48	0.48	0.06	-0.36	-0.43
**RN7**	0.59	0.60	0.62	0.07	0.52	0.54	0.55	0.11	-0.54	-0.43
**RNA**	0.68	0.69	0.70	0.21	0.60	0.59	0.60	0.54	-0.50	-0.05
**RNH**	0.82	0.79	0.78	1.18	0.63	0.61	0.61	0.63	0.40	0.02
**RNJ**	0.68	0.69	0.70	0.14	0.57	0.57	0.57	0.49	-0.56	-0.09

For peer review only - http://bmjopen.bmj.com/site/about/guidelines.xhtml

**RNL**	0.47	0.46	0.46	0.51	0.42	0.41	0.41	0.66	0.05	0.25
**RNQ**	0.56	0.57	0.57	0.12	0.54	0.55	0.56	0.09	-0.45	-0.48
**RNS**	0.65	0.66	0.67	0.13	0.72	0.75	0.75	0.57	-0.54	-0.18
**RNZ**	0.34	0.35	0.35	0.08	0.43	0.45	0.45	0.06	-0.27	-0.40
**RP5**	0.79	0.81	0.82	0.13	0.70	0.71	0.71	0.71	-0.69	0.01
**RPA**	0.68	0.69	0.70	0.25	0.73	0.76	0.76	0.51	-0.45	-0.25
**RQ8**	0.64	0.64	0.62	1.30	0.70	0.65	0.63	2.17	0.68	1.55
**RQM**	0.81	0.81	0.82	0.49	1.15	1.11	1.11	1.00	-0.33	-0.11
**RQQ**	0.36	0.37	0.37	0.09	0.34	0.35	0.35	0.11	-0.28	-0.24
**RQW**	0.56	0.55	0.56	0.19	0.59	0.57	0.57	0.69	-0.37	0.12
**RQX**	0.74	0.74	0.73	1.02	0.97	0.97	0.97	0.86	0.29	-0.12
**RR1**	1.60	1.64	1.66	0.62	1.34	1.38	1.39	0.89	-1.04	-0.50
**RR7**	0.28	0.28	0.29	0.05	0.29	0.29	0.29	0.29	-0.24	0.00
**RR8**	1.40	1.38	1.39	0.64	1.47	1.43	1.43	1.34	-0.76	-0.08
**RRF**	0.45	0.45	0.46	0.20	0.42	0.43	0.43	0.40	-0.26	-0.03
**RRV**	0.74	0.75	0.76	0.37	1.35	1.35	1.36	0.94	-0.38	-0.42
**RTD**	0.97	0.98	1.00	0.26	1.25	1.29	1.30	0.57	-0.74	-0.73
**RTE**	0.93	0.95	0.97	0.11	0.98	1.03	1.04	0.17	-0.86	-0.87
**RTF**	0.47	0.48	0.49	0.08	0.34	0.35	0.36	0.00	-0.42	-0.36
**RTG**	0.84	0.86	0.87	0.32	0.79	0.79	0.78	1.17	-0.55	0.39
**RTH**	1.26	1.28	1.27	1.43	1.56	1.52	1.50	3.06	0.16	1.56
**RTK**	0.59	0.60	0.61	0.07	0.77	0.79	0.81	0.06	-0.54	-0.75
**RTP**	0.64	0.63	0.64	0.31	0.70	0.69	0.69	0.71	-0.33	0.02
**RTR**	0.80	0.80	0.81	0.20	0.91	0.93	0.95	0.17	-0.61	-0.77
**RTX**	0.51	0.52	0.52	0.25	0.41	0.42	0.43	0.23	-0.27	-0.20
**RV8**	0.74	0.75	0.76	0.27	0.61	0.59	0.60	0.49	-0.49	-0.11
**RVJ**	0.87	0.89	0.91	0.21	1.02	1.05	1.06	0.29	-0.70	-0.77
**RVL**	0.96	0.93	0.90	2.27	0.95	0.89	0.87	2.26	1.37	1.39
**RVR**	0.75	0.75	0.76	0.27	0.86	0.85	0.86	0.63	-0.49	-0.23
**RVV**	1.03	1.03	1.02	1.90	1.05	1.00	0.96	3.14	0.89	2.19
**RVW**	0.53	0.53	0.54	0.08	0.51	0.53	0.54	0.06	-0.46	-0.48
**RVY**	0.45	0.44	0.45	0.09	0.39	0.39	0.39	0.57	-0.37	0.18
**RW3**	1.27	1.27	1.28	0.93	1.48	1.43	1.41	2.52	-0.35	1.11
**RW6**	1.52	1.55	1.58	0.24	1.20	1.24	1.25	0.77	-1.34	-0.47
**RWA**	0.84	0.86	0.87	0.16	0.79	0.81	0.82	0.37	-0.71	-0.45

For peer review only - http://bmjopen.bmj.com/site/about/guidelines.xhtml

For peer review only

**RWD**	0.86	0.86	0.85	0.97	0.72	0.74	0.73	1.26	0.12	0.53
**RWE**	1.54	1.56	1.57	0.96	1.44	1.42	1.40	2.37	-0.61	0.97
**RWF**	0.76	0.77	0.78	0.33	0.98	1.01	1.02	0.51	-0.45	-0.50
**RWG**	0.82	0.84	0.85	0.33	0.91	0.89	0.88	1.03	-0.52	0.15
**RWH**	0.80	0.80	0.78	1.73	0.94	0.94	0.92	2.00	0.95	1.08
**RWJ**	0.54	0.56	0.57	0.08	0.52	0.55	0.55	0.40	-0.49	-0.15
**RWP**	0.87	0.87	0.89	0.28	0.89	0.83	0.84	0.71	-0.60	-0.12
**RWW**	0.48	0.49	0.50	0.22	0.44	0.45	0.46	0.23	-0.28	-0.23
**RWY**	0.86	0.87	0.88	0.43	0.78	0.79	0.79	0.86	-0.46	0.07
**RX1**	1.50	1.53	1.55	0.77	1.60	1.58	1.57	2.03	-0.78	0.46
**RXC**	0.58	0.59	0.60	0.13	0.49	0.49	0.49	0.54	-0.47	0.06
**RXF**	0.98	1.00	1.02	0.19	0.79	0.83	0.84	0.20	-0.83	-0.64
**RXH**	0.83	0.82	0.83	0.33	1.08	1.07	1.06	1.51	-0.50	0.45
**RXK**	0.89	0.89	0.90	0.43	0.68	0.67	0.66	1.00	-0.47	0.34
**RXL**	0.44	0.45	0.46	0.16	0.39	0.41	0.41	0.17	-0.30	-0.24
**RXN**	0.67	0.68	0.69	0.52	0.61	0.62	0.61	0.74	-0.16	0.13
**RXP**	0.83	0.85	0.86	0.19	0.72	0.74	0.74	0.40	-0.67	-0.34
**RXQ**	0.81	0.82	0.83	0.30	0.78	0.80	0.81	0.29	-0.53	-0.53
**RXR**	0.97	0.96	0.97	0.88	0.82	0.84	0.84	0.69	-0.09	-0.15
**RXW**	0.75	0.77	0.78	0.18	0.76	0.79	0.80	0.46	-0.60	-0.34
**RYJ**	1.43	1.44	1.46	0.47	2.38	2.32	2.33	1.69	-0.99	-0.65
**RYQ**	1.50	1.53	1.55	0.48	1.61	1.67	1.68	0.80	-1.07	-0.88
**RYR**	0.79	0.80	0.80	0.47	0.81	0.80	0.80	0.69	-0.33	-0.11
**XXX**	0.00	0.00	0.00	0.00	0.00	0.00	0.00	0.00	0.00	0.00
**Total**	6,468,586	6,268,013	6,138,487	129,526	208,326	196,769	193,270	3,499		
**χ^2 test**	p < .001 (χ^2 = 6267610.79, df = 151)				p < .001 (χ^2 = 9154.95, df = 151)					

For peer review only - http://bmjopen.bmj.com/site/about/guidelines.xhtml

BMJ Open

Linkage of Maternity Hospital Episode Statistics Data to Birth Registration and Notification Records for Births in England 2005-2014: 2 - Quality Assurance of Linkage of Routine Data for Singleton and Multiple Births

Journal:	*BMJ Open*
Manuscript ID	bmjopen-2017-017898.R1
Article Type:	Research
Date Submitted by the Author:	31-Jul-2017
Complete List of Authors:	Harper, Gillian; City, University of London, School of Health Sciences
**Primary Subject Heading**:	Research methods
Secondary Subject Heading:	Health services research
Keywords:	Data linkage, Quality assurance, Births and maternities, Administrative data

SCHOLARONE™ Manuscripts

For peer review only

Linkage of Maternity Hospital Episode Statistics Data to Birth Registration and Notification Records for Births in England 2005-2014: 2 - Quality Assurance of Linkage of Routine Data for Singleton and Multiple Births

Author: Gill Harper, Research Fellow, City, University of London, London, United Kingdom

Correspondence address

City, University of London,
Centre for Maternal and Child Health Research,
1 Myddelton Street,
London. EC1R 1UW.
Tel: 0207 040 5315
Email: gill.harper@city.ac.uk

ORCID Number: 0000-0002-3492-2076

Disclaimer

It is essential that any copyright agreement I sign is in the form of a non-exclusive agreement as, under the terms of my current contract, the Crown has been assigned an irrevocable non-exclusive licence in respect of any information, Intellectual Property, Results, Materials and conclusions arising from the research project.

Funding

This work was supported by the National Institute for Health Research. HS&DR Programme, project number HS&DR 12/136/93. Project title: 'Births and their outcome: analysing the daily, weekly and yearly cycle and their implications for the NHS'

Word count: 6,858

Number of tables: 5

Competing interests: None

Author contribution

The author Gill Harper was responsible for the design of the quality assurance methodology, writing all parts of the paper, and giving final approval of the version to be published. I agree to be accountable for all aspects of the work in ensuring that questions related to the accuracy or integrity of any part of the work are appropriately investigated and resolved.

Data sharing statement

The author does not have permission to supply data or identifiable information to third parties, including other researchers but the research team of which she is a member has permission under Section 251 of the Health Service (Control of Patient Information) Regulations 2002 to analyse patient identifiable data for England and Wales without consent and create a research database which could be accessed by other researchers using the VML at the Office for National Statistics subject to permission from the Confidentiality Advisory Group of the Health Research Authority to access patient identifiable data without consent.

For peer review only

Keywords

Data linkage, Quality assurance, Administrative data, Births and maternities

Abstract

Objectives

To quality assure a Trusted Third Party linked dataset to prepare it for analysis.

Setting

Birth registration and notification records from the Office for National Statistics (ONS) for all births in England 2005-2014 linked to Maternity Hospital Episode Statistics (HES) delivery records by NHS Digital using mothers' identifiers.

Participants

All 6,676,912 births that occurred in England 1st January 2005 to 31st December 2014.

Primary and secondary outcome measures

Every link between a registered birth and a HES delivery record for the study period was categorised as either the same birth or a different birth to the same mother, or as a no-match, by comparing common baby data items and valid values in key fields with step-wise deterministic rules. Rates of preserved and discarded links were calculated and which features were more common in each group were assessed.

Results

98% of births originally linked to HES were left with one preserved link. The majority of discarded links were due to duplicate HES delivery records. Of the 4,854 discarded links categorised as no-match, clerical checks found 85% were false positives links, 13% were quality assurance false negatives, and 2% were undeterminable.

Births linked using a less reliable stage of the linkage algorithm, births at home and in the London region, and with birth weight or gestational age values missing in HES were more likely to have all links discarded.

Conclusions

Linkage error, data quality issues, and false negatives in the quality assurance procedure were uncovered. The procedure could be improved by allowing for transposition in date fields, and more discrimination between missing and differing values. The availability of identifiers in the datasets supported clerical checking. Other research using Trusted Third Party linkage should not assume the linked dataset is error-free or optimised for their analysis, and allow sufficient resources for this.

For peer review only - http://bmjopen.bmj.com/site/about/guidelines.xhtml

Strengths and Limitations

1. The study is the first time linked birth registration and hospital delivery records for ten years of data at the national level has been quality assured
2. A high linkage rate was maintained after quality assurance
3. The methodology was able to draw upon the availability of identifiers in both the ONS and HES data to inform the choice of rules at each stage in the methodology, to carry out clerical checks to ensure the accuracy of the results, and to explore data and linkage quality issues
4. Carrying the work out in the secure environment of ONS' VML safeguarded the data but limited software options and increased processing time
5. Any bias arising from non-submission of HES records by some trusts is likely to lead to bias in the extent to which records could be quality assured

For peer review only

Introduction

The quality assurance procedure described here was developed to support a project[1] for which data recorded at birth registration, birth notification and clinical information on maternity care at delivery were linked for analysis. After piloting in an earlier project,[2] the Office for National Statistics (ONS) mainstreamed the linkage of data recorded at birth registration in England and Wales to data recorded at birth notification, originally in an interim system, NHS Numbers for Babies (NN4B). These linked records of births from 2005 to 2014 were then linked to records from the Maternity Hospital Episode Statistics (HES) dataset for births that occurred in England and from the National Community Child Health Database /Patient Episode Database Wales (NCCHD / PEDW) for births that occurred in Wales. This linkage brought together key demographic and clinical data items unique to each dataset and not otherwise available together at a national level, enabling new analyses. For example, it made it possible to compare time of birth, recorded in birth notifications with birth outcomes such as mortality and conditions reported at birth, as ONS routinely links child death registration to birth registration records and reports. The further linkage with Maternity HES made it possible to analyses the outcomes of birth by onset of labour, mode of delivery, gestational age, time of day and day of the week. The history of the project and details of the linkage are described elsewhere[3].

The routinely linked ONS birth registration and birth notification records from here on will be referred to collectively as ONS birth records.

This paper describes quality assurance of the linked data for England, as data for Wales were linked separately.

Quality assuring this linkage aims to achieve two goals simultaneously. The first is to ensure that the ONS birth record has linked to the correct HES delivery record. As HES can contain more than one episode record for a delivery, the second aim of quality assurance is to select the most relevant HES delivery episode record with the most information from all the available correctly linked records.

Data sources

The ONS birth records were for 6,468,586 singleton and 208,326 multiple births in England from 1st January 2005 to 31st December 2014.

Only the Maternity HES delivery record which contains information on onset of labour and method of delivery was used. Maternity HES also contains a birth record for the baby, but linkage to these data and the further linkage to subsequent admissions of mothers and babies is not considered in this paper.

HES delivery records are usually grouped by financial year and were provided for the financial years 1st April 2004 to 31st March 2015 in order to include the calendar years used by ONS for its publications and analyses. They are for finished consultant episodes (FCEs) which capture information about diagnoses and procedures during the time a patient spends under the care of an individual consultant, or in the case of maternity, a midwife. Each delivery record contains an admitted patient care record with additional fields about the mother in an appended maternity tail, and information on the baby in an appended baby tail for up to nine babies.

Singleton and multiple birth deliveries are not explicitly identified in HES, but multiple delivery records can be identified where the diagnostic codes and the number of babies indicate a multiple

birth, and vice versa for singletons. On this basis, 7,040,590 HES delivery records relating to singleton births, and 123,497 relating to multiple births were identified. As stated above, the singleton and multiple births were quality assured separately. There can be more than one episode record per delivery, and some records are duplicated, therefore the number of HES delivery records is higher than the number of births.

Full details of the ONS and HES data are given elsewhere[3, 4] along with assessments of their data quality. [5-7] HES, particularly Maternity HES, has well recognised data quality problems[8] such as large numbers of missing data for some variables and missing maternity tails. The data recorded at birth registration is overseen by the General Register Office and processed by ONS for statistical purposes. ONS official statistics are considered as a 'gold' standard. Data quality has been found to influence the accuracy of linkage and create bias[2, 5-7, 9, 10]. Methods of cleaning HES data in isolation and by trust are discussed elsewhere.[11-14]

Linkage

Linkage of the ONS birth records to the Maternity HES delivery records was carried out by the Health and Social Care Information Centre, now known as NHS Digital, at the person-level, using maternal identifiers because HES delivery records are associated with mothers rather than their babies. It consists of a hierarchical step-wise linkage based on combinations of NHS Number, date of birth, sex, and postcode of the mother's place of residence (see Box 1). Every link is assigned the step of the algorithm that made the link, known as the match rank. On this basis, registered births to a mother during the study period 2005 to 2014 should link to all available delivery records for every birth that mother has had in those ten years.

Step/match rank	NHS number	DoB	Sex	Postcode	
1	Exact	Exact	Exact	Exact	
2	Exact	Exact	Exact		
3	Exact	Partial	Exact	Exact	
4	Exact	Partial	Exact		
5	Exact			Exact	
6		Exact	Exact	Exact	where NHSNO does not contradict the match and DOB is not 1 January and the POSTCODE is not in the 'ignore' list
7		Exact	Exact	Exact	where NHSNO does not contradict the match and DOB is not 1 January
8	Exact				

Box 1: NHS Digital hierarchical step-wise linkage algorithm used to link ONS birth records to HES delivery records

Mothers' identifiers from ONS birth records were linked first of all to the HES Patient Index to assign a HESID to each, then the HES delivery records associated with these HESIDs were extracted. Tables 1a and 1b give a summary of the linkage rates by calendar year, showing that 97% of singleton births and 95% of multiple births were linked to at least one HES delivery record.

For peer review only - http://bmjopen.bmj.com/site/about/guidelines.xhtml

Table 1a and 1b: Summary of linkage rates for singleton and multiple births by calendar year

Instances where the linkage algorithm did not link a registered birth to a HES delivery record could be because no delivery record existed, for example for home births and births in private health facilities which are not usually captured by HES, non-submission of HES data by particular trusts at particular times, or due to linkage error. Overall, 1.7% of singleton and 4.3% of multiple ONS birth records without a link to a HES delivery record did have a link to the mother's HESID. These problems have not been investigated further in this paper.

This paper focuses only on validating the records that were linked to a mother's HESID and HES delivery record by NHS Digital's algorithm. Singleton and multiple births were assessed separately due to the extra complexity associated with multiple birth registration records. This paper sets out the quality assurance procedures and results for both.

Methods

The aim was to assess the linked ONS birth and HES delivery records at the national level to efficiently identify one correctly linked HES record with the maximum amount of delivery information from all possible links to create a file to be used in analysis.

Both mothers' and babies' identifiers were available for the ONS birth and HES delivery records to use in the quality assurance process. These were mother's date of birth, NHS number and postcode of usual residence, and baby date of birth, NHS number and sex.

The work was carried out in the secure environment of ONS' Virtual Microdata Laboratory (VML). Microsoft Access database software was used as it was the only database software available in the VML at that time, and Structured Query Language (SQL) code was used for replicability. The relatively large size of the data files and restrictions on database size and processing speed constrained the design of the methods used.

The STROBE RECORD extension guidelines and checklist were followed where applicable.

Data preparation

It was necessary to normalise the baby information in the maternity tail section of each HES delivery record so that the data could be stored and interrogated efficiently in the database environment. This meant that if information was held on more than one baby in the maternity tail part of a delivery record, this was transformed into separate records for each baby. For example, this meant two records, one for each of the two babies in a twin delivery. Each record contained the same common episode identifier, admitted patient care fields, and maternity tail fields, but with the respective baby's information in each one.

For variables common to birth registration and birth notification, the maximum amount of reliable information was derived from the two sources. The baby's date of birth, birthweight and sex were taken from birth registration, and gestational age was taken from birth notification, unless values were missing.

Data types and formats, for example date fields and values for the baby's sex were made consistent between ONS and HES records.

Data quality

For peer review only - http://bmjopen.bmj.com/site/about/guidelines.xhtml

The extent of missing values for key items of data about the baby in the ONS birth records for 1st January 2005 to 31st December 2014 and HES normalised records for 1st April 2004 to 31st March 2015 is given in Table 2. A value would have to be missing on both birth registration and birth notification to be considered missing overall on ONS birth records in Table 2. While the total numbers may not be directly comparable due to definitional and date differences, the percentages of missing values per variable gives an indication of differences in data completeness.

Table 2: Comparison of percentages of missing values in key baby data items on ONS birth records and HES normalised delivery records for singleton and multiple births 2005 to 2014

The HES data have a much higher proportion of records with missing values than ONS birth records, for example up to 37% for gestational age in singleton births. The proportion of records with missing information is lower for multiple births in HES, as data items for the first baby are often repeated for their siblings in the same delivery.

Where the baby's date of birth was missing on HES, it was derived from operation dates if a delivery code was present. If sex of baby, gestational age or birthweight were missing, they could not be derived, however.

Gestational age and birthweight are known to have problems, notably implausible values, truncation and rounding in HES. ONS birth records can also have implausible values for birthweight but this has been improving with the introduction of 'warning' edits to birth notification systems.

Procedure

There are few examples of existing procedures to quality assure linked datasets. One example[15] cleaned and validated linked HES and mortality data for England by re-formatting and deriving variables, removing duplicates, and breaking erroneous links indicated where common data items were highly discordant.

A pilot was initially carried out on data for 2005 to help formulate the methodology. The following procedure is the final version that was carried out on all ten years of the linked data. Clerical checks were carried out throughout the development phase using relevant variables and identifiers to inform and improve the procedure.

On the assumption that the maternal linkage was correct, the links between the ONS birth records and HES delivery records were categorised as either 'same mother same birth' (SMSB) or 'same mother different birth' (SMDB). The category 'wrong birth' (WB) was for where neither of the previous categories apply, suggesting incorrect maternal linkage or data quality problems. These scenarios are summarised in Box 2. This excludes occasions when a birth registration record did not link to any HES delivery records.

An ONS birth record may link to one correct or incorrect HES delivery record, or to more than one HES delivery record with one, none or some of them being correct for any of the reasons in Box 2.

Category	Summary	Details	Action
Same mother same birth (SMSB)	ONS birth record linking to one relevant HES delivery record	One-to-one linkage as expected	Keep link
Same mother same birth (SMSB)	ONS birth record linking to many relevant HES delivery records, no clear episode order sequence	Due to duplicate HES records, or pre- and post-delivery HES records	Keep link to one of the duplicates or the most appropriate delivery record<br>Move others to unlinked file

For peer review only

Same mother same birth (SMSB)	ONS birth record linking to many relevant HES records, with a clear episode order sequence	Due to multiple episodes as part of a hospital spell	Keep link to one episode record and maximise delivery information<br>Move others to unlinked file
Same mother different birth (SMDB)	ONS birth record linking to one or more incorrect HES delivery record (same mother)	Another birth to the same mother within the study period	Move to unlinked file
Wrong birth (WB)	ONS birth record linking to one or more incorrect HES delivery record	The maternal linkage is incorrect or poor data quality	Move to unlinked file

Box 2: Categories of linked ONS birth records and HES delivery records

The quality assurance exercise aimed to keep 'same mother same birth' linked records (with the correct normalised record for multiple births), and move all others to the unlinked file. If more than one correct link was available, only one linked delivery record containing the maximum delivery information was chosen. This was to create a one-to-one relationship and support ease of analysis.

Baby data items were compared to ascertain if the linked records related to the same baby. These were baby's date of birth (derived date of birth in HES where applicable), sex, gestational age, birthweight, and location of birth.

The place of birth codes used differed between the datasets. These were translated into a consistent common location code, designed specifically for the study.[16]**Error! Bookmark not defined.** This accounted for NHS organisational changes which took place over the time period. Many maternity services continued to operate in the same location, while the NHS trusts responsible for them and, consequently their organisation code, changed over the study period. In addition, groups of location codes were identified that were within a reasonable geographical distance of each other that might transfer women between them, for example, if they developed problems which made it advisable to transfer to a hospital with a specialist neonatal unit. The presence of codes within these groups on the linked data could relate to the same birth.

Locations of births out-with an NHS trust, such as home births, were all treated as potential location matches because they could ultimately be transferred to an NHS hospital and have a HES delivery record, and the recording of the location for such births in HES can be unreliable. For these cases the other baby data items were relied on to establish if records were for the same baby.

Four combinations of these variables were used to confirm whether or not a linked ONS birth record and HES delivery record related to the same baby (see Box 3), in order of decreasing certainty from 1 to 4. Combination 1 required all the data items to match. Combination 2 required location and date of birth to match plus either birthweight or gestational age or sex. Combination 3 required only location and date of birth to match and birthweight, gestational age and sex will not match in these cases. Combinations 2 and 3 took into account the high occurrence of missing values and poor data quality in HES. Combination 4 accounted for a degree of data entry error in the date of birth by allowing it to differ by up to 4 days, but only if backed up by the location of birth matching, and birthweight and gestational age and sex not differing very much between ONS and HES if the values were not missing. If they were different and the date of birth was not an exact match, it was considered to be a different baby. The four combinations were applied in order so the most reliable matches are identified first.

For peer review only

Combination	ONS birth record and HES location of birth matches		ONS birth record and HES baby date of birth matches		ONS birth record and HES birthweight matches		ONS birth record and HES gestational age matches		ONS birth record and HES sex of baby matches
1	**Y**	**and**	**Y**	**and**	**Y**	**and**	**Y**	**and**	**Y**
2	**Y**	**and**	**Y**	**and**	**(Y**	**or**	**Y**	**or**	**Y)**
3	**Y**	**and**	**Y**						
4	**Y**	**and**	**Differs by up to 4 days***	**and**	**Missing or not completely different**	**and**	**Missing or not completely different**	**and**	**Missing or not completely different**

*by up to 10 days for multiple births

Box 3: The four combinations of baby data items that confirm that the link between the ONS birth record and HES relates to the same baby

If the linked records referred to the same baby and there was more than one linked HES delivery record to choose from, then the record with the maximum onset of delivery and delivery method information was chosen, as these variables were of key importance for the analysis. The presence of valid values in these and other fields were used to inform this.

The procedure was implemented using deterministic step-wise rules to be carried out in order, with any linked records dealt with at each stage excluded from subsequent stages. A summary is given in Box 4 and fuller details are given in Appendix A (Appendix A Table 1 for singleton births, and Appendix A Table 2 for multiple births). The complexity of quality assurance was greater for multiple births because each ONS birth record linked to all HES normalised records for that delivery through the mother's identifiers, by corresponding to each of the siblings. The procedure is slightly different for singleton and multiple births to account for this. For example, for multiple births the birth order is taken into consideration.

**Stage 1 - Cleaning**
Exclude links to invalid HES delivery records<br>Exclude links to surplus x 6 or x 9 duplicate HES delivery records
**Stage 2 - Same mother different birth**
Exclude links to HES delivery records where the HES baby date of birth is too far away from the ONS baby date of birth to be the same birth
**Stage 3 – Only one HES delivery record linked to an ONS birth record**
Keep if baby data items match<br>Exclude if baby data items do not match
**Stage 4 – Multiple HES delivery records all in same spell linked to an ONS birth record**
Keep episode if baby data items match and contains maximum delivery information<br>Exclude episode(s) if baby data items do not match and/or episode contains less delivery information
**Stage 5 – Multiple HES delivery records containing duplicate information linked to an ONS birth record**
Keep record if baby data items match with latest episode dates<br>Exclude record(s) if baby data items do not match and/or earlier episode dates
**Stage 6 - Multiple HES delivery records containing different information linked to an ONS birth record**
Keep record if baby data items match and contains maximum delivery information<br>Exclude record(s) if baby data items do not match and/or contains less delivery information

Box 4: Summary of quality assurance procedure

The first two rules cleaned the HES data by eliminating invalid linked records and duplicates. It is known that some trusts generate multiple copies of the same delivery episode record, usually six or

For peer review only

nine copies of the same record. In these cases only one was kept to be quality assured and the others were discarded for efficiency.

Checks

Once all rules in the procedure had been carried out, it was confirmed that all linkages had been assigned a decision, and there were no more records to check.

Further checks were made on:

1. Private hospital births should not have a link to a HES delivery record unless the mother or baby were later transferred to an NHS hospital for any reason
2. Each HES delivery record should link to the correct number of ONS birth records depending if it is for a singleton or multiple delivery
3. The ONS birth record baby date of birth fell within the HES delivery admission period (no admission dates for home births). This was not always the case even when it was a confident link
4. If there was more than one correct and relevant linked HES delivery record for an ONS birth record, any missing onset of delivery and method of delivery information was filled in from these

Results

The quality assurance procedure took the equivalent of 55 working days for singleton births and 16 days for multiple births. The database software and processing limitations of the VML at that time slowed the procedure down. SQL Server has since been made available in VML and would speed this up considerably.

Seventeen per cent of singleton births linked records were dealt with in the first two cleaning stages, and a further 80% were dealt with by rules 3 and 4. All the remaining rules were required to deal with the residual 3% of more complex cases.

Five per cent of multiple births linked records were dealt with in the first two cleaning stages, and a further 60% were dealt with by rules 3 and 4. This left 35% as residual records, reflecting the greater complexity in multiple birth records.

For each year, the complex residual cases were clustered within particular trusts. This was probably due to IT problems and changes to systems.

A summary of the results for singleton and multiple births is given in Tables 3a) and 3b), broken down by calendar year.

Table 3a: Summary of quality assurance results for singleton births by calendar year (QA = quality assurance)
Table 3b: Summary of quality assurance results for multiple births by calendar year (QA = quality assurance)

Of the total 6,468,586 singleton ONS birth records, 97% had been linked to one or more HES delivery records and could be quality assured. Of these, 98% remained linked to one HES delivery record and 2% had all HES links discarded after quality assurance. Ninety five per cent of all ONS singleton birth records overall were left with a link to a HES record for analysis.

For peer review only

Of the 208,326 multiple births, 95% were linked to one or more HES delivery records and could be quality assured. Of these, 98% remained linked to one HES delivery record and 2% had all HES links discarded after quality assurance. Ninety three per cent of all ONS multiple birth records overall were left with a link to a HES record for analysis.

For singleton births, the percentage of all birth records left linked to one HES delivery record after quality assurance increased more or less year on year from 93% in 2005 to 95% in 2014. For multiple births, the rate increased from 94% in 2005 to 97% on 2010, but then started to decrease again to 87% in 2014. This reflects the same patterns as the percentage of ONS birth records that were originally linked to a HES delivery record and are biased by the number of records that linked to a HESID but no delivery record that were excluded from quality assurance.

Reasons for discarding HES records linked to an ONS birth

After quality assurance, all or some of the available linked HES records will have been discarded, leaving only a link to one correct HES record, or to none at all. Discarding all the linked HES records for a birth suggests incorrect linkage and is discussed in the next section. Discarding all but one is due to a variety of reasons why there may be more than one HES record available for a birth.

Seventy six per cent of all the linked HES records that were discarded were due to duplicate copies of HES delivery episodes, and 0.5% were due to invalid delivery records, dealt with in the cleaning stages 1 and 2 of the methodology. A summary of the remaining reasons by year for singleton births is given in Table 4.

Table 4: Breakdown of reasons for discarding HES delivery records linked to an ONS birth excluding duplicate copies of HES delivery episodes and invalid deliveries for England singleton births by year

'Same mother different births' (column a) accounted for 60% of all discarded links. This is expected when the study period covers ten years and each birth is linked by the mothers' identifiers to all other deliveries to that mother in that period. The second main reason which accounted for 21% of discarded links, was when there were additional episodes relating to a delivery, usually a pre- or post-delivery admission (column b). The numbers of these generally decreased over time, suggesting data quality is a factor. The third main reason, accounting for 16%, was when links to other episodes in a spell have been discarded, keeping only the link to the episode with the most amount of delivery information (column c).

The remaining three reasons each accounted for fewer than 2% of discarded links. Column d is when multiple delivery records contained the same information and links to superfluous records were discarded. This is also a data quality issue. There was a large increase in these in 2014. Column f relates to a small number of linked singleton birth records that were identified as 'potential multiple births', described later in this paper.

Column e are 'no matches'; links discarded where there are no matching variables or insufficient numbers of matching variables according to the quality assurance methodology, and the baby's date of birth did not indicate a 'same mother different birth'. This group is of interest because it could be indicating erroneous false positive links by the linkage algorithm, or it could indicate erroneous false negatives in the quality assurance methodology.

Fifty seven per cent (2,743) of this group were linked by match rank 1 of the linkage algorithm, the most reliable stage of the linkage algorithm that matches all available identifiers including the NHS

For peer review only - http://bmjopen.bmj.com/site/about/guidelines.xhtml

number, suggesting they should be correct maternal linkages. These were checked in greater detail clerically for explanations.

Of these, 2,323 (85%) of these were true no matches where either none of the five baby data items matched at all, or only the place of birth matched, or only sex matched, or only gestation matched. The baby's date of birth has not been transposed nor were 'same mother different birth', suggesting incorrect maternal linkage or some other data quality issue. This is 0.04% of all singleton birth records originally linked to a HES delivery record.

It appeared that 366 (13%) were potential false negatives made in error by the quality assurance methodology. The dates of birth in these do not match due to transposition in the date of birth elements. If this had been factored into the methodology, these links would have not been discarded.

The remaining 94 (3%) were undeterminable, with some discriminatory baby data items matching and some not. For example, the date of birth may match or have been transposed, but birthweight and/or place of birth may be very different.

Some of the categories of problem shown in Table 4 may have been restricted to particular trusts but further investigation would be needed to check this.

Differences between records with correct and incorrect linkage

Checks were carried out to ascertain if the ONS birth records that remained correctly linked to one HES delivery record after the quality assurance procedure, and the ONS birth records with all links to HES discarded after the quality assurance procedure differed significantly by key variables. Discarding all links could indicate linkage error, data quality issues, or false negatives in the quality assurance methodology.

Appendix B gives tables to compare the proportions of values for the key variables for both groups, and chi-square tests results for testing the null hypothesis that the distribution of a variable is the same in the two groups. Variables from the ONS birth record include the match rank of the linkage algorithm, the region of birth, the year of birth, the month of birth, the day of birth (8) based on an 8 day categorisation including public holidays, the day of birth (11) based on an 11 day categorisation including specific public holidays and the days before and after them, the hour of birth, the age of the mother, the sex of the baby, the ethnicity of the baby, gestational age, if a stillbirth, and the location of birth. Missing gestational age and missing birthweight in HES were also included.

The null hypothesis is rejected for all variables except for sex, day (8), and day (11) in multiple births. Finding significant differences is to be expected because the dataset is large and the chi-square test can pick up subtle differences, but this does not tell us very much. Comparing the difference in the percentage distribution of values for each variable for the two groups provides more information on how they differ. This is given in Table 5 for singleton and multiple births where the difference is greater than 1%.

Table 5: Comparing the difference in the percentage distribution of values for key variables for ONS birth records that remained correctly linked to one HES delivery record after the quality assurance procedure, and the ONS birth records with all links to HES discarded after the quality assurance, for singleton and multiple births

These differences give an indication of when incorrect linkage is more likely to occur, or indeed false negatives in the quality assurance. Differences between singleton and multiple births are seen. Some

For peer review only - http://bmjopen.bmj.com/site/about/guidelines.xhtml

of these associations with incorrect linkage make sense. Home births are less likely to have a HES delivery record as many are not captured by hospital-based clinical systems from which HES records are derived. Match rank 6 is a less reliable stage of the linkage algorithm because it does not match on the NHS number. HES data quality is previously known to be poorer in years 2006 and 2007, although for multiple births, years 2012 to 2014 are more likely to be incorrectly linked. Missing data for gestational age and birthweight in HES values means there is less information to make a correct link. The other factors require more investigation or could be simply due to chance with the large numbers involved.

Table B.1 in Appendix B breaks down the proportions of each group by the match rank of the NHS Digital linkage algorithm, providing useful information about the quality of their linkage at each stage.

Data and linkage quality issues

Quality assurance of the linked dataset for such a large population revealed issues with the quality of the data within each of the datasets and also the quality of linkage that otherwise might not be detectable. While it is already known that HES has data quality issues, issues were found in the ONS birth records that warranted further investigation.

Firstly, the consistent common location code that was assigned to the place of birth codes was found to not match between birth registration and notification for 22% of the singleton births with a correct HES link after quality assurance. This suggested the linkage between birth registration and notification could be incorrect. A sample of these records that also had very discordant mother and baby identifiers and baby tail information was given to ONS, which carried out this linkage, for checking.

ONS confirmed that the baby NHS number or the sequence number[17] matched for these cases and therefore the linkage was correct between ONS birth registration and birth notification, regardless of the discordant variables. However, no quality assessment of the sequence number assignment process was available, so 542 of the most suspect cases were flagged. It was decided to exclude these from analyses.

Secondly, a small number of singleton birth records appeared to be multiple birth records (see Table 4 column f) based on a combination of HES variables suggesting a multiple birth delivery, and other birth registrations existing to the same mother on the same day, yet these were not labelled as a multiple birth by ONS. Again, these cases were checked by ONS which confirmed that they should be multiple births. The error was explained by a delay in some records being registered and received, possibly causing issues with finding the corresponding record in its system.

Lastly, after quality assurance, a small number of multiple births did not have all the expected number of ONS birth records, for example, a twin birth only had one ONS birth record associated with it rather than two. These were not always explained by stillbirths prior to 24 weeks gestation which should not be registered as a birth. Investigation revealed that the missing ONS birth records could often be found in the ‘unlinked’ ONS birth records file, to which no HES records were linked. This could be the case even if the mothers’ identifiers, including NHS number, clearly matched.

This was discussed with our contact at NHS Digital who investigated this. It was explained that these missed links were due either to the HES patient index having been updated since the linkage was done originally, or linkage error. The project did not attempt to find any further missing links.

For peer review only - http://bmjopen.bmj.com/site/about/guidelines.xhtml

The advantage of having the identifiers available to the project team for both ONS birth and HES delivery records made it possible to investigate and explain the above issues, but other research projects may not. Overall, this highlights how researchers and analysts should not assume that any routinely collected administrative datasets and linkages carried out between them are error-free.

Discussion

Main findings

This paper describes the quality assurance process that was carried out on ten years of linked ONS birth records and Maternity HES delivery records at a national level using patient identifiers. The aim was to prepare the linked dataset for analysis but the results also shed light on the quality of the data and the linkage itself. This makes it informative for other users of similarly linked data and for NHS Digital staff doing the linkage.

The method adopted compares common baby data items, giving most importance to matching babies' date of birth and location of birth. Altogether, 95% of all singleton births and 93% of all multiple births in England in the period 2005 to 2014 were linked to one correct HES delivery record after quality assurance.

Administrative duplicate HES records were found to be the main reason for discarding links. Other reasons were due to other births to the same mother within the study period, the presence of multiple HES records per delivery for various reasons, and error in the linkage and in the quality assurance method itself.

Certain features were more common in incorrectly linked records than correctly linked records. These included births linked using match rank 6 of the linkage algorithm, births at home and in the London region, and births where the birthweight or gestational age was missing in HES. This suggests some bias in the linkage and often relates to data quality issues. These patterns differed between singleton and multiple births.

Missed links were also discovered, which together with the previous findings provides further evidence on the quality of the linkage.

It was possible to identify aspects of the quality of the ONS birth records data and have ONS investigate them.

Strengths and limitations, and future work

The quality assurance procedure was able to draw upon the availability of identifiers in both the ONS and HES data to inform the choice of rules at each stage in the methodology, to carry out clerical checks to ensure the accuracy of the results, and to explore data and linkage quality issues. The procedure successfully identified correct and incorrect linkages to prepare the large linked dataset for analysis within the resources available.

Because personally identifiable data was used, the work was carried out in the secure environment of ONS' VML. While security was safeguarded, this led to many delays and disruption due to software and processing limitations of systems and servers. This was costly in terms of researcher time and has limited the extent of analytical outputs from the study.

Quality assurance could only be carried out on ONS birth records that had linked to a HES delivery record. Links where a HESID was found but no delivery record was available were excluded. If the

For peer review only

main reason for this is because some trusts did not submit the HES delivery record, then bias will be inherently present in which linked records could be quality assured and which couldn't.

The methodology to decide if the linked records related to the same baby was purely deterministic and did not allow for transposition of date of birth elements, or for rounding and truncating in the birthweight and gestational age values. It did not strongly discriminate between when values were missing rather than different.

A disproportionate amount of effort was spent on the approximately 4% of complex linked records that remained after stages 1-4 of the methodology.

Future work could address these issues by incorporating more flexible matching criteria and more thoroughly preparing the birthweight and gestational age variables for data entry errors beforehand. Ways to simplify the methodology to fewer variable combinations or rules to identify correct and incorrect links could be explored. Future work could also assess how variation in data quality at trust level affects linkage quality and the results.

Implications

The linkage error identified by quality assuring the linked dataset suggests that linkage carried out using similar algorithms and by 'Trusted Third Parties' generally, cannot be assumed to be error-free and may affect any analyses carried out on them. This has implications for other research projects that are not informed of the quality of the linkage, or have the means to quality assure it themselves using identifiers. In particular, no assessment of the quality of the linkage algorithm is routinely published by NHS Digital.

A very similar linkage algorithm is used to routinely link HES data to ONS death registration data, differing only at match rank 8,[18] although different approaches were used earlier by the National Centre for Health Outcomes Development[19] and the Department of Health. This linked data is used to analyse outcomes of hospital care such as immediate and long-term survival rates and hospital-based mortality indices including the Standard Hospital Mortality Index (SHMI) and indices of 30-day mortality. Concerns over how quality of this linkage can affect analyses using the 30-day mortality indicator have been reported.[20] On the other hand, an evaluation of the mortality linkage for SHMI purposes found that 'the proportion of erroneous linkages is sufficiently small to not impact on a SHMI result', but its report did not explicitly describe the linkage algorithm used.[21] The relevant member of its study team has confirmed that the team did not know what linkage algorithm was used by the then Information Centre for Health and Social Care to link the mortality data for SHMI purposes, and they were not provided with NHS numbers or other identifiable variables to enable performing the linkage themselves or indeed to investigate the quality of the linkage (Fotheringham J, personal communication).

Linkage of identifiable data for research purposes is mostly carried out by 'Trusted Third Parties' such as NHS Digital. The Trusted Third Party model replaces identifiers with an encrypted code, and only the de-identified data are made available to researchers in a secure environment. The Digital Economy Bill[22] announced in May 2016, sought increased access to de-identified data for research, meaning that reliance on Trusted Third Party linkage would increase. The Bill implies that data accessed under the new legislation must only be linked using the Trusted Third Party model. However, responses to this and to The Cabinet Office consultation document 'Better Use of Data in Government'[23] from bodies such as the Royal Statistical Society[24] and the Health Statistics User Group[24] have expressed concern over this and also that the proposed legislation does not include health and social care data. Elements of the Digital Economy Bill could be interpreted as allowing

For peer review only

flexibility in who can access and link identifiable data so long as disclosure is avoided and other conditions and codes of practice are in place. Clarification on this is required so that it can be established if researchers will be restricted only to pre-linked data without any means to assess their quality.

In the context of the use of linked data for statistics, the ONS has expressed a preference to be able to link data with identifiers rather than encryption to achieve better quality official population statistics in the future[25].

Conclusion

Carrying out quality assurance of linked records has informed knowledge of both the quality of the data and of the linkage, providing benefits over cleaning each dataset in isolation. Other research projects using large linked routinely collected datasets should not assume the linked datasets are error-free or optimised for their analysis. This has implications especially for users of anonymised linked data. The resources needed to do quality assurance and prepare effectively prior to generating descriptive or analytic reports are considerable and need to be addressed when planning and costing large data linkage projects.

For peer review only

Acknowledgement

The author would like to thank the members of the project team and its Study Advisory Group for their help and advice. In particular Nirupa Dattani who managed the linkage, Rod Gibson who set up the database, and Alison Macfarlane as principal investigator. And also to thank all the relevant colleagues in the Office for National Statistics and NHS Digital, formerly the Health and Social Care Information Centre for their help. In particular, to thank Emma Gordon, Joanne Evans, Claudia Wells, Alex Lloyd, Justine Pooley, Elizabeth Mclaren and members of the VML Team at the Office for National Statistics, and Ariane Alamdari and Garry Coleman at NHS Digital.

Disclaimers

The views and opinions expressed here are those of the author and do not necessarily reflect those of the HS&DR programme, NIHR, NHS or the Department of Health.

The data were processed in the secure environment of the Office for National Statistics' Virtual Microdata Laboratory and the following disclaimer applies:

This work contains statistical data from ONS which is Crown Copyright. The use of the ONS statistical data in this work does not imply the endorsement of the ONS in relation to the interpretation or analysis of the statistical data. This work uses research datasets which may not exactly reproduce National Statistics aggregates.

Approvals

Ethics approval 05/Q0603/108 and subsequent substantial amendments were granted by East London and City Local Research Ethics Committee 1 and its successors.

Permission to use patient identifiable data without consent under Section 60 of the Health and Social Care Act 2001 was initially granted by the Patient Information Advisory Group PIAG 2-10(g)/2005. Renewals and amendments and a second permission CAG 9-08(b)2014 under Section 251 of the Health Service (Control of Patient Information) Regulations 2002 were granted by its successor bodies, the Ethics and Confidentiality Committee of the National Information Governance Board and the Confidentiality Advisory Group of the Health Research Authority.

A second permission CAG 9-08(b)2014 to use patient identifiable data without consent under Section 251 of the Health Service (Control of Patient Information) Regulations 2002 and create a research database held at the Office for National Statistics for analyses relating to inequalities in the outcome of pregnancy and to inform maternity service users about the outcome of midwifery, obstetric and neonatal care was granted by the Confidentiality Advisory Group of the Health Research Authority.

Permission to access data from the Office for National Statistics in the VML was granted by ONS' Microdata Release Panel. All members of the research team successfully applied for ONS Approved Researcher Status.

Permission to link and analyse data held by the Health and Social Care Information Centre, now NHS Digital was granted under Data Sharing Agreement NIC-273840-N0N0N.

Copyright

It is essential that any copyright agreement I sign is in the form of a non-exclusive agreement as, under the terms of my current contract, the Crown has been assigned an irrevocable non-exclusive

For peer review only

licence in respect of any information, Intellectual Property, Results, Materials and conclusions arising from the research project.

For peer review only

References

1. 'Births and their outcome: analysing the daily, weekly and yearly cycle and their implications for the NHS' funded by the National Institute for Health Research. HS&DR Programme, project number HS&DR 12/136/93.
2. Hilder L, Moser K, Dattani N, Macfarlane A. Pilot linkage of NHS Numbers for Babies data with birth registrations. Health Statistics Quarterly. 2007(33):25-33.
3. Dattani N. Linkage of Maternity Hospital Episode Statistics data to birth registration and NHS Numbers for babies records, England 2005-2014: methods. Forthcoming.
4. Ghosh RE, Ashworth DC, Hansell AL, Garwood K, Elliott P, Toledano MB. Routinely collected English birth data sets: comparisons and recommendations for reproductive epidemiology. Arch Dis Child Fetal Neonatal Ed. 2016;101(5):F451-7.
5. Hockley C, Quigley M, Johnson J, Rosenberg R, Dezateux C, Joshi H. Millennium Cohort Study: Birth Registration and Hospital Episode Statistics Linkage. A Guide to the Dataset. Centre for Longitudinal Studies Bedford Group for Lifecourse & Statistical Studies Institute of Education, University of London, 2007.
6. Dattani N, Datta-Nemdharry P, Macfarlane A. Linking maternity data for England, 2005-06: methods and data quality. Health Stat Q. 2011(49):53-79.
7. Dattani N, Datta-Nemdharry P, Macfarlane AJ. Linking maternity data for England 2007: methods and data quality. Health Statistics Quarterly. 2012;53:4-21.
8. HES Data Quality: NHS Digital. Available from: http://content.digital.nhs.uk/article/1825/The-processing-cycle-and-HES-data-quality.
9. Hagger-Johnson G, Harron K, Fleming T, Gilbert R, Goldstein H, Landy R, et al. Data linkage errors in hospital administrative data when applying a pseudonymisation algorithm to paediatric intensive care records. BMJ Open. 2015;5.
10. Knight HE, Gurol-Urganci I, Mahmood TA, Templeton A, Richmond D, van der Meulen JH, et al. Evaluating maternity care using national administrative health datasets: how are statistics affected by the quality of data on method of delivery? BMC Health Serv Res. 2013;13:200.
11. Raleigh VS, Foot C. Getting the Measure of Quality: Opportunities and Challenges. London: 2010.
12. Sinha S, Peach G, Poloniecki JD, Thompson MM, Holt PJ. Studies using English administrative data (Hospital Episode Statistics) to assess health-care outcomes—systematic review and recommendations for reporting. Eur J Public Health. 2013;23(1):86-92.
13. Royal College of Obstetricians and Gynaecologists. Patterns of Maternity Care in English NHS Hospitals 2011/12. London: 2013.
14. Sandall J, Murrells T, Dodwell M, al e. The efficient use of the maternity workforce and the implications for safety and quality in maternity care: a population-based, cross-sectional study. Health Services and Delivery Research. 2014;2(38).
15. RCPCH Child Health Reviews – UK Clinical Outcome Review Programme. Overview of child deaths in the four UK countries. September 2013.
16. Dodwell M. Assigning pseudo trusts to birth locations. Forthcoming.
17. Pregnancy and ethnic factors influencing births and infant mortality: 2013: ONS. Available from: https://www.ons.gov.uk/peoplepopulationandcommunity/healthandsocialcare/causesofdeath/bulletins/pregnancyandethnicfactorsinfluencingbirthsandinfantmortality/2015-10-14#data-sources-and-linkage.
18. Guide to linked mortality data from HES and the Office for National Statistics: NHS Digital. Available from: http://content.digital.nhs.uk/media/11668/HES-ONS-Mortality-Data-Guide/pdf/mortality_guide.pdf.
19. Gill LE, Goldacre MJ. English National Record Linkage of Hospital Episode Statistics and Death Registration Records. Report to the Department of Health, 2003. National Centre for Health Outcomes Development, Unit of Health-Care Epidemiology, University of Oxford, 2003.

For peer review only

20. Cooper Z, Gibbons S, Jones S, McGuire A. Does hospital competition save lives? Evidence from the NHS patient choice reforms. Economic Journal. 2011;121:228-60.
21. Campbell MJ, Jacques RM, Fotheringham J, Pearson T, Maheswaran R, Nicholl J. An evaluation of the Summary Hospital Mortality Index. Final report. Sheffield: ScHARR, The University of Sheffield, 2011.
22. Digital Economy Bill: House of Commons. Available from: https://www.publications.parliament.uk/pa/bills/cbill/2016-2017/0045/cbill_2016-20170045_en_1.htm.
23. Better use of data in government: Cabinet Office. Available from: https://www.gov.uk/government/consultations/better-use-of-data-in-government.
24. Statement on the Cabinet Office consultation 'Better use of data in government': Royal Statistical Society; 1 April 2016. Available from: http://www.rss.org.uk/Images/PDF/influencing-change/2016/RSS-Statement-on-Better-Use-of-Data-consultation-April-2016.pdf.
25. Methodology of Statistical Population Dataset V2.0: ONS. Available from: https://www.ons.gov.uk/census/censustransformationprogramme/administrativedatacensusproject/methodology/methodologyofstatisticalpopulationdatasetv20#summary-and-future-developments.

For peer review only

Tables

Table 1a: Summary of linkage rates for singleton births by calendar year

Year	Total singleton ONS birth records	Total singleton ONS births linked to any Maternity HES by NHS Digital	Total singleton ONS births not linked to any Maternity HES by NHS Digital	Singleton ONS birth records linked to HESID but no delivery record	% Total singleton ONS births linked to any Maternity HES by NHS Digital
2005	599,237	565,559	33,678	16,349	94.4
2006	620,730	589,127	31,603	15,802	94.9
2007	638,995	612,782	26,213	12,889	95.9
2008	656,196	635,411	20,785	9,908	96.8
2009	653,322	636,284	17,038	9,099	97.4
2010	665,599	652,533	13,066	7,009	98.0
2011	666,582	653,552	13,030	8,329	98.0
2012	676,399	661,511	14,888	11,444	97.8
2013	647,666	633,222	14,444	11,913	97.8
2014	643,860	628,032	15,828	13,514	97.5
**Total**	**6,468,586**	**6,268,013**	**200,573**	**116,256**	**96.9**

Table 1b: Summary of linkage rates for multiple births by calendar year

Year	Total multiple ONS birth records	Total multiple ONS births linked to any Maternity HES by NHS Digital	Total multiple ONS births not linked to any Maternity HES by NHS Digital	Multiple ONS birth records linked to HESID but no delivery record	% Total multiple ONS births linked to any Maternity HES by NHS Digital
2005	18,376	17,404	972	510	94.7
2006	19,541	18,522	1,019	602	94.8
2007	20,066	19,257	809	450	96.0
2008	20,803	20,100	703	378	96.6
2009	22,008	21,338	670	369	97.0
2010	21,501	21,033	468	276	97.8
2011	22,099	21,199	900	722	95.9
2012	22,058	20,166	1,892	1,805	91.4
2013	20,767	18,735	2,032	1,917	90.2
2014	21,107	19,015	2,092	2,009	90.1
**Total**	**208,326**	**196,769**	**11,557**	**9,038**	**94.5**

Table 2: Comparison of %s of missing values in key baby data items on ONS birth records and HES normalised delivery records for singleton and multiple births 2005 to 2014

For peer review only

Variable	ONS singleton birth records	HES singleton delivery records	ONS multiple birth records	HES multiple delivery records
Total	6,468,586	7,040,590	208,326	230,019
% with missing values for:				
Baby's date of birth	0.00	19.4	0.00	16.3
Sex of baby	0.00	23.1	0.00	16.1
Gestational age	0.71	37.2	0.80	27.8
Birthweight	0.56	22.7	1.70	15.3

For peer review only

Table 3a: Summary of quality assurance results for singleton births by calendar year (QA = quality assurance)

Year	Total singleton ONS birth records	Total singleton ONS births linked to any Maternity HES by NHS Digital	% Total singleton ONS births linked to any Maternity HES by NHS Digital	Of singleton ONS births linked to any Maternity HES by NHS Digital, total left with one link to HES after QA	Of singleton ONS births linked to any Maternity HES by NHS Digital, % left with one link to HES after QA	Of singleton ONS births linked to any Maternity HES by NHS Digital, total left with no link to HES after QA	Of singleton ONS births linked to any Maternity HES by NHS Digital, % left with no link to HES after QA	% of total singleton ONS births left with one link to HES after QA	% of total singleton ONS births left with no link to HES after QA
2005	599,237	565,559	94.4	554,566	98.1	10,993	1.9	92.5	7.5
2006	620,730	589,127	94.9	573,770	97.4	15,357	2.6	92.4	7.6
2007	638,995	612,782	95.9	595,585	97.2	17,197	2.8	93.2	6.8
2008	656,196	635,411	96.8	621,006	97.7	14,405	2.3	94.6	5.4
2009	653,322	636,284	97.4	621,423	97.7	14,861	2.3	95.1	4.9
2010	665,599	652,533	98.0	641,167	98.3	11,366	1.7	96.3	3.7
2011	666,582	653,552	98.0	642,263	98.3	11,289	1.7	96.4	3.6
2012	676,399	661,511	97.8	648,501	98.0	13,010	2.0	95.9	4.1
2013	647,666	633,222	97.8	622,943	98.4	10,279	1.6	96.2	3.8
2014	643,860	628,032	97.5	617,263	98.3	10,769	1.7	95.9	4.1
Total	6,468,586	6,268,013	96.9	6,138,487	97.9	129,526	2.1	94.9	5.1

For peer review only

Table 3b: Summary of quality assurance results for multiple births by calendar year (QA = quality assurance)

Year	Number of multiple ONS birth records	Number of multiple ONS births linked to any Maternity HES by NHS Digital	Percentage of multiple ONS births linked to any Maternity HES by NHS Digital	Number left with one link to HES after QA	Percentage left with one link to HES after QA	Number left with no link to HES after QA	Percentage left with no link to HES after QA	Percentage of all multiple ONS births left with one link to HES after QA	Percentage of all multiple ONS births left with no link to HES after QA
2005	18,376	17,404	94.7	17,209	98.9	195	1.1	93.6	1.1
2006	19,541	18,522	94.8	18,258	98.6	264	1.4	93.4	1.4
2007	20,066	19,257	96.0	18,957	98.4	300	1.6	94.5	1.5
2008	20,803	20,100	96.6	19,894	99.0	206	1.0	95.6	1.0
2009	22,008	21,338	97.0	21,085	98.8	253	1.2	95.8	1.1
2010	21,501	21,033	97.8	20,847	99.1	186	0.9	97.0	0.9
2011	22,099	21,199	95.9	20,872	98.5	327	1.5	94.4	1.5
2012	22,058	20,166	91.4	19,554	97.0	612	3.0	88.6	2.8
2013	20,767	18,735	90.2	18,165	97.0	570	3.0	87.5	2.7
2014	21,107	19,015	90.1	18,429	96.9	586	3.1	87.3	2.8
**All years**	**208,326**	**196,769**	**94.5**	**193,270**	**98.2**	**3,499**	**1.8**	**92.8**	**1.7**

For peer review only

Table 4: Breakdown of reasons for discarding HES delivery records linked to an ONS birth excluding duplicate copies of HES delivery episodes and invalid deliveries for England singleton births by year

Year	All<br>Total links broken (excluding exact duplicates and invalid records)	a<br>Same mother different birth	b<br>Multiple episodes - pre/post delivery admission	c<br>Multiple episodes - part of spell	d<br>Multiple episodes - exact duplicates with different epikeys	e<br>No match	f<br>Potential multiple birth
2005	46,164	24,457	14,941	5,805	104	817	40
2006	49,706	25,082	16,565	6,984	204	782	89
2007	48,999	26,304	13,187	8,301	323	799	85
2008	47,748	26,032	9,466	11,473	89	614	74
2009	40,519	25,688	6,137	7,680	283	673	58
2010	37,007	24,993	5,176	5,819	366	599	54
2011	37,013	24,679	6,033	5,367	741	145	48
2012	36,176	24,966	5,899	4,298	838	146	29
2013	34,285	23,071	5,709	5,018	281	169	37
2014	31,690	21,980	3,662	3,957	1,863	110	118
**All years**	**409,307**	**247,252**	**86,775**	**64,702**	**5,092**	**4,854**	**632**
Percentage	***100***	***60.41***	***21.2***	***15.81***	***1.24***	***1.19***	***0.15***

For peer review only - http://bmjopen.bmj.com/site/about/guidelines.xhtml

Table 5: Comparison of differences in the percentage distributions of values for key variables for ONS birth records that remained correctly linked to one HES delivery record after the quality assurance procedure, and the ONS birth records with all links to HES discarded after the quality assurance, for singleton and multiple births

	Singleton births		Multiple births	
**Variable**	**Value**	**Difference in percentages***	**Value**	**Difference in percentages***
Match rank of linkage algorithm	6	5.4		
Region of birth	Elsewhere	2.3	East of England	1.9
	Home	38.3	London	5.8
			Home	2.8
Year of birth	2006	2.5	2012	7.4
	2007	3.6	2013	6.9
	2009	1.4	2014	7.2
Month of birth	March	2.0	March	1.7
			April	1.5
Day of birth (8)			Saturday	1.2
Day of birth (11)			Saturday	1.2
Hour of birth	3.00 - 5.59	1.7	6.00 - 8.59	1.4
	6.00 - 8.59	1.8	9.00 -11.59	1.5
Sex of baby			Male	1.1
Age of mother	30-34	1.7	30-34	1.2
	35-39	1.3	35-39	2.4
			40-44	3.9
Ethnicity of baby	Not known	3.4	Black African	1.9
			Not known	1.4
Gestational age	Pre-term	1.1	Pre-term	1.1
Stillbirth	Yes	2.3	Yes	2.5
Gestational age missing on HES	Yes	20.1	Yes	10.9

For peer review only

For peer review only - http://bmjopen.bmj.com/site/about/guidelines.xhtml

Birthweight missing on HES	Yes	12.3	Yes	7.0
Trust	PPL (Portland)	1.7	PPL (Portland)	2.5
	RGQ (Ipswich)	2.0	RGQ (Ipswich)	1.5
	RKE (Whittington)	4.0	RJ6 (Croydon)	1.3
	RVL (Barnet and Chase Farm)	1.4	RKE (Whittington)	3.9
			RQ8 (Mid Essex)	1.6
			RTH (Oxford University Hospitals)	1.6
			RVL (Barnet and Chase Farm)	1.4
			RVV (East Kent)	2.0
			RW3 (Central Manchester)	1.1
			RWH (East and North Hertfordshire)	1.1

* Over one per cent

For peer review only

Appendix A

Quality assurance procedures for singleton and multiple births to select only one correct linked HES record for each ONS birth record and discard incorrect linkages (detailed version)

1: Singleton births

RULE	CATEGORY	APPLIES TO	K (Keep the link)	D (Discard the link)	NOTES
1	Cleaning	Linked to HES records that have no birth or op dates or is an invalid delivery		HES records where baby date of birth = null or valid delivery = false	
2	Cleaning	Duplicate HES records (typically x 6 or x 9) linked to same ONS birth record where baby tail information and identifier is identical	The first version of the duplicate HES record goes through to the subsequent cleaning stages	All other versions of the duplicate HES record	Due to IT administrative issue with some NHS Trusts
3	SMDB	ONS birth record linked to HES record where baby date of birth differs between them by 24 weeks (168 days) or more		If HES baby date of birth > 24 weeks (168 days) from ONS birth record baby date of birth	The size of the date difference implies a different birth to the same mother<br><br>Kings Project used 30 weeks
4	SMSB	ONS birth record linked to one HES record (originally or remaining)	If ONS birth and HES variables match as listed in Box 3	If ONS birth and HES variables do not match as listed in Box 3	
5	SMSB	ONS birth record linked to >1 HES record all from same spell with genuine epiorder sequence (or a combination of genuine spell and additional delivery records)	If ONS birth and HES variables match as listed in Box 3 (baby date of birth must be exact match) and episode is part of spell and onset of delivery and delivery method and birth status values are valid	If ONS birth and HES variables do not match as listed in Box 3 or are the other episodes in that spell	Identifies where ONS birth records are linked to >1 HES record because some or all form a hospital spell. A spell is identified as where episodes have a sequence of epiorder numbers (e.g. 1 and 2) and the episode start date of the subsequent episode = the episode end date of the previous episode, all have the same admission date, and the discharge date is only recorded on the final episode.<br><br>The episode in the spell with the maximum and valid delivery information is chosen

For peer review only

6	SMSB	ONS birth record linked to >1 HES record with different epikeys but duplicate (admidate, disdate, epistart, epiend, epiorder, date of birth, gestat, birweit, baby sex, numbaby, birstat, birordr, delonset, delmeth, ICD code count, OP code count, procode, sitetret)	If ONS birth and HES variables match as listed in Box 3 and highest epikey	If ONS birth and HES variables do not match as listed in Box 3 or if not highest epikey	Highest epikey is the latest HES record and should contain the most information
7	SMSB	ONS birth record linked to >1 HES record with different epikeys but duplicate (epiorder, date of birth, gestat, birweit, baby sex, numbaby, birstat, birordr, delonset, delmeth, procode, sitetret)<br><br>Different admidate, disdate, epistart, epiend, ICD code count, OP code count	If ONS birth and HES variables match as listed in Box 3 and highest epikey	If ONS birth and HES variables do not match as listed in Box 3 or if not highest epikey	Highest epikey is the latest HES record and should contain the most information<br><br>The additional HES records can be pre- or post- hospital admissions relating to a delivery
8	SMSB	ONS birth record linked to >1 HES record with different epikeys not duplicates	If ONS birth and HES variables match as listed in Box 3 (baby date of birth must be exact match) and ONS and onset of delivery and delivery method and birth status and birth order values are valid	If ONS birth and HES variables match as listed in Box 3 but baby date of birth is not an exact match or HES baby date of birth is null) and onset of delivery and delivery method and birth status and birth order values are not valid and there is a corresponding K record	Chooses the linked HES record with the maximum valid delivery information
10	SMSB	ONS birth record linked to 2 HES records, both match on baby date of birth and location of birth	If ONS birth and HES variables match as listed in Box 3 (HES derived and original baby date of birth must be exact match to ONS) and onset of delivery and delivery method and birth status and birth order values are valid. Most escalated onset of delivery and delivery method	If ONS birth and HES variables match as listed in Box 3 (HES derived baby date of birth is exact match to ONS but HES original baby date of birth is null) and onset of delivery and delivery method and birth status and birth order values are valid (but less escalated)	A small number of records that can be differentiated by one containing maximum delivery information and one doesn't where they both match on location of birth and baby dob

For peer review only

11	SMSB	ONS birth record linked to HES records with differing baby date of birth values (within 14 days of ONS baby date of birth)	If ONS birth and HES variables match as listed in Box 3 (baby date of birth must be exact match) and valid onset of delivery and delivery method value	If HES baby date of birth <=-14 days from ONS baby date of birth or HES baby date of birth <=-1 and >-14 days before ONS baby date of birth<br><br>or<br><br>if already a K record and HES baby date of birth >=1-14 days from ONS baby date of birth	Differentiates between the HES linked records by HES baby date of birth. The HES records that have HES baby date of birth <=-14 days from ONS baby date of birth, then <=-1 and >-14 days before ONS baby date of birth, then >=1-14 days from ONS baby date of birth, are discarded and the remaining 1 linked HES record with HES baby date of birth = ONS baby date of birth and valid method of delivery value is kept (if matching).<br><br>The discarded HES records look like maternal pregnancy care records based on ICD codes
12	SMWB	ONS birth record linked to HES records with differing baby date of birth values (within 15 to 168 days of ONS baby date of birth)	If ONS birth and HES variables match as listed in Box 3 (baby date of birth must be exact match) and valid onset of delivery and delivery method value	If already a K record and HES baby date of birth >14-168 days from ONS baby date of birth and different baby tail values	The discarded records look like different births approximately 2 or 3 months later to the same mother, some of which are clearly premature births.<br><br>Could have been incorporated in Rule 3
13		ONS birth record linked to >1 HES record with different epikeys but duplicate (date of birth, gestat, birweit, baby sex, delonset, delmeth)<br><br>]]	If ONS birth and HES variables match as listed in Box 3 and highest epikey	If not highest epikey	
14	SMSB	Clerical check to identify potential multiple birth records where >1 HES baby tail with same epikey linking to ONS birth record and/or where HES multibirth ICD is true and/or the epikey is linked to >1 ONS birth record, but not due to SMDB	Keep as a singleton matched birth if ONS birth and HES variables match as listed in Box 3<br><br>and<br><br>HES multiple birth ICD but matches only one ONS birth record<br><br>or<br><br>Either ONS birth or HES say multiple or stillbirth, baby tails identical, keep	Identify as potential multiple birth if:<br><br>epikey links >1 ONS birth record with same baby date of birth<br><br>or<br><br>2 different HES baby tails, 1 still birth, and only 1 ONS birth record for livebirth<br><br>or	Any records identified as potential multiple births are separated and given to ONS to check

For peer review only

			only 1 HES record with field order 1 (genuine duplicates)	HES multiple birth ICD and HES baby tails different	
			or		
			ONS birth singleton and HES multiple, treat as singleton if baby tails are the same	D all if ONS birth and HES variables do not match as listed in Box 3	
15	Check	If previous stages have kept > correct number of linked HES records per ONS birth	The highest epikey	Version (s) with earlier or other epikeys	
16	Check	If previous stages have kept no links per ONS birth	If birth location match, ONS baby date of birth within HES admission date ranges, and gestation, birth weight and sex match or are null and match rank 1 and is not a SMDB or invalid record.<br><br>Or if birth location does not match but everything else does		Clerical

For peer review only

2: Multiple Births

RULE	CATEGORY	APPLIES TO	K (Keep the link)	D (Discard the link)	NOTES
1	Cleaning	Linked to HES records that are invalid deliveries		HES records where valid delivery = false	Do not exclude those where HES baby date of birth = null because may be ghost baby tail records
2	Cleaning	Duplicate HES records (typically x 6 or x 9) linked to same ONS birth record where baby tail information and identifier is identical	The first version of the duplicate HES record goes through to the subsequent cleaning stages	All other versions of the duplicate HES record	Due to IT administrative issue with some NHS Trusts
3	SMDB	ONS birth record linked to HES record where baby date of birth differs between them by 24 weeks (168 days) or more		If HES baby date of birth > 24 weeks (168 days) from ONS birth record baby date of birth	The size of the date difference implies a different birth to the same mother<br><br>Kings Project used 30 weeks
4	SMSB	ONS birth records in MULTBTNO linked to one epikey and the correct number of baby tails (originally or remaining)<br><br>Correct number of baby tails<br><br>Correct number of ONS birth records<br><br>1 epikey per MULTBTNO	If ONS birth and HES variables match as listed in Box 3 and field order 1 for 1st baby, field order 2 for 2nd baby etc. so that ONS birth record corresponds to correct baby tail	If ONS birth and HES variables do not match as listed in Box 3.<br><br>Or field and birth orders don't match and sex and birth weight are wrong way round and already 2 correct K records	
5	SMSB	ONS birth records in MULTBTNO linked to one epikey and more than the correct number of baby tails<br><br>> correct number of baby tails<br><br>(duplicates)	If ONS birth and HES variables match as listed in Box 3 and field order 1 (or lowest) for 1st baby, field order 2 (or plus 1) for 2nd baby etc. so that birth record corresponds to correct baby tail	If ONS birth and HES variables do not match as listed in Box 3.<br><br>Or field and birth orders don't match and sex and birth weight are wrong way round and already 2 correct K records	

For peer review only

		Correct number of ONS birth records<br><br>1 epikey per MULTBTNO			
6	SMSB	ONS birth records in MULTBTNO linked to more than one epikey<br><br>Correct number of ONS birth records<br><br>>1 epikey per MULTBTNO	Where epikey matches by baby date of birth, ONS baby date of birth is within HES admission date range, and has valid/most escalated onset of delivery and delivery method	All others	Births with >1 epikey correctly associated with them, but not spells<br><br>Due to first baby linked to 1 epikey and the second baby to the other or duplicates with epikey number increasing by one each time, or non-delivery records
7	SMSB	Remaining MULTBTNO with < correct number of ONS birth records	If ONS birth and HES variables match as listed in Box 3		Some of the missing ONS birth records found in singleton file and unlinked file
8	Check	If previous stages have kept > correct number of links per MULTBTNO. Epikeys are exact duplicates	Version with highest epikey or version within admission date range	Version (s) with earlier or other epikeys	
9	Check	If previous stages have kept no links per MULTBTNO	If birth location match, ONS baby date of birth within HES admission date ranges, and gestation, birth weight and sex match or are null and match rank 1 and is not a SMDB or invalid record<br><br>Or if birth location does not match but everything else does		Clerical

For peer review only

Appendix B

Differences between records with correct and incorrect linkage

Comparison of distributions of key variables for singleton and multiple births in England 2005 to 2014, for 4 groups of data:

1) All birth registrations (n = 6,468,586 singleton births, n = 208,326 multiple births)

2) All birth registrations linked to HES by NHS Digital with a HES delivery record (n = 6,268,013 singleton births, n = 196,769 multiple births)

3) Those of 2) that remain linked to HES after QA (n = 6,138,487 singleton births, n = 193,270 multiple births)

4) Those of 2) left with no link after QA (n = 129,526 singleton births, n = 3,499 multiple births)

Tables B.13 and B.14 are based on total links (a birth registration record can link to >1 HES record):

1) All birth registrations (n = 6,468,586 singleton births, n = 208,326 multiple births)

2) All birth registrations and HES links by NHS Digital (n = 7,862,903 singleton births, n = 395,202 multiple births)

3) Those of 2) that remain linked to HES after QA (n = 6,138,487 singleton births, n = 193,270 multiple births)

4) Those of 2) total broken links after QA (n = 1,724,416 singleton births, n = 201,932 multiple births)

Total number of links are inflated for multiple births because each birth registration within a multiple delivery links with every possible HES baby tail i.e. 4 linked records minimum

Chi-square tests are conducted to test the null hypothesis that the distribution of a demographic variable is the same in the “linked to HES after QA” as in the “No link to HES after QA"

Any rows with values of 0 are excluded from the Chi-square tests

For peer review only

	Singleton births England				Multiple births England				Difference between no link to HES and linked to HES after QA	
	Percentage distribution of variables				Percentage distribution of variables				Singletons	Multiples
**B.1: Match rank**	**All birth registrations**	**All birth registrations linked to HES by NHS Digital with a HES delivery record**	**Linked to HES after QA**	**No link to HES after QA**	**All birth registrations**	**All birth registrations linked to HES by NHS Digital with a HES delivery record**	**Linked to HES after QA**	**No link to HES after QA**		
1	80.51	81.92	82.06	75.13	80.3	81.4	81.4	80.9	-6.93	-0.59
2	2.65	2.67	2.65	3.68	2.0	2.1	2.1	2.8	1.03	0.71
3	1.39	1.42	1.42	1.27	1.3	1.3	1.3	1.1	-0.15	-0.16
4	0.06	0.06	0.06	0.09	0.0	0.0	0.0	0.1	0.03	0.08
5	0.45	0.46	0.46	0.51	0.4	0.4	0.4	0.5	0.06	0.14
6	13.26	13.16	13.05	18.40	14.3	14.4	14.4	13.6	5.35	-0.85
7	0.28	0.27	0.27	0.38	0.3	0.3	0.3	0.1	0.11	-0.25
8	0.05	0.04	0.03	0.53	0.0	0.0	0.0	0.9	0.50	0.93
Null	1.36	0.00	0.00	0.00	1.3	0.0	0.0	0.0	0.00	0.00
**Total**	6,468,586	6,268,013	6,138,487	129,526	208,326	196,769	193,270	3,499		
**χ^2 test**	p < .001 (χ^2 = 11815.13 df = 7)				p < .001 (χ^2 = 903.69, df = 7)					
**B.2: Region of birth**										
East Midlands	6.84	6.95	7.02	3.56	6.61	6.66	6.64	7.66	-3.46	1.02
East of England	10.05	10.08	10.12	8.09	10.50	10.46	10.43	12.35	-2.03	1.92
London	19.36	18.92	18.93	18.34	21.91	21.14	21.03	26.86	-0.59	5.83
North East	4.61	4.66	4.73	0.97	4.62	4.75	4.81	1.54	-3.77	-3.26
North West	13.02	13.18	13.32	6.63	12.35	12.49	12.52	10.89	-6.69	-1.63
South Central	6.92	7.03	7.08	4.56	7.39	7.47	7.47	7.69	-2.52	0.22
South East Coast	7.68	7.76	7.83	4.38	8.48	8.53	8.52	8.72	-3.45	0.19

For peer review only

South West	8.48	8.63	8.73	3.74	8.91	9.17	9.25	4.74	-5.00	-4.51
West Midlands	10.64	10.78	10.93	3.83	10.03	10.05	10.09	7.75	-7.09	-2.35
Yorkshire/Humber	9.69	9.85	9.97	4.07	8.92	9.05	9.07	8.46	-5.90	-0.61
Elsewhere	0.17	0.15	0.10	2.34	0.05	0.04	0.04	0.43	2.24	0.39
Home	2.54	2.01	1.22	39.49	0.23	0.19	0.14	2.92	38.27	2.77
Not known	0.00	0.00	0.00	0.00	0.00	0.00	0	0	0.00	0.00
**Total**	6,468,586	6,268,013	6,138,487	129,526	208,326	196,769	193,270	3,499		
**χ^2 test**	p < .001 (χ^2 = 992568.53, df = 11)				p < .001 (χ^2 = 1768.25, df = 11)					
**B.3: Year of birth**										
2005	9.26	9.02	9.03	8.49	8.82	8.84	8.90	5.57	-0.55	-3.33
2006	9.60	9.40	9.35	11.86	9.38	9.41	9.45	7.55	2.51	-1.90
2007	9.88	9.78	9.70	13.28	9.63	9.79	9.81	8.57	3.57	-1.23
2008	10.14	10.14	10.12	11.12	9.99	10.22	10.29	5.89	1.00	-4.41
2009	10.10	10.15	10.12	11.47	10.56	10.84	10.91	7.23	1.35	-3.68
2010	10.29	10.41	10.45	8.78	10.32	10.69	10.79	5.32	-1.67	-5.47
2011	10.30	10.43	10.46	8.72	10.61	10.77	10.80	9.35	-1.75	-1.45
2012	10.46	10.55	10.56	10.04	10.59	10.25	10.12	17.49	-0.52	7.37
2013	10.01	10.10	10.15	7.94	9.97	9.52	9.40	16.29	-2.21	6.89
2014	9.95	10.02	10.06	8.31	10.13	9.66	9.54	16.75	-1.74	7.21
**Total**	6,468,586	6,268,013	6,138,487	129,526	208,326	196,769	193,270	3,499		
**χ^2 test**	p < .001 (χ^2 = 4645.84, df = 9)				p < .001 (χ^2 = 811.59, df = 9)					
**B.4: Month of birth**										
Jan	8.28	8.27	8.26	8.46	8.35	8.38	8.39	8.09	0.20	-0.30
Feb	7.59	7.58	7.56	8.40	7.57	7.56	7.57	6.97	0.84	-0.60
March	8.29	8.26	8.21	10.19	8.22	8.20	8.17	9.89	1.98	1.72
April	8.00	7.99	8.00	7.56	7.95	7.97	7.94	9.43	-0.44	1.49
May	8.43	8.44	8.45	7.72	8.38	8.38	8.39	7.92	-0.74	-0.48
June	8.27	8.28	8.29	7.91	8.55	8.52	8.52	8.57	-0.38	0.06
July	8.68	8.69	8.70	8.10	8.68	8.70	8.70	8.92	-0.60	0.22
Aug	8.60	8.61	8.62	8.17	8.07	8.14	8.15	7.57	-0.45	-0.58

For peer review only

Sep	8.65	8.67	8.68	8.21	8.39	8.43	8.46	6.89	-0.47	-1.57
Oct	8.67	8.69	8.70	8.24	8.97	8.91	8.91	9.03	-0.46	0.12
Nov	8.21	8.22	8.22	8.08	8.43	8.40	8.41	7.89	-0.14	-0.53
Dec	8.32	8.32	8.30	8.95	8.44	8.39	8.38	8.83	0.65	0.45
**Total**	6,468,586	6,268,013	6,138,487	129,526	208,326	196,769	193,270	3,499		
**χ^2 test**	p < .001 (χ^2 = 1072.06, df = 11)				p < .001 (χ^2 = 42.23, df = 11)					
**B.5: Day of birth (8)**										
Mon	13.25	13.25	13.25	13.27	13.75	13.76	13.74	14.49	0.02	0.75
Tue	14.63	14.64	14.64	14.53	16.41	16.38	16.39	15.72	-0.11	-0.67
Wed	14.90	14.90	14.90	14.86	16.74	16.80	16.80	16.29	-0.04	-0.51
Thur	15.03	15.03	15.04	14.87	17.31	17.29	17.29	17.63	-0.17	0.35
Fri	14.73	14.73	14.73	14.65	16.66	16.65	16.68	15.06	-0.08	-1.62
Sat	12.96	12.95	12.94	13.32	9.77	9.76	9.74	10.95	0.38	1.21
Sun	12.14	12.15	12.15	12.10	7.75	7.75	7.75	8.00	-0.05	0.25
Hol	2.36	2.36	2.36	2.40	1.61	1.62	1.61	1.86	0.04	0.25
**Total**	6,468,586	6,268,013	6,138,487	129,526	208,326	196,769	193,270	3,499		
**χ^2 test**	p < .001 (χ^2 = 36.20, df = 7)				p = .0333 (χ^2 = 15.22, df = 7)					
**B.6: Day of birth (11)**										
Mon	13.05	13.05	13.05	12.99	13.54	13.55	13.54	14.32	-0.07	0.78
Tue	13.32	13.33	13.33	13.26	14.95	14.92	14.93	14.58	-0.06	-0.35
Wed	14.59	14.59	14.59	14.46	16.37	16.43	16.44	16.00	-0.12	-0.43
Thur	14.55	14.56	14.56	14.24	16.78	16.76	16.76	16.95	-0.32	0.19
Fri	13.59	13.59	13.59	13.55	15.39	15.37	15.40	13.63	-0.04	-1.77
Sat	12.96	12.95	12.94	13.47	9.77	9.76	9.74	10.95	0.53	1.21
Sun	12.14	12.15	12.15	12.24	7.75	7.75	7.75	8.00	0.09	0.25
a.hol	1.68	1.68	1.69	1.62	1.89	1.88	1.88	1.60	-0.07	-0.28
b.hol	1.75	1.75	1.75	1.72	1.95	1.96	1.96	2.11	-0.03	0.16
Christmas	0.43	0.43	0.43	0.50	0.29	0.29	0.29	0.37	0.07	0.09
Hol	1.93	1.93	1.93	1.95	1.33	1.33	1.33	1.49	0.03	0.16
**Total**	6,468,586	6,268,013	6,138,487	129,526	208,326	196,769	193,270	3,499		

For peer review only

**χ^2 test**	p < .001 (χ^2 = 60.47, df = 10)				p = .0525 (χ^2 = 18.15, df = 10)					
**B.7: Hour of birth**										
0.00 - 2.59	12.35	12.36	12.35	12.84	8.42	8.46	8.45	9.00	0.49	0.56
3.00 - 5.59	12.15	12.15	12.12	13.75	7.74	7.82	7.81	8.06	1.64	0.25
6.00 - 8.59	10.79	10.76	10.73	12.53	6.67	6.69	6.67	8.03	1.80	1.36
9.00 -11.59	16.06	16.10	16.13	14.39	26.62	26.56	26.53	27.98	-1.74	1.45
12.00 -14.59	12.88	12.87	12.90	11.80	17.35	17.27	17.28	16.95	-1.10	-0.33
15.00 -17.59	11.86	11.85	11.87	10.93	13.41	13.37	13.40	11.77	-0.94	-1.63
18.00 -20.59	11.19	11.18	11.19	11.02	9.65	9.65	9.67	8.49	-0.16	-1.19
21.00 -23.59	11.81	11.81	11.81	11.80	9.18	9.21	9.22	8.69	-0.01	-0.53
Null	0.91	0.91	0.91	0.93	0.96	0.96	0.96	1.03	0.03	0.07
**Total**	6,468,586	6,268,013	6,138,487	129,526	208,326	196,769	193,270	3,499		
**χ^2 test**	p < .001 (χ^2 = 1146.18, df = 8)				p = < .001 (χ^2 = 26.99, df = 8)					
**B.8: Age of mother**										
Under 15	0.03	0.03	0.03	0.02	0.01	0.01	0.01	0.00	-0.01	-0.01
15-19	3.16	3.16	3.18	2.28	1.24	1.26	1.27	0.86	-0.89	-0.41
20-24	18.48	18.57	18.59	17.39	11.14	11.41	11.46	8.75	-1.20	-2.72
25-29	27.29	27.42	27.42	27.44	22.70	23.10	23.19	18.12	0.01	-5.07
30-34	28.72	28.73	28.70	30.39	32.75	32.85	32.83	33.98	1.69	1.16
35-39	16.13	15.99	15.96	17.20	23.75	23.44	23.40	25.81	1.24	2.41
40-44	3.60	3.53	3.54	3.19	5.99	5.64	5.57	9.46	-0.35	3.89
45 & more	0.20	0.19	0.19	0.19	1.34	1.17	1.15	2.14	0.00	0.99
Null	2.39	2.39	2.40	1.91	1.08	1.12	1.12	0.89	-0.49	-0.24
**Total**	6,468,586	6,268,013	6,138,487	129,526	208,326	196,769	193,270	3,499		
**χ^2 test**	p < .001 (χ^2 = 716.36, df = 8)				p < .001 (χ^2 = 196.19, df = 8)					
**B.9: Sex of baby**										
Female	48.69	48.69	48.66	49.65	49.87	49.91	49.93	48.84	0.99	-1.09
Male	51.31	51.31	51.34	50.35	50.13	50.09	50.07	51.16	-0.99	1.09

For peer review only

**Total**	6,468,586	6,268,013	6,138,487	129,526	208,326	196,769	193,270	3,499		
**χ^2 test**	p < .001 (χ^2 = 49.40, df = 1)				p = .2018 (χ^2 = 1.63, df = 1)					
**B.10: Ethnicity of baby**										
Bangladeshi	1.41	1.43	1.43	1.00	0.86	0.89	0.89	0.69	-0.43	-0.20
Indian	3.00	3.02	3.04	1.90	2.73	2.62	2.60	3.63	-1.14	1.03
Pakistani	4.12	4.15	4.18	2.81	2.98	2.99	2.98	3.06	-1.37	0.07
Black African	3.21	3.14	3.12	3.90	3.73	3.67	3.64	5.54	0.77	1.91
Black Caribbean	0.97	0.97	0.97	0.91	1.09	1.09	1.10	0.97	-0.06	-0.13
White British	63.44	63.99	63.97	64.75	65.11	65.85	65.90	62.79	0.78	-3.12
White Other	8.19	8.10	8.13	6.75	8.38	8.21	8.23	7.14	-1.38	-1.08
Other	9.43	9.34	9.35	8.71	8.85	8.65	8.65	8.69	-0.64	0.03
Not Known	6.20	5.87	5.80	9.21	6.26	6.02	5.99	7.40	3.41	1.41
Null	0.02	0.01	0.01	0.07	0.02	0.01	0.01	0.09	0.06	0.08
**Total**	6,468,586	6,268,013	6,138,487	129,526	208,326	196,769	193,270	3,499		
**χ^2 test**	p < .001 (χ^2 = 4730.98 df = 9)				p < .001 (χ^2 = 151.29, df = 9)					
**B.11: Gestational age**										
Missing or less than 22 weeks	0.79	0.78	0.76	1.29	0.93	0.92	0.90	1.83		
									0.53	0.92
Pre-term	6.08	6.07	6.05	7.13	54.10	53.86	53.84	54.93	1.08	1.09
Term	89.17	89.18	89.19	88.63	44.85	45.09	45.12	43.13	-0.56	-2.00
Post-term	3.96	3.97	4.00	2.95	0.13	0.13	0.13	0.11	-1.04	-0.01
**Total**	6,468,586	6,268,013	6,138,487	129,526	208,326	196,769	193,270	3,499		
**χ^2 test**	p < .001 (χ^2 = 1048.57, df = 3)				p < .001 (χ^2 = 35.71, df = 3)					
**B.12: Stillbirth**										
No	99.52	99.53	99.58	97.30	98.87	98.88	98.92	96.46	-2.28	-2.47
Yes	0.48	0.47	0.42	2.70	1.13	1.12	1.08	3.54	2.28	2.47
**Total**	6,468,586	6,268,013	6,138,487	129,526	208,326	205,684	193,270	12,414		

For peer review only

**χ^2 test**	p < .001 (χ^2 = 14053.04, df = 1)				p < .001 (χ^2 = 188.14, df = 1)					
**B.13: Gestational age missing on HES**	**All birth registrations**	**All birth registrations and HES links by NHS Digital**	**Linked to HES after QA**	**Broken links after QA**	**All birth registrations**	**All birth registrations and HES links by NHS Digital**	**Linked to HES after QA**	**Broken links after QA**		
No		68.94	73.50	52.70		74.18	79.73	68.86	-20.80	-10.87
Yes		31.06	26.50	47.30		25.82	20.27	31.14	20.80	10.87
**Total**	6,468,586	7,862,903	6,138,487	1,724,416	208,326	196,769	193,270	3,499		
**χ^2 test**	p < .001 (χ^2 = 272051.09 df = 1)				p < .001 (χ^2 = 6086.08, df = 1)					
**B.14: Birth weight missing on HES**										
No		80.69	83.38	71.12		85.88	89.52	82.40	-12.26	-7.13
Yes		19.31	16.62	28.88		14.12	10.48	17.60	12.26	7.13
**Total**	6,468,586	7,862,903	6,138,487	1,724,416	208,326	395,202	193,270	201,932		
**χ^2 test**	p < .001 (χ^2 = 129812.25 df = 1)				p < .001 (χ^2 = 4814.69, df = 1)					

	**Singleton births England** Percentage distribution of variables				**Multiple births England** Percentage distribution of variables					
**B.15: Location of birth (assigned trust code)**	All birth registrations	All birth registrations linked to HES by NHS Digital with a HES delivery record	Linked to HES after QA	No link to HES after QA	All birth registrations	All birth registrations linked to HES by NHS Digital with a HES delivery record	Linked to HES after QA	No link to HES after QA		
**MMC**	0.00	0.00	0.00	0.00	0.00	0.00	0.00	0.00	0.00	0.00
**MML**	0.07	0.00	0.00	0.04	0.04	0.00	0.00	0.00	0.04	0.00
**MMS**	0.00	0.00	0.00	0.00	0.00	0.00	0.00	0.00	0.00	0.00
**PPJ**	0.04	0.01	0.00	0.26	0.00	0.00	0.00	0.06	0.26	0.06
**PPL**	0.30	0.04	0.00	1.74	0.40	0.05	0.00	2.52	1.74	2.51

For peer review only

For peer review only - http://bmjopen.bmj.com/site/about/guidelines.xhtml

**PPN**	0.00	0.00	0.00	0.01	0.00	0.00	0.00	0.00	0.01	0.00
**PPT**	0.00	0.00	0.00	0.08	0.00	0.00	0.00	0.00	0.08	0.00
**R1F**	0.18	0.18	0.18	0.27	0.18	0.18	0.18	0.31	0.09	0.13
**RA2**	0.48	0.49	0.49	0.21	0.57	0.59	0.60	0.23	-0.28	-0.37
**RA4**	0.22	0.23	0.23	0.11	0.18	0.19	0.18	0.37	-0.12	0.19
**RA7**	0.84	0.86	0.87	0.35	0.84	0.89	0.90	0.23	-0.52	-0.67
**RA9**	0.35	0.35	0.35	0.10	0.33	0.33	0.33	0.11	-0.25	-0.22
**RAE**	0.89	0.91	0.92	0.43	0.66	0.69	0.68	1.00	-0.49	0.32
**RAJ**	0.54	0.55	0.55	0.24	0.57	0.56	0.55	0.86	-0.31	0.31
**RAL**	0.48	0.48	0.49	0.13	0.44	0.44	0.44	0.34	-0.36	-0.10
**RAP**	0.55	0.55	0.55	0.21	0.49	0.49	0.49	0.34	-0.34	-0.15
**RAS**	0.60	0.61	0.62	0.15	0.57	0.59	0.59	0.40	-0.47	-0.19
**RAX**	0.83	0.84	0.85	0.32	0.99	0.93	0.93	1.23	-0.52	0.30
**RBA**	0.47	0.47	0.48	0.27	0.55	0.55	0.55	0.46	-0.21	-0.09
**RBD**	0.30	0.31	0.32	0.04	0.31	0.32	0.33	0.14	-0.28	-0.18
**RBK**	0.61	0.63	0.64	0.13	0.50	0.51	0.51	0.29	-0.50	-0.23
**RBL**	0.52	0.53	0.54	0.14	0.52	0.53	0.53	0.26	-0.40	-0.28
**RBN**	0.50	0.49	0.50	0.08	0.41	0.41	0.41	0.06	-0.43	-0.36
**RBT**	0.42	0.42	0.42	0.45	0.39	0.39	0.39	0.37	0.02	-0.02
**RBZ**	0.23	0.24	0.24	0.10	0.21	0.22	0.22	0.29	-0.14	0.07
**RC1**	0.46	0.46	0.46	0.39	0.48	0.46	0.46	0.60	-0.07	0.14
**RC3**	0.43	0.40	0.40	0.09	0.35	0.33	0.33	0.40	-0.32	0.07
**RC9**	0.74	0.75	0.76	0.50	0.81	0.80	0.80	0.66	-0.25	-0.15
**RCB**	0.49	0.50	0.51	0.05	0.48	0.51	0.51	0.34	-0.46	-0.17
**RCC**	0.25	0.25	0.23	1.04	0.17	0.17	0.16	0.69	0.81	0.52
**RCD**	0.29	0.30	0.31	0.04	0.25	0.26	0.27	0.00	-0.27	-0.27
**RCF**	0.36	0.37	0.38	0.08	0.31	0.32	0.32	0.06	-0.30	-0.27
**RCX**	0.34	0.34	0.35	0.05	0.33	0.34	0.34	0.06	-0.30	-0.29
**RD1**	0.72	0.72	0.72	0.43	0.74	0.75	0.76	0.34	-0.29	-0.42
**RD3**	0.72	0.73	0.74	0.18	0.72	0.74	0.75	0.26	-0.57	-0.49
**RD7**	0.75	0.76	0.78	0.16	0.69	0.69	0.68	0.74	-0.61	0.06
**RD8**	0.55	0.55	0.54	1.02	0.52	0.51	0.50	1.23	0.48	0.73
**RDD**	0.64	0.66	0.67	0.11	0.61	0.64	0.65	0.14	-0.57	-0.51
**RDE**	0.56	0.57	0.57	0.31	0.58	0.58	0.57	0.80	-0.26	0.23
**RDU**	0.72	0.74	0.75	0.31	0.79	0.80	0.80	0.69	-0.43	-0.11

For peer review only

**RE9**	0.22	0.23	0.23	0.05	0.16	0.17	0.17	0.06	-0.18	-0.11
**REF**	0.62	0.64	0.64	0.30	0.59	0.61	0.62	0.34	-0.34	-0.27
**REP**	1.22	1.24	1.26	0.20	1.59	1.64	1.66	0.74	-1.07	-0.91
**RF4**	1.35	1.32	1.35	0.34	1.35	1.31	1.31	1.09	-1.01	-0.23
**RFF**	0.42	0.43	0.43	0.11	0.37	0.38	0.39	0.11	-0.33	-0.27
**RFR**	0.42	0.42	0.42	0.12	0.41	0.41	0.41	0.46	-0.30	0.05
**RFS**	0.43	0.44	0.45	0.09	0.38	0.40	0.41	0.11	-0.36	-0.29
**RFW**	0.64	0.63	0.62	1.13	0.54	0.49	0.47	1.23	0.51	0.76
**RGC**	0.77	0.76	0.77	0.33	0.74	0.72	0.72	0.80	-0.43	0.08
**RGN**	0.64	0.65	0.66	0.11	0.60	0.60	0.60	0.63	-0.54	0.03
**RGP**	0.32	0.33	0.33	0.06	0.29	0.29	0.29	0.17	-0.27	-0.12
**RGQ**	0.54	0.52	0.48	2.48	0.53	0.49	0.46	1.94	2.00	1.48
**RGR**	0.37	0.38	0.39	0.04	0.35	0.36	0.36	0.17	-0.34	-0.19
**RGT**	0.80	0.82	0.84	0.08	1.05	1.10	1.11	0.11	-0.76	-1.00
**RH8**	0.58	0.59	0.60	0.16	0.65	0.67	0.68	0.40	-0.44	-0.28
**RHM**	0.85	0.86	0.87	0.45	0.86	0.87	0.88	0.34	-0.42	-0.54
**RHQ**	1.03	1.05	1.07	0.20	1.16	1.14	1.13	1.74	-0.87	0.62
**RHU**	0.86	0.87	0.88	0.30	0.95	0.99	1.00	0.40	-0.58	-0.60
**RHW**	0.83	0.85	0.86	0.39	0.95	0.99	0.99	0.74	-0.48	-0.25
**RJ1**	0.97	0.98	0.99	0.30	1.24	1.26	1.28	0.49	-0.70	-0.79
**RJ2**	0.55	0.53	0.53	0.46	0.52	0.53	0.53	0.29	-0.07	-0.25
**RJ6**	0.65	0.65	0.65	0.68	0.61	0.61	0.59	1.91	0.03	1.33
**RJ7**	0.74	0.75	0.76	0.26	0.99	1.00	1.01	0.77	-0.50	-0.24
**RJC**	0.40	0.41	0.41	0.14	0.36	0.36	0.36	0.29	-0.27	-0.07
**RJD**	0.31	0.31	0.32	0.08	0.28	0.27	0.28	0.06	-0.24	-0.22
**RJE**	0.85	0.86	0.87	0.29	0.77	0.79	0.80	0.31	-0.58	-0.48
**RJF**	0.54	0.54	0.54	0.46	0.48	0.50	0.50	0.34	-0.09	-0.16
**RJL**	0.69	0.70	0.71	0.46	0.59	0.61	0.61	0.57	-0.24	-0.04
**RJN**	0.30	0.31	0.31	0.02	0.30	0.31	0.32	0.09	-0.29	-0.23
**RJR**	0.47	0.48	0.49	0.08	0.47	0.48	0.49	0.11	-0.41	-0.37
**RJZ**	0.74	0.73	0.73	0.47	1.02	0.97	0.97	0.89	-0.26	-0.09
**RK5**	0.46	0.47	0.48	0.19	0.41	0.43	0.43	0.06	-0.29	-0.38
**RK9**	0.66	0.67	0.66	1.15	0.74	0.72	0.72	1.03	0.49	0.31
**RKB**	0.83	0.85	0.87	0.25	0.89	0.92	0.93	0.11	-0.62	-0.82
**RKE (Whittington)**	0.56	0.48	0.40	4.43	0.59	0.47	0.40	4.34	4.03	3.94

For peer review only

RL4	0.57	0.58	0.59	0.12	0.60	0.62	0.62	0.43	-0.47	-0.19
RLN	0.50	0.50	0.51	0.06	0.44	0.45	0.46	0.00	-0.45	-0.46
RLQ	0.27	0.28	0.29	0.07	0.26	0.25	0.25	0.29	-0.22	0.03
RLT	0.35	0.36	0.36	0.05	0.29	0.31	0.31	0.11	-0.32	-0.20
RLU	1.11	1.10	1.11	0.52	1.32	1.27	1.26	1.91	-0.58	0.66
RM1	0.84	0.86	0.88	0.04	0.82	0.86	0.87	0.20	-0.84	-0.67
RM2	0.56	0.56	0.55	1.04	0.55	0.52	0.50	1.43	0.49	0.93
RM4	0.11	0.11	0.11	0.24	0.08	0.08	0.08	0.17	0.13	0.09
RMC	0.74	0.76	0.78	0.13	0.61	0.63	0.64	0.11	-0.64	-0.53
RMP	0.40	0.41	0.41	0.12	0.31	0.32	0.32	0.17	-0.30	-0.15
RN1	0.41	0.42	0.43	0.16	0.44	0.44	0.44	0.51	-0.27	0.07
RN3	0.63	0.65	0.66	0.17	0.62	0.66	0.66	0.26	-0.49	-0.41
RN5	0.42	0.42	0.43	0.07	0.45	0.48	0.48	0.06	-0.36	-0.43
RN7	0.59	0.60	0.62	0.07	0.52	0.54	0.55	0.11	-0.54	-0.43
RNA	0.68	0.69	0.70	0.21	0.60	0.59	0.60	0.54	-0.50	-0.05
RNH	0.82	0.79	0.78	1.18	0.63	0.61	0.61	0.63	0.40	0.02
RNJ	0.68	0.69	0.70	0.14	0.57	0.57	0.57	0.49	-0.56	-0.09
RNL	0.47	0.46	0.46	0.51	0.42	0.41	0.41	0.66	0.05	0.25
RNQ	0.56	0.57	0.57	0.12	0.54	0.55	0.56	0.09	-0.45	-0.48
RNS	0.65	0.66	0.67	0.13	0.72	0.75	0.75	0.57	-0.54	-0.18
RNZ	0.34	0.35	0.35	0.08	0.43	0.45	0.45	0.06	-0.27	-0.40
RP5	0.79	0.81	0.82	0.13	0.70	0.71	0.71	0.71	-0.69	0.01
RPA	0.68	0.69	0.70	0.25	0.73	0.76	0.76	0.51	-0.45	-0.25
RQ8	0.64	0.64	0.62	1.30	0.70	0.65	0.63	2.17	0.68	1.55
RQM	0.81	0.81	0.82	0.49	1.15	1.11	1.11	1.00	-0.33	-0.11
RQQ	0.36	0.37	0.37	0.09	0.34	0.35	0.35	0.11	-0.28	-0.24
RQW	0.56	0.55	0.56	0.19	0.59	0.57	0.57	0.69	-0.37	0.12
RQX	0.74	0.74	0.73	1.02	0.97	0.97	0.97	0.86	0.29	-0.12
RR1	1.60	1.64	1.66	0.62	1.34	1.38	1.39	0.89	-1.04	-0.50
RR7	0.28	0.28	0.29	0.05	0.29	0.29	0.29	0.29	-0.24	0.00
RR8	1.40	1.38	1.39	0.64	1.47	1.43	1.43	1.34	-0.76	-0.08
RRF	0.45	0.45	0.46	0.20	0.42	0.43	0.43	0.40	-0.26	-0.03
RRV	0.74	0.75	0.76	0.37	1.35	1.35	1.36	0.94	-0.38	-0.42
RTD	0.97	0.98	1.00	0.26	1.25	1.29	1.30	0.57	-0.74	-0.73
RTE	0.93	0.95	0.97	0.11	0.98	1.03	1.04	0.17	-0.86	-0.87

For peer review only

**RTF**	0.47	0.48	0.49	0.08	0.34	0.35	0.36	0.00	-0.42	-0.36
**RTG**	0.84	0.86	0.87	0.32	0.79	0.79	0.78	1.17	-0.55	0.39
**RTH**	1.26	1.28	1.27	1.43	1.56	1.52	1.50	3.06	0.16	1.56
**RTK**	0.59	0.60	0.61	0.07	0.77	0.79	0.81	0.06	-0.54	-0.75
**RTP**	0.64	0.63	0.64	0.31	0.70	0.69	0.69	0.71	-0.33	0.02
**RTR**	0.80	0.80	0.81	0.20	0.91	0.93	0.95	0.17	-0.61	-0.77
**RTX**	0.51	0.52	0.52	0.25	0.41	0.42	0.43	0.23	-0.27	-0.20
**RV8**	0.74	0.75	0.76	0.27	0.61	0.59	0.60	0.49	-0.49	-0.11
**RVJ**	0.87	0.89	0.91	0.21	1.02	1.05	1.06	0.29	-0.70	-0.77
**RVL**	0.96	0.93	0.90	2.27	0.95	0.89	0.87	2.26	1.37	1.39
**RVR**	0.75	0.75	0.76	0.27	0.86	0.85	0.86	0.63	-0.49	-0.23
**RVV**	1.03	1.03	1.02	1.90	1.05	1.00	0.96	3.14	0.89	2.19
**RVW**	0.53	0.53	0.54	0.08	0.51	0.53	0.54	0.06	-0.46	-0.48
**RVY**	0.45	0.44	0.45	0.09	0.39	0.39	0.39	0.57	-0.37	0.18
**RW3**	1.27	1.27	1.28	0.93	1.48	1.43	1.41	2.52	-0.35	1.11
**RW6**	1.52	1.55	1.58	0.24	1.20	1.24	1.25	0.77	-1.34	-0.47
**RWA**	0.84	0.86	0.87	0.16	0.79	0.81	0.82	0.37	-0.71	-0.45
**RWD**	0.86	0.86	0.85	0.97	0.72	0.74	0.73	1.26	0.12	0.53
**RWE**	1.54	1.56	1.57	0.96	1.44	1.42	1.40	2.37	-0.61	0.97
**RWF**	0.76	0.77	0.78	0.33	0.98	1.01	1.02	0.51	-0.45	-0.50
**RWG**	0.82	0.84	0.85	0.33	0.91	0.89	0.88	1.03	-0.52	0.15
**RWH**	0.80	0.80	0.78	1.73	0.94	0.94	0.92	2.00	0.95	1.08
**RWJ**	0.54	0.56	0.57	0.08	0.52	0.55	0.55	0.40	-0.49	-0.15
**RWP**	0.87	0.87	0.89	0.28	0.89	0.83	0.84	0.71	-0.60	-0.12
**RWW**	0.48	0.49	0.50	0.22	0.44	0.45	0.46	0.23	-0.28	-0.23
**RWY**	0.86	0.87	0.88	0.43	0.78	0.79	0.79	0.86	-0.46	0.07
**RX1**	1.50	1.53	1.55	0.77	1.60	1.58	1.57	2.03	-0.78	0.46
**RXC**	0.58	0.59	0.60	0.13	0.49	0.49	0.49	0.54	-0.47	0.06
**RXF**	0.98	1.00	1.02	0.19	0.79	0.83	0.84	0.20	-0.83	-0.64
**RXH**	0.83	0.82	0.83	0.33	1.08	1.07	1.06	1.51	-0.50	0.45
**RXK**	0.89	0.89	0.90	0.43	0.68	0.67	0.66	1.00	-0.47	0.34
**RXL**	0.44	0.45	0.46	0.16	0.39	0.41	0.41	0.17	-0.30	-0.24
**RXN**	0.67	0.68	0.69	0.52	0.61	0.62	0.61	0.74	-0.16	0.13
**RXP**	0.83	0.85	0.86	0.19	0.72	0.74	0.74	0.40	-0.67	-0.34
**RXQ**	0.81	0.82	0.83	0.30	0.78	0.80	0.81	0.29	-0.53	-0.53

For peer review only

**RXR**	0.97	0.96	0.97	0.88	0.82	0.84	0.84	0.69	-0.09	-0.15
**RXW**	0.75	0.77	0.78	0.18	0.76	0.79	0.80	0.46	-0.60	-0.34
**RYJ**	1.43	1.44	1.46	0.47	2.38	2.32	2.33	1.69	-0.99	-0.65
**RYQ**	1.50	1.53	1.55	0.48	1.61	1.67	1.68	0.80	-1.07	-0.88
**RYR**	0.79	0.80	0.80	0.47	0.81	0.80	0.80	0.69	-0.33	-0.11
**XXX**	0.00	0.00	0.00	0.00	0.00	0.00	0.00	0.00	0.00	0.00
**Total**	6,468,586	6,268,013	6,138,487	129,526	208,326	196,769	193,270	3,499		
**χ^2 test**	p < .001 (χ^2 = 6267610.79, df = 151)				p < .001 (χ^2 = 9154.95, df = 151)					

For peer review only

The RECORD statement – checklist of items, extended from the STROBE statement, that should be reported in observational studies using routinely collected health data.

	Item No.	STROBE items	Location in manuscript where items are reported	RECORD items	Location in manuscript where items are reported
**Title and abstract**					
	1	(a) Indicate the study's design with a commonly used term in the title or the abstract (b) Provide in the abstract an informative and balanced summary of what was done and what was found	Study design not stated other than 'quality assuring'. The paper is not about a study, it is about a data quality assurance exercise for the purpose of a larger study which is discussed elsewhere	RECORD 1.1: The type of data used should be specified in the title or abstract. When possible, the name of the databases used should be included.<br><br>RECORD 1.2: If applicable, the geographic region and timeframe within which the study took place should be reported in the title or abstract.<br><br>RECORD 1.3: If linkage between databases was conducted for the study, this should be clearly stated in the title or abstract.	Title gives full data source names.<br><br>Timeframe and country stated in title and abstract<br><br>Title and abstract specify linkage
**Introduction**					
Background rationale	2	Explain the scientific background and rationale for the investigation being reported			Not applicable to the article on its own. It is part of a larger study and these aspects are described in the main study report. The purpose of this paper is given in the Introduction on page 4.
Objectives	3	State specific objectives, including any prespecified hypotheses			As above, described in Chapter 1of main study report.

					The objectives of this paper are given in the Introduction on page 4.
**Methods**					
Study Design	4	Present key elements of study design early in the paper			N/A to this paper on its own. Described in separate main study report
Setting	5	Describe the setting, locations, and relevant dates, including periods of recruitment, exposure, follow-up, and data collection			N/A to this paper on its own. Described in separate main study report
Participants	6	*(a) Cohort study* - Give the eligibility criteria, and the sources and methods of selection of participants. Describe methods of follow-up<br>*Case-control study* - Give the eligibility criteria, and the sources and methods of case ascertainment and control selection. Give the rationale for the choice of cases and controls<br>*Cross-sectional study* - Give the eligibility criteria, and the sources and methods of selection of participants<br><br>*(b) Cohort study* - For matched studies, give matching criteria and number of exposed and unexposed<br>*Case-control study* - For matched studies, give matching criteria	N/A. The paper discusses quality assurance of all linked data for births in England 2005-2014<br><br>N/A	RECORD 6.1: The methods of study population selection (such as codes or algorithms used to identify subjects) should be listed in detail. If this is not possible, an explanation should be provided.<br><br>RECORD 6.2: Any validation studies of the codes or algorithms used to select the population should be referenced. If validation was conducted for this study and not published elsewhere, detailed methods and results should be provided.<br><br>RECORD 6.3: If the study involved linkage of databases, consider use of a flow diagram or other graphical display to demonstrate the data linkage process, including the number of individuals with linked data at each stage.	N/A to this paper on its own. Described in separate main study report<br><br>N/A to this paper on its own. Described in separate main study report and in Dattani's preceding paper on linkage<br><br>N/A. This paper works with all births in England that linked to HES maternity delivery

		and the number of controls per case			records by NHS Digital. Dattani's preceding paper discusses the linkage process
Variables	7	Clearly define all outcomes, exposures, predictors, potential confounders, and effect modifiers. Give diagnostic criteria, if applicable.		RECORD 7.1: A complete list of codes and algorithms used to classify exposures, outcomes, confounders, and effect modifiers should be provided. If these cannot be reported, an explanation should be provided.	N/A to this paper on its own. Described in separate main study report. This is not a statistical analysis of a selected population with outcomes.
Data sources/ measurement	8	For each variable of interest, give sources of data and details of methods of assessment (measurement). Describe comparability of assessment methods if there is more than one group			Data quality on page 6-7
Bias	9	Describe any efforts to address potential sources of bias			Page 12
Study size	10	Explain how the study size was arrived at			Maximised by starting with earliest year for which data were available and ending with most recent at time.
Quantitative variables	11	Explain how quantitative variables were handled in the analyses. If applicable, describe which groupings were chosen, and why			N/A to this paper on its own. Described in separate main study report.
Statistical methods	12	(a) Describe all statistical methods, including those used to			a) N/A to this paper on its own.

For peer review only

		control for confounding<br><br>(b) Describe any methods used to examine subgroups and interactions<br><br>(c) Explain how missing data were addressed<br><br>(d) *Cohort study* - If applicable, explain how loss to follow-up was addressed<br>*Case-control study* - If applicable, explain how matching of cases and controls was addressed<br>*Cross-sectional study* - If applicable, describe analytical methods taking account of sampling strategy<br><br>(e) Describe any sensitivity analyses			Described in separate main study report.<br><br>b) N/A to this paper on its own. Described in separate main study report.<br><br>c) page 6-7 'Data quality'<br><br>d) N/A to this paper on its own. Described in separate main study report.<br><br>e) N/A to this paper on its own. Described in separate main study report.
Data access and cleaning methods		..		RECORD 12.1: Authors should describe the extent to which the investigators had access to the database population used to create the study population.<br><br>RECORD 12.2: Authors should provide information on the data cleaning	P17 'Approvals'. And use of secure setting, the VML, described throughout.<br><br>The purpose of the entire paper is to

For peer review only - http://bmjopen.bmj.com/site/about/guidelines.xhtml

				methods used in the study.	describe the cleaning/quality assuring method of the linked data
Linkage		..		RECORD 12.3: State whether the study included person-level, institutional-level, or other data linkage across two or more databases. The methods of linkage and methods of linkage quality evaluation should be provided.	Page 5 'Linkage' gives a summary of the linkage, and states that it is person-level. This is described in more detail in Dattani's preceding paper
**Results**					
Participants	13	(a) Report the numbers of individuals at each stage of the study (*e.g.*, numbers potentially eligible, examined for eligibility, confirmed eligible, included in the study, completing follow-up, and analysed)<br>(b) Give reasons for non-participation at each stage.<br>(c) Consider use of a flow diagram		RECORD 13.1: Describe in detail the selection of the persons included in the study (*i.e.,* study population selection) including filtering based on data quality, data availability and linkage. The selection of included persons can be described in the text and/or by means of the study flow diagram.	Tables 1a and 1b page 6 and page 20 gives the numbers of records included. All linked records were included. Linkage is described in Dattani's preceding paper.
Descriptive data	14	(a) Give characteristics of study participants (*e.g.*, demographic, clinical, social) and information on exposures and potential confounders<br>(b) Indicate the number of participants with missing data for each variable of interest<br>(c) *Cohort study* - summarise			a) N/A<br>b) Table 2 page 7

For peer review only

		follow-up time (*e.g.*, average and total amount)			c) N/A
Outcome data	15	*Cohort study* - Report numbers of outcome events or summary measures over time<br>*Case-control study* - Report numbers in each exposure category, or summary measures of exposure<br>*Cross-sectional study* - Report numbers of outcome events or summary measures			N/A
Main results	16	(a) Give unadjusted estimates and, if applicable, confounder-adjusted estimates and their precision (e.g., 95% confidence interval). Make clear which confounders were adjusted for and why they were included<br>(b) Report category boundaries when continuous variables were categorized<br>(c) If relevant, consider translating estimates of relative risk into absolute risk for a meaningful time period			N/A, but quality assurance results given page 10
Other analyses	17	Report other analyses done—e.g., analyses of subgroups and interactions, and sensitivity analyses			Page 11 to 13
**Discussion**					
Key results	18	Summarise key results with reference to study objectives			Page 14 'Main findings'
Limitations	19	Discuss limitations of the study, taking into account sources of potential bias or imprecision. Discuss both direction and magnitude of any potential bias		RECORD 19.1: Discuss the implications of using data that were not created or collected to answer the specific research question(s). Include discussion of misclassification bias,	N/A, but implications of the results given page 15

For peer review only

				unmeasured confounding, missing data, and changing eligibility over time, as they pertain to the study being reported.	
Interpretation	20	Give a cautious overall interpretation of results considering objectives, limitations, multiplicity of analyses, results from similar studies, and other relevant evidence			Page 15 'Implications'
Generalisability	21	Discuss the generalisability (external validity) of the study results			Page 15 'Implications'. It is clear that the article relates to England but findings about trusted third party linkage could apply elsewhere.
**Other Information**					
Funding	22	Give the source of funding and the role of the funders for the present study and, if applicable, for the original study on which the present article is based			Source of funding given on page 1
Accessibility of protocol, raw data, and programming code		..		RECORD 22.1: Authors should provide information on how to access any supplemental information such as the study protocol, raw data, or programming code.	Data sharing statement given on page 1

*Reference: Benchimol EI, Smeeth L, Guttmann A, Harron K, Moher D, Petersen I, Sørensen HT, von Elm E, Langan SM, the RECORD Working Committee. The REporting of studies Conducted using Observational Routinely-collected health Data (RECORD) Statement. *PLoS Medicine* 2015; in press.

*Checklist is protected under Creative Commons Attribution (CC BY) license.

For peer review only - http://bmjopen.bmj.com/site/about/guidelines.xhtml
